# Neural Ganglia Transcriptome and Peptidome Associated with Sexual Maturation in Female Pacific Abalone (*Haliotis discus hannai*)

**DOI:** 10.3390/genes10040268

**Published:** 2019-04-02

**Authors:** Mi Ae Kim, Kesavan Markkandan, Na-Young Han, Jong-Moon Park, Jung Sick Lee, Hookeun Lee, Young Chang Sohn

**Affiliations:** 1 Department of Marine Molecular Bioscience, Gangneung-Wonju National University, Gangneung 25457, Korea; kimmiaecho@gmail.com; 2 The East Coast Research Institute of Life Science, Gangneung-Wonju National University, Gangneung 25457, Korea; 3 TheragenETEX Bio Institute, TheragenETEX Inc., Suwon 16229, Korea; kesavanmarkkandan@gmail.com; 4 College of Pharmacy, Gachon University, Incheon 21936, Korea; nyhan1103@daum.net (N.-Y.H.); bio4647@naver.com (J.-M.P.); hklee@gachon.ac.kr (H.L.); 5 Department of Aqualife Medicine, Chonnam National University, Yeosu 59626, Korea; ljs@chonnam.ac.kr

**Keywords:** ganglia, gastropod, HCD-MS/MS spectra, maturation-associated neuropeptide, RNA-Seq

## Abstract

Genetic information of reproduction and growth is essential for sustainable molluscan fisheries and aquaculture management. However, there is limited knowledge regarding the reproductive activity of the commercially important Pacific abalone *Haliotis discus hannai*. We performed de novo transcriptome sequencing of the ganglia in sexually immature and mature female Pacific abalone to better understand the sexual maturation process and the underlying molecular mechanisms. Of the ~305 million high-quality clean reads, 76,684 transcripts were de novo-assembled with an average length of 741 bp, 28.54% of which were annotated and classified according to Gene Ontology terms. There were 256 differentially expressed genes between the immature and mature abalone. Tandem mass spectrometry analysis, as compared to the predicted-peptide database of abalone ganglia transcriptome unigenes, identified 42 neuropeptide precursors, including 29 validated by peptidomic analyses. Label-free quantification revealed differential occurrences of 18 neuropeptide families between immature and mature abalone, including achatin, FMRFamide, crustacean cardioactive peptide, and pedal peptide A and B that were significantly more frequent at the mature stage. These results represent the first significant contribution to both maturation-related transcriptomic and peptidomic resources of the Pacific abalone ganglia and provide insight into the roles of various neuropeptides in reproductive regulation in marine gastropods.

## 1. Introduction

The reproductive success of animals depends on diverse physiological and behavioral processes that take place in a timely and orderly manner. In addition to the importance of successful coordination among these processes, each step requires continuous adjustment according to the external factors of abiotic and biotic natures. In both vertebrates and invertebrates, neuroendocrine factors, such as neurotransmitters and neurohormones, are responsible for communicating cell signals, and they thus play crucial roles in the regulation, formation, maturation, and release of gametes [1,2]. Bioactive neuropeptides (3–40 amino acids) are produced from larger precursor proteins (prepropeptides) via a series of sequence-specific and tissue-specific proteolytic steps, which are frequently associated with other covalent post-translational modifications.

In vertebrates, the hypothalamic gonadotropin-releasing hormone (GnRH) is a representative neuropeptide in pituitary gonadotropin synthesis, controlling pulsatile secretion, modulating gonadal steroid feedback, and bringing about full fertility [3]. In contrast to the hypothalamic regulation of the brain, the neural ganglia of ecdysozoan invertebrates produces several neuropeptides, which include insulin-like peptides, neuroparsins, neuropeptide F (NPF)/short NPF, allatoregulatory peptides, corticotropin-releasing factor-like diuretic hormone, gonad-stimulating factor, crustacean hyperglycemic hormone, vitellogenesis inhibiting hormone, and gonad inhibitory hormone, which have been reported to act as reproductive regulators [4,5].

Like other animals, mollusks produce several neuropeptides from the neural ganglia, which aid in the control of the reproduction processes. For example, the egg-laying hormone is a 36-amino-acid neuropeptide that is expressed in specific neuron clusters that belong to the cerebral ganglia [6,7]. Moreover, the caudo-dorsal cell hormones, APGWamide, and FMRFamide, act as triggers in various aspects of reproductive activities, such as penis erection, inhibition of the contraction of the oviduct, and stimulation of secretion of the egg capsule and the oviducal gland [6,7,8,9,10]. Transcriptome resources for reproduction-related genes are currently available in several mollusks, including the freshwater snail (*Lymnaea stagnalis*) [11], the Sydney rock oyster (*Saccostrea glomerata*) [12], and the cuttlefish (*Sepia officinalis*) [13]. In addition, detailed information for some mollusks about neuropeptides and their potential reproductive functions is now being studied [11,12,13,14,15,16].

Over the past few decades, supplies of abalone from fisheries have been declining worldwide, which is mainly due to over-exploitation, illegal harvesting, disease, and habitat degradation, although a significant increase of abalone production through aquaculture in the last 10 years was reported in China and Korea [17]. In Korea, the Pacific abalone *Haliotis discus hannai* (Hdh) is a commercially valuable marine gastropod, and it has been studied from various perspectives, including genetics [18], breeding [19], immunology [20], and growth [21]. Recently, transcriptome and proteome analyses of abalone have been conducted to identify the reproductive and metabolic genes, such as vitellogenin, forkhead box protein L2, sperm-associated antigen 6, Fem-1 homolog C-like, Teckin-1, heat shock cognate 70, and peroxiredoxin 6 [22,23]. In addition, the putative abalone neuropeptides that were involved in the regulation of sexual maturation and spawning were reported, including GnRH [24], a Kazal-type proteinase inhibitor [25], APGWamide [26,27], myomodulin, whitnin, FMRFamide, a schistosomin-like peptide, molluscan insulin-related peptide, and haliotid growth-associated peptide [14]. However, the detailed mechanisms that orchestrate the entire reproductive process have not yet been elucidated, and no systematic identification of neuropeptides that are involved in sexual maturation has been performed in abalone species.

In the present study, we performed de novo transcriptome and peptidome analyses of the neural ganglia from sexually immature and mature female Hdh. The objectives of this study were (1) to establish a novel transcriptomic dataset of the neural ganglia from female Hdh, (2) to identify differentially expressed genes and pathways that are closely related to the regulation of gamete maturation, and (3) to further provide a valuable resource for the function of neuropeptide families while using peptidomic analysis. Overall, our results highlight the key neuropeptide-related genes that are differentially expressed during sexual maturation in abalone, which were mainly found to play roles in steroidogenesis, ovarian development, neuro-protection, and metabolism in vertebrates. Thus, the identification of these key genes and pathways, which are essential for the reproductive function, can be used to improve aquaculture and to develop targeted breeding strategies for abalone.

## 2. Materials and Methods

### 2.1. Animals and Tissue Collection

Adult female Pacific abalones were obtained from a public fish market (Gangwon-do, Gangneung, Korea). The reproductive cycle of the ovaries and neural ganglia were determined based on a previous report [24]. The cerebral ganglion (CG), pleuro-pedal ganglion (PPG), and branchial ganglion (BG) tissues were dissected from immature and mature individuals: 1) immature (stage VI: inactive stage, January 2017, shell length (SL) 83.67 ± 4.04 mm, body weight (BW) 81.01 ± 18.63 g, *n* = 7); and, 2) mature (stage III: ripe stage, August 2016, SL 88.67 mm ± 7.09 mm, BW 84.11 ± 22.15 g, *n* = 7). For transcriptomic analysis, the whole ganglia (CG + PPG + BG) were dissected from three immature and three mature female abalones, which were immediately frozen in liquid nitrogen and stored at −80 °C until total RNA isolation. For the ganglia peptidome, the CG, PPG, and BG were individually collected from four immature and four mature females. Pooled CG, PPG, or BG tissue samples at two different physiological stages (three samples at two time points) were used for peptide extraction.

### 2.2. mRNA Library and Sequencing

Total RNA was extracted from the whole ganglia, including the CG, PPG, and BG, while using RNeasy Lipid Tissue Kit (Qiagen, Hilden, Germany) according to the manufacturer’s instructions after treatment with RNase-free DNase I (Qiagen) to eliminate genomic DNA. The concentration and integrity of the RNA were assessed with a Nanodrop spectrophotometer (8000, Thermo Fisher Scientific, Waltham, MA, USA) and an Agilent 2100 Bioanalyzer (Agilent Technologies, Waldbronn, Germany), respectively. The RNA samples with an optical density ratio at 260/280 nm ≥ 1.8 and an RNA integrity number ≥7.0 were used in subsequent experiments. Equal amounts of high-quality RNA were then separately used for cDNA synthesis and sequencing. The cDNA library was prepared with ~1.0 μg of total RNA, following the manufacturer’s recommendations of TrueSeq RNA library Preparation Kit (Illumina Inc., San Diego, CA, USA). The library was then amplified and the final library yielded ~200 ng of cDNA, with an average fragment size of ~300 bp. The resulting cDNA libraries were then paired-end sequenced (2 × 101 bp) to produce four-million reads using the HiSeq 2500 platform (Illumina Inc., San Diego, CA, USA). The raw reads of immature and mature ganglia of Hdh were submitted to the NCBI Sequence Read Archive under Accession No. SRP163160.

### 2.3. De novo Assembly and Annotation

Before assembly, the raw reads of fastq format were subjected to pre-processing by removing adapter sequences, duplicates, and ambiguous nucleotides using FastQC [28]. Reads that contained more than five nucleotides with quality scores of less than 20 were trimmed. The resulting high-quality clean data were used in subsequent downstream analyses. The pre-processed high-quality sequences were then subjected to de novo assembly while using Trinity software [29]. The default assembly parameters were used with a minimum overlap length of 50 bp and minimum sequence identity of 90%. Functional annotations were conducted by comparing the sequences against those in public databases. The Gene Ontology (GO) terms were assigned to each unigene based on the GO terms annotated to its corresponding homologs. Unigenes were classified according to GO terms within molecular functions, cellular components, and biological processes categories, and they were further plotted using Web Gene Ontology Annotation Plot (WEGO) software with default parameters. Finally, unigenes were assigned to specific biochemical pathways according to the KEGG database using BLASTx, followed by retrieving KEGG orthology information.

### 2.4. Differentially Expressed Genes (DEGs) Analysis

To obtain an overview of the expression pattern of the identified unigenes in all samples, reads from individual samples were aligned to the reference transcriptome using Bowtie2 [30] with default parameters. Transcript abundances in reads per kilobase per million reads mapped (RPKM) were estimated while using the RNA-Seq by Expectation Maximization (RSEM) algorithm. To identify the differential expression patterns of transcripts, the trimmed mean of M-values normalized fragments per kilobase of transcript per million reads mapped (FPKM = total exon fragments/(mapped reads (millions) × exon length (kb))) matrix was used for generating heatmaps in the R programming environment [31]. Differential expression was assessed using Cuffdiff2 [32]. Unigenes were considered to be differentially expressed at a *p*-value < 0.05, following a Benjamini and Hochberg false discovery rate (FDR) adjustment of 5% (0.05). Output files of DEGs (FDR  <  0.05) were then used in Gene Set Enrichment Analysis [33] to evaluate the relationship between gene expression patterns that are significantly associated with reproduction. In this study, we focused only on the quantification of known mRNA sequences; therefore, any unknown proteins or isoforms were not considered.

### 2.5. Sample Preparation and Peptide Extraction

The ganglia tissues were subjected to heat at 95 °C in an air-evacuated cartridge (Denator T1 Heat Stabilizor, Denator AB, Uppsala, Sweden) for stabilizing the peptides of tissue samples. The tissue was pulverized by Covaris cryoPREPTM (Covaris Inc., Woburn, MA, USA) and then sonicated on a focused ultrasonicator (Covaris S-Series, Covaris Inc., Woburn, MA, USA) with Adaptive Focused AcousticsTM in 8 M urea for extraction of the proteome. After extraction, the six ganglia samples were separated into three equivalent technical replicate samples, resulting in 18 total samples for analysis (three samples × two time points × three technical replicate samples). The protein concentration of each sample was measured with a bicinchoninic acid assay (Pierce, Rockford, IL, USA). Each 100 µg protein sample was centrifuged at 16,000 × *g*, 30 min, 4 °C, and the supernatant was collected into a new Eppendorf tube. Microcon YM-30 cutoff filters (Millipore, Milford, MA, USA) were pre-rinsed with 100 µL of 20% acetonitrile (MeCN) and 30% MeOH (15,000 × *g*, 8 min) twice. The supernatant was centrifuged (15,000 × *g*, 20 min, 4 °C) in the 30 kDa filters and the flow-through was collected. After adding 1% trifluoroacetic acid (TFA), the solution was desalted in a C18 microspin column (Harvard Apparatus, Holliston, MA, USA) that was preconditioned with 100 µL MeOH, 100 µL 80% MeCN, 0.5% AcOH, 100 µL 1% TFA, and 3% MeCN, twice. The C18 columns were then washed twice with 100 µL 8% MeCN, 0.5% AcOH, and 100 µL 0.5% AcOH. The sample solution was loaded onto the C18 column and they peptides were eluted with 100 µL 40% MeCN, 0.5% AcOH, and then with 100 µL 50% MeCN and 0.5% AcOH. The eluate was collected into a new Eppendorf tube for LC-MS/MS.

### 2.6. LC-MS/MS

The eluted peptide solution was dried out by vacuum centrifugation in a speed-vac. The peptides were reconstituted with 100 µL of 2% MeCN, 0.5% AcOH, and 0.1% TFA. The sample solution was analyzed by online reversed-phased C18 nanoscale LC-MS/MS on a Q-Exactive mass spectrometer (Thermo Fisher Scientific) while using a data-dependent analysis approach. The LC-MS/MS analysis was performed with a nanoflow UHPLC system (UltiMate 3000 UHPLC, Dionex, Sunnyvale, CA, USA) that was connected through a nano-electrospray ion source to the mass spectrometer. The peptides were separated by a linear MeCN gradient for 60 min in a PepMapTM RSLC C18 column (2 µm, 100 Å, 75 µm × 50 cm, Thermo Fisher Scientific). Full-scan MS spectra were acquired from 400–2000 m/z at a target value of 3e6 and a resolution of 70,000, and the higher-energy collision dissociation (HCD)-MS/MS spectra were recorded at a target value of 1 × 10^5^, with a resolution of 17,500 using a normalized collision energy of 27.

### 2.7. Peptide Identification and Quantification by MaxQunat

The raw MS data files were processed using MaxQuant software (ver. 1.5.8.3, Max-Planck Institute of Biochemistry, Department of Proteomics and Signal Transduction, Munich, Germany). Peptides were identified by searching all MS/MS spectra against an abalone TBI unigene of ganglia protein sequence database (Figure S1). The HCD-MS/MS spectra were searched with variable post-translational modifications of methionine oxidation and dioxidation, C-carbamylation, N-terminal pyroglutamate, C-terminal amidation, and no enzyme specificity being required. The search parameters were set to an initial precursor ion tolerance of 20 ppm and an MS/MS tolerance at 20 ppm. To reduce the peptide complexity for the identification of biologically relevant peptides, data filtering and elimination of protein redundancy were finally adopted to MS data according to Secher’s algorithm [34]. Label-free peptide quantification that was based on peak intensities and validation was performed with Perseus software (ver. 1.5.8.5, Max-Planck Institute of Biochemistry, Planegg, Germany), which required a minimum FDR threshold of 1%.

### 2.8. Neuropeptide Prediction

Molluscan neuropeptide precursors that were reported in the literatures [15,35,36,37] were input into TBLASTN as query sequences (Supplementary File 1) to identify the orthologous neuropeptides that were identified in the Hdh transcriptome database. All of the hits returned by a given search were fully translated using the online open reading frame (ORF) finder tool of the National Center for Biotechnology Information (Bethesda, MD, USA; https://www.ncbi.nlm.nih.gov/orffinder/) and then input into BLASTp as query sequences to identify the neuropeptide orthologues. Each of the deduced precursor proteins was assessed for the presence of a signal peptide using the online program SignalP 4.1 (http://www.cbs.dtu.dk/services/SignalP/); the D-cutoff was set to “Default” [38]. NeuroPred (http://stagbeetle.animal.uiuc.edu/cgi-bin/neuropred.py) was then used to predict the cleavage sites [39], and the presence of putative bioactive peptides was determined by homology to known molluscan preprohormone processing schemes [15,35,40].

### 2.9. Quantitative Real-Time RT-PCR

Quantitative real-time RT-PCR (qPCR) was performed for genes of *NPF, FxRIamide*, *Achatin,* crustacean cardioactive peptide (*CCAP*), *FMRFamide,* and *pedal peptides A* and *B*. Based on the minimum information for publication of qPCR experiments guidelines [41], the transcript levels were validated. Total RNAs were extracted from the CG and PPG of sexually immature (stage VI, BW 87.76 ± 15.26 g, *n* = 4) and mature female abalone (stage III, 79.14 ± 8.70 g, *n* = 4) using the RNeasy Mini kit (Qiagen, Hilden, Germany). After the digestion of genomic DNA, 1 μg of total RNA was reverse transcribed while using the PrimeScript RT reagent (Perfect Real Time, Takara, Japan). The resulting cDNAs were diluted and an amount equivalent to 10 ng of starting RNA was assayed for mRNA expression analysis using ribosomal protein 5 (*RPL5*) as the reference gene. SYBR-based qPCR reactions (SYPR Premix Ex Taq II, Takara, Japan) were performed on Applied Biosystems 7500 Real-Time PCR System (Applied Biosystems, Foster City, CA, USA) using the following reaction conditions: 50 °C for 2 m, 95 °C for 10 m, followed by 40 cycles of 95 °C for 15 s, and 60 °C for 1 m. Table S1 lists the primer sets that were used in this study. The PCR efficiencies of the target gene and reference gene were verified. The relative mRNA expression was calculated according to the formula: 2^−(Ct target gene−Ct reference gene)^. All of the results are expressed as the mean ± SEM.

## 3. Results

### 3.1. Transcriptome Assembly and Annotation

**Figure 1:**
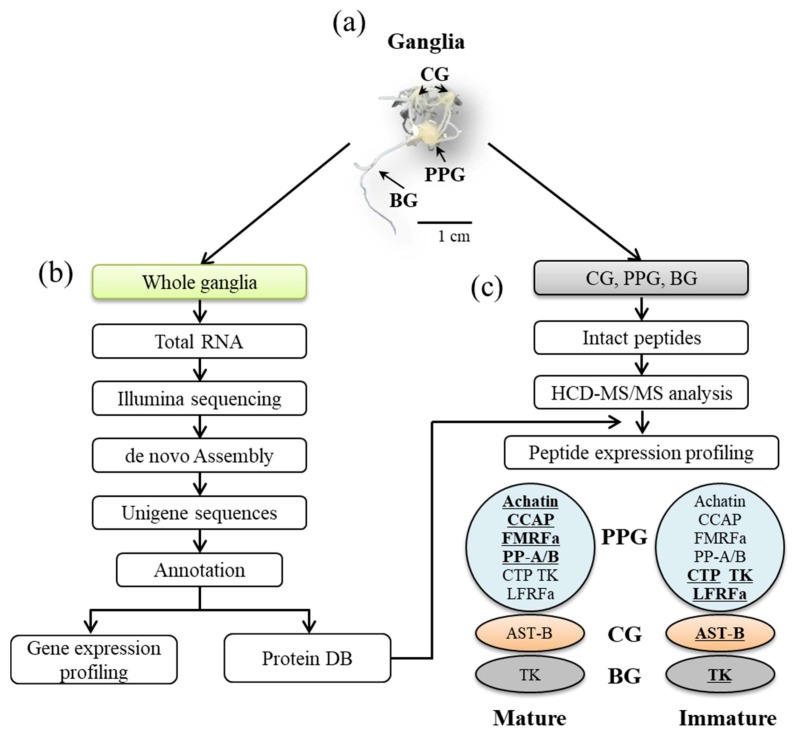
Workflow of transcriptomics and peptidomics experiments to identify neuropeptides of immature and mature female *Haliotis discus hannai* and a summary of the results in the present study. (**a**) Ganglia of abalone, cerebral ganglion (CG), pleuro-pedal ganglion (PPG), and branchial ganglion (BG); (**b**) Differentially expressed genes (DEGs) between mature and immature stages by transcriptome analysis; and, (**c**) Overview of increased (bold and underlined) or decreased neuropeptides by peptidome analysis.

**Figure 2:**
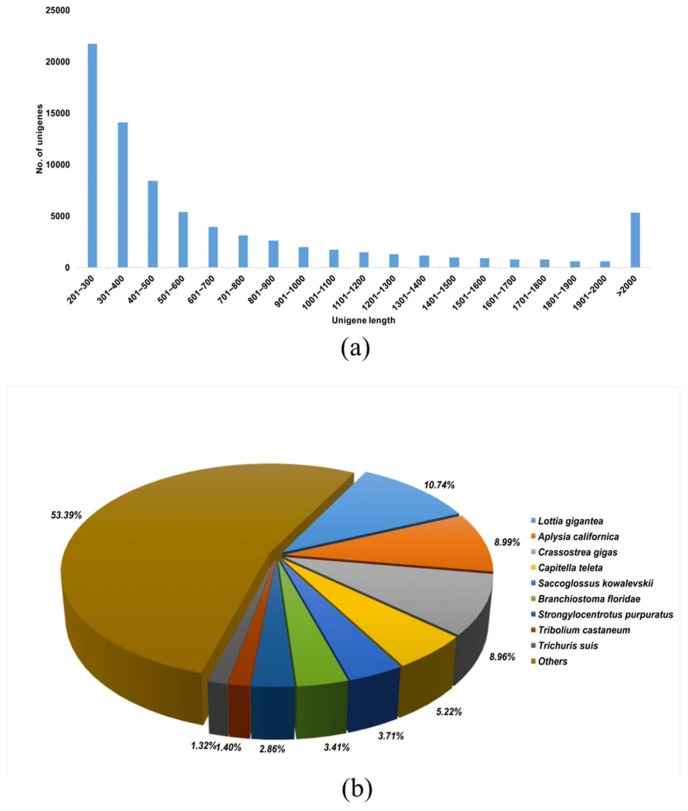
Overview of the *Haliotis discus hannai* transcriptome. (**a**) Overall length distribution of unigene reads; (**b**) Species distribution of the best of BLASTx hits of transcripts against the NCBI NR database.

Six cDNA libraries from Hdh at immature and mature stages were constructed for Illumina sequencing. Figure 1 schematically illustrates the overall procedure that was applied in this study and Table 1 summarizes the data processing results. Using Trinity de novo assembly [29] of the reads into transcriptome sequences, 76,684 unique transcript fragments (unigenes) were obtained from the six libraries. The length of these unigenes ranged from 201 to 11,864 bp, with a total length of 56,857,768 bp and an average length of 741 bp (Table 1, Figure 2A). Further, to provide information for the reference transcripts, the unigene sequences were selected and then subjected to annotation analysis by matching sequences against the non-redundant (NR) and Swiss-Prot databases while using the BLASTx search tool with an E-value of 1.0 × 10^−5^ (Table S2). The total number (21,891) of transcripts that were annotated in Swiss-Prot and NR was then used as a basis for the species distribution percentages. Among the annotated unigenes, 2503 (10.74%) were most similar to orthologs of *Lottia gigantea*, followed by *Aplysia californica* (8.99%), *Crassostrea gigas* (8.96%), *Capitella teleta* (5.22%), and other species (53.39%) (Figure 2B).

**Figure 3:**
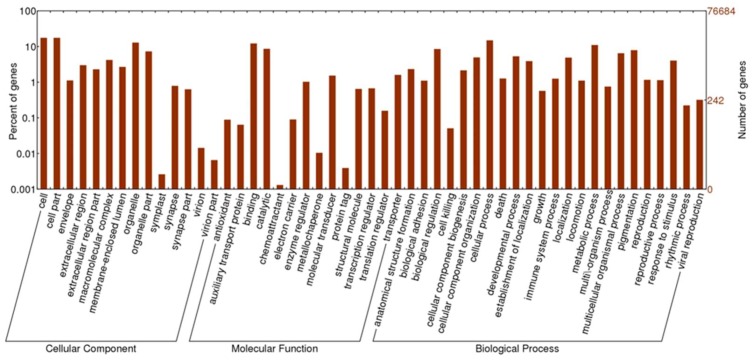
Functional annotation of assembled sequences based on Gene Ontology (GO) categorization. The histogram of the GO annotation was generated automatically using the WEGO tool based on the most recent GO archive. GO analysis was performed at level 2 for three main categories (cellular component, molecular function, and biological process). The right y-axis indicates the number of unigenes in a category. The left y-axis indicates the percentage of a specific category of genes in that main category. One gene could be annotated under more than one GO term.

Among the NR unigenes, 15,585 could be assigned as a GO functional classification term, and these genes were subsequently categorized into 51 functional groups (Figure 3). Specifically, GO analysis assigned 9864 unigenes to biological process, 6364 to cellular component, and 2404 to molecular function, with "cellular process", "cell part", and "binding", being the most prevalent terms for each category, respectively. Other genes were found to be involved in various other principal biological processes, such as metabolic process, biological regulation, developmental process, pigmentation, and localization. The corresponding pathways of the assembled unigenes were determined by comparison to the Kyoto Encyclopedia of Genes and Genomes (KEGG) database while using BLASTx. In total, 3419 unigenes were assigned to the KEGG pathways, resulting in 175 metabolic pathways, with particular enrichment in various metabolisms and signaling pathways (Table S3). These annotations provide a valuable resource for investigating specific processes, structures, functions, and pathways in Hdh related to sexual maturation.

### 3.2. DEGs in the Ganglia of Immature and Mature Abalone

**Figure 4:**
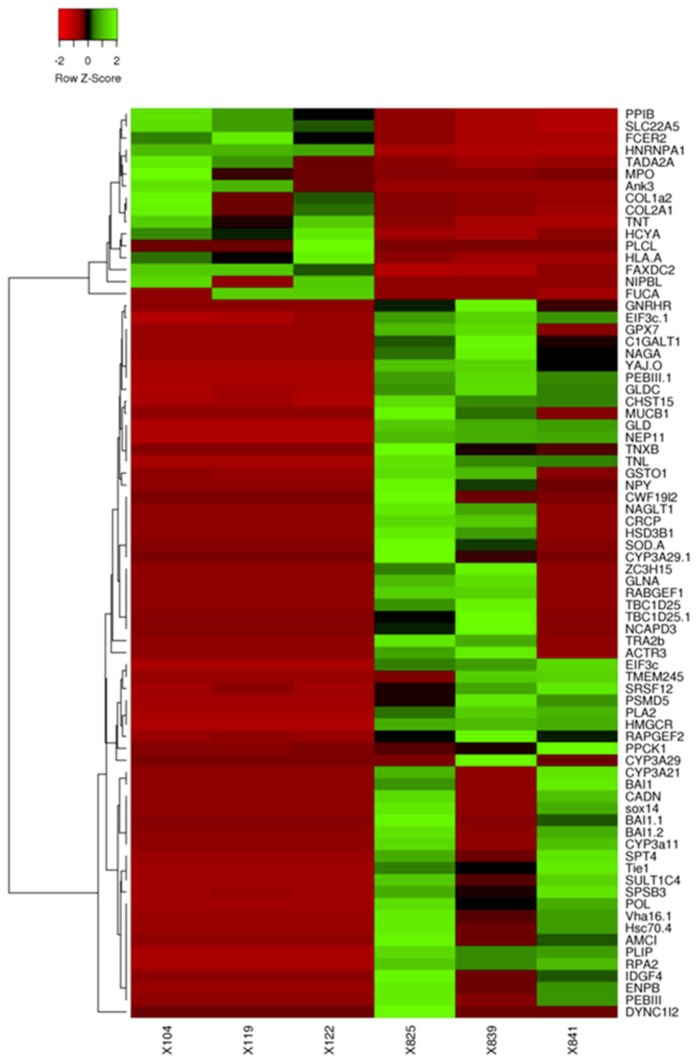
Heatmap of transcripts with an expression change greater than four-fold. The samples X104, X119, and X122 belong to the ganglia of mature abalone, whereas X825, X839, and X841 belong to the immature abalone. The up and downregulated transcripts are indicated by green and red, respectively.

To confirm the expression levels of the identified DEGs between immature and mature Hdh, we calculated the number of clean transcripts for the unigenes in the two groups. A total of 27,978 unigenes (22,566 annotated genes + 5412 novel genes showing a FPKM value >0.3; Table S2) were expressed in the ganglia of all abalone. Using cuffdiff2 [30] and a FDR cut-off of 5%, 256 of the ganglia transcripts (*q*-value <0.05) were differentially expressed between immature and mature abalone (Table S4). Of this set, 42 genes were upregulated in immature abalone and 214 genes were upregulated in mature abalone, and 79 transcripts were confirmed to display significantly different expression levels (log four-fold) between the two reproductive stages (Figure 4).

To identify the putative biological function of these DEGs, GO functional enrichment analysis was performed for the unigene libraries from the ganglia in immature and mature abalone (Table S5), revealing 38 and 74 gene sets that were enriched (FDR < 25%) in immature and mature abalone, respectively. GO analysis of DEGs in the ganglia of mature abalone showed an enrichment in oogenesis (GO:0048477), ovarian follicle cell development (GO:0030707), transmembrane signaling receptor activity (GO:0004888), termination of G-protein coupled receptor (GPCR) signaling pathway (GO:0038032), steroid hormone receptor activity (GO:0003707), regulation of GPCR protein signaling pathway (GO:0008277), adenylate cyclase-modulating GPCR signaling (GO:0007188), negative regulation of cell cycle (GO:0045786), and response to hypoxia (GO:0001666).

### 3.3. Transcriptome Analysis Identified Putative Genes Involved in Sexual Maturation

**Figure 5:**
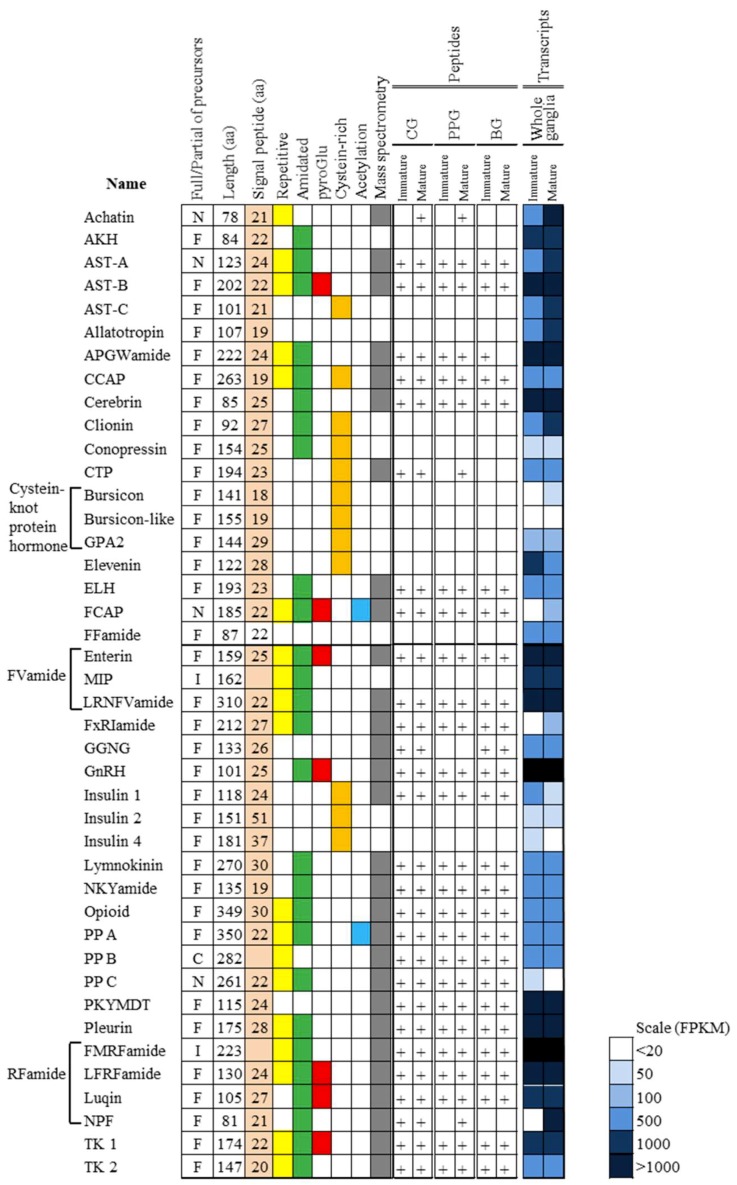
Characteristics of neuropeptide precursors and expression patterns of peptides and transcripts identified from *Haliotis discus hannai*. Shading on signal peptide, repetitive, amidated, pyroGlu, Cysteine-rich and acetylation squares indicates the characteristic of neuropeptide structures. White cells indicate an absence of the neuropeptide or precursor transcripts. “+” represent the neuropeptide was positively detected in the corresponding sample. FPKM in immature and immature ganglia are color-coded as described in legend. Peptide family abbreviations; AKH, Adipokinetic hormone; AST-A, A-type allatostatin or buccalin; AST-B, B-type allatostatin or WWamide; AST-C, C-type allatostatin; CCAP, Crustacean cardioactive peptide; GPA2, Glycoprotein A2; CTP, Calcitonin-like peptide; ELH, Egg laying hormone; FCAP, Feed circuit activating peptide; GnRH, Gonadotropin-hormone; MIP, Mytilus inhibitory peptide or PXFVamide; PP, Pedal peptide; TK, Tachykinin. Deduced protein type abbreviations: F, full-length; I, internal fragment protein; N, amino-terminal partial protein, C, carboxyl-terminal partial protein.

We obtained 18 relevant mRNAs that displayed increased expression in the ganglia of mature abalone when compared to that in the immature abalone (*p* < 0.05; Table 2), which encode neuropeptides and members of the GPCR signaling pathway, along with genes that are involved in oxidoreductase activity, steroid/cholesterol/isoprenoid biosynthesis, lipid and carbohydrate metabolic process, and translational initiation. Among these DEGs, *FxRI*, *NPF*, gonadotropin releasing hormone receptor (*GNRHR*), and glycine receptor subunit alpha-2 (*GLRA2*), which contain the complete coding sequences for the respective peptides, are members of neuropeptides, GPCR, and GPCR signaling pathways. To further explore the associations of neuropeptides with the reproduction of abalones, BLAST searches employing known molluscan neuropeptide pre/preprohormone queries identified 42 sequences encoding neuropeptide pre/preprohormones from the transcriptome of abalone ganglia (Figure 5; Table S6). Supplementary File 2 lists information regarding the Hdh neuropeptide precursors, including the predicted prepropeptide sequences, putative signal peptide sequences, cleavage sites, cysteine residues to make disulfide bridges, and putative bioactive neuropeptides.

### 3.4. Peptidomic Analysis-Based Identification and Quantification of Neuropeptides

Nano-ultra high-pressure liquid chromatography-tandem mass spectrometry (UHPLC-MS/MS) was applied to directly elucidate the maturation-related neuropeptides in the ganglia tissues, i.e., the cerebral ganglion (CG), pleuro-pedal ganglion (PPG), and branchial ganglion (BG). With this peptidomic approach, a total of 119 NR peptides that were derived from 29 precursor proteins were identified in the ganglia of immature and mature abalone (Figure 5; Table S6). Using the intensity-based label-free quantitative LC-MS/MS approach, 18 neuropeptide precursors and 17 proteins showed a significantly different abundance between immature and mature abalone at an adjusted *p*-value of <0.05, FC > 2 (Table S7). Table 3 lists the 18 neuropeptide families, including FMRFamide, crustacean cardioactive peptide (CCAP), LFRFamide, and pedal peptide A and B, which were found to be differentially expressed (*p* < 0.05, FC > 2) between immature and mature abalone. These include 10 known neuropeptide families of mollusks and eight uncharacterized peptides, designated maturation-associated peptides (MAP1–8). The MAPs are predicted as neuropeptide precursors, because of the presence of a signal peptide and peptide cleavage sites (Supplementary File 3). In the CG and BG at the mature stage, MAP-1 expression was slightly upregulated, whereas the expression of tachykinin, allatostatin B, and MAP-2, -3, and -4 peptides was marginally downregulated, although significant differences were not observed (Table 3). In the PPG at the mature stage, CCAP, FMRFamide, pedal peptide A and B, and MAP-5 were significantly upregulated, while LFRFamide was downregulated in the ganglion (Table 3; *p* < 0.05). The majority of the neuropeptides were mutually identified in the ganglia of immature and mature abalone. However, the achatin peptide (GFGDKRGFGD; 1054.48 Da), pedal peptide A (PFDSISSGGGMAGFA; 1399.61 Da), and pedal peptide B (SRGDGLSNFY; 1114.50 Da) were only detected in the mature stage, whereas allatostatin B peptide (QWSNFHSWamide, 1089.48 Da) and the uncharacterized MAP-3 peptide (AGIANQVTRILPIQVLSPDDLM(O); 2379.28 Da) were only detected in the immature stage(Table 3, Table S6-1).

### 3.5. Comparison of Neuropeptides at the Transcript and Peptide Levels

**Figure 6:**
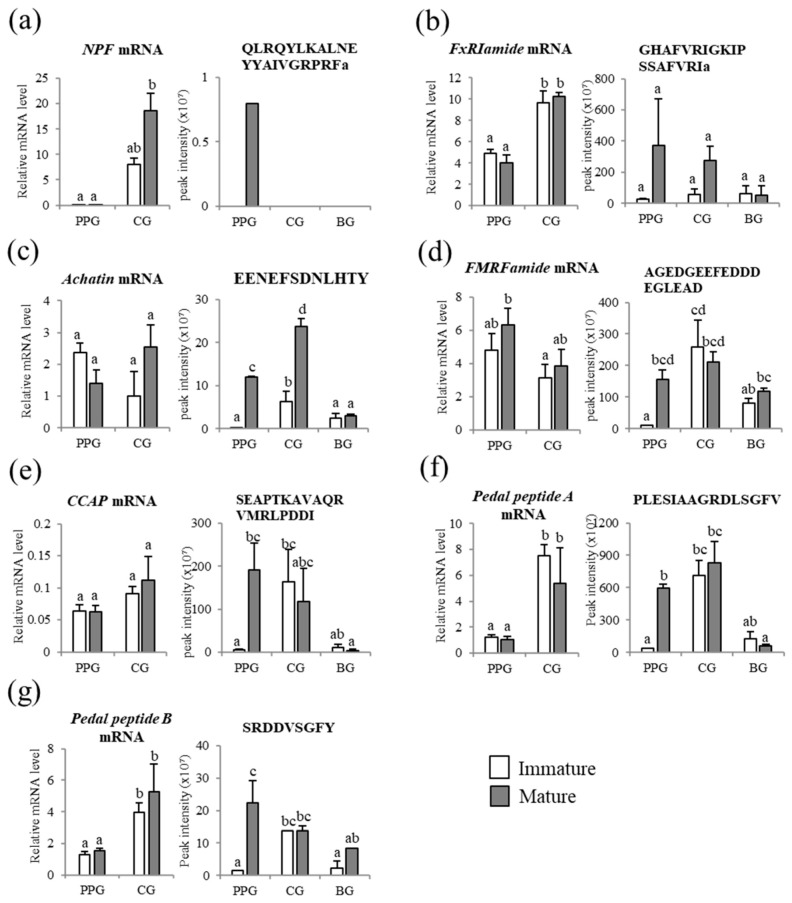
Quantitative comparisons of mRNAs of neuropeptide precursors and neuropeptides in the ganglia PPG, CG, and BG between immature and mature *Haliotis discus hannai*. (**a**) NPF; (**b**) FxRIamide; (**c**) Achatin; (**d**) FMRFamide; (**e**) crustacean cardioactive peptide (CCAP); (**f**) Pedal peptide A; and, (**g**) Pedal peptide B. The histograms on the left are mean mRNA expression levels of neuropeptide precursors determined by qPCR (*n* = 4), and the histograms on the right are mean peak intensity of their representative individual neuropeptides (*n* = 3; Table S7). Data are means ± SEM. Means were compared by two-way ANOVA, followed by Bonferroni’s post-hoc test (SPSS version 23, SPSS). Different lowercase letters (a, b and c) on the bars indicate significant differences (*p* < 0.05).

The neuropeptides were then quantitatively compared at the transcript level to test the expression concordance between the neuropeptide precursor proteins and their encoding genes. We selected representative genes encoding neuropeptide precursors, which were differentially expressed in the ganglia between immature and mature abalone (Table 2 and Table 3). Notably higher levels of *NPF* and *FxRIamide* mRNA in the whole ganglia at the mature stage were detected, matching the similarly high level of NPF and FxRI detected in one MS/MS spectrum of PPG at the mature stage. However, *NPF* and *FxRIamide* mRNA levels, as analyzed by qPCR, had inconsistent tendencies with peptide abundances (Figure 6A,B). The transcript levels of *Achatin* and *FMRFamide* genes showed a tendency of upregulation at the mature stage, corresponding to the high intensities of their peptides in CG and PPG, respectively (Figure 6C,D). Although the mRNA levels of *CCAP* and *pedal peptides A* and *B* did not show marked differences between immature and mature abalone, their peptide levels were significantly higher (*p* < 0.05) in the PPG of mature animals (Figure 6E–G).

## 4. Discussion

With the goal of identifying and quantifying endogenous ganglionic signaling molecules in the abalone related to sexual maturation, we adopted a two-step approach: the construction of a transcriptome dataset to identify DEGs and pathways playing important roles in sexual maturation, followed by a detailed investigation of the expression of specific neuropeptide families using peptidomic analysis. Previous transcriptome analyses have provided insights into the reproduction, growth, and immunity of Hdh [20,21,22]. We established a repertoire of annotated unigenes from the ganglia of immature and mature Hdh to generate a comprehensive reference transcriptome on the neuropeptides in the abalone ganglia. Among the 76,684 unigenes in the CG, BG, and PPG, only 28.5% (21,891) significantly matched with known genes in the reference databases. Thus, 71.5% (54,793) of the unigenes failed in BLAST annotation, which likely reflects the paucity of genomic information that is available on abalone in public databases. These unmatched unigenes might be candidates for novel gene discovery. As is consistent with reports of pooled tissue transcriptomes of Hdh, the BLAST results of the NR database showed that the majority of Hdh unigenes most closely matched with the marine gastropod mollusks *L. gigantea* and *A. californica* [20,22].

Of the 21,891 transcripts that were identified in the Hdh ganglia, 214 and 42 genes were down or upregulated in immature and mature individuals. Several of these genes were identified as neuropeptides and receptors that are involved in GPCR signal transduction processes, such as FxRI, NPF, and GNRHR, as discussed in more detail below. Another group of upregulated genes in the ganglion of the mature Hdh were enriched in the GO terms steroidogenesis, ovarian development, neuro-protection, and metabolism in vertebrates. Although glycine receptors are found in all vertebrates and *A. californica* [42], to the best of our knowledge, the present finding represents the first report on the increased expression level of *GLRA2* in the molluscan ganglia of mature animals. The enzyme 3-Hydroxy-3-methylglutaryl-coenzyme A reductase (HMGCR) catalyzes the rate-limiting step of cholesterol biosynthesis [43] and 3-beta-hydoxysteroid dehydrogenase/Δ5→4-isomerase (HSD3B1), and it is involved in the synthesis of several natural steroid hormones, including progesterone and testosterone [44]. In the swimming crab *Portunus trituberculatus*, the transcript level of *HMGCR* was considerably increased at the mature stage, along with methyl farnesoate, the crustacean homolog of insect juvenile hormone [45]. When considering the localization and steroidogenic activity of HSD3B1 in the lobes of the nervous system of cephalopod mollusks [46], the increases of *HSD3B1* mRNAs in mature abalone suggest that these enzymes are involved in steroidogenic events in the abalone ganglia during maturation processes.

Insect imaginal disc growth factors (IDGFs) are chitinase-like factors that are orthologs of human chitinase-like proteins (CLPs) and they were the first soluble growth factors to be identified from invertebrates [47,48]. In the present study, an IDGF ortholog (IDGF4) that diverged from CLPs was identified for the first time in mollusks, and its mRNA expression level was increased in the ganglia of mature abalone. This is consistent with the malformation of egg shell structures in *IDGF4*-dysregulated *Drosophila* [49], although the function in mollusks has yet to be elucidated. Glutathione S-transferases (GSTs) are enzymes that catalyze the conjugation of the reduced form of glutathione to endogenous and exogenous substances for the purpose of detoxification [50]. Two GSTs in the lophotrochozoan, *Clonorchis sinensis,* showed upregulated expression profiles during sexual maturation and in response to oxidative conditions [51]. In accordance with this result, increased GST transcripts in the ganglia of mature abalone suggest that GST may play a role in the protection of the neural systems under oxidative stress.

Abalone NPF consists of 39 amino acid residues and it shows structural similarity to the vertebrate NPY motif, with a C-terminal Arg-Phe-amide, as previously reported in mollusks [35,52]. The *NPF* transcript abundance in the whole ganglia was higher at the mature stage than at the immature stage that was estimated by the RNA-Seq method, and a partial peptide of NPF, QLRQYLKALNEYYAIVGRPRFamide, was only detected in one spectrum of the PPG at the mature stage, suggesting that NPF plays an important role in the reproductive process of Pacific abalone. Further, in *Sepia officinalis, NPF* expression was significantly upregulated in the egg-laying female [13]. However, in the future, we need to examine the *NPF* transcript in each ganglion, i.e., PPG, CG, and BG, separately, because the expression levels of *NPF* were markedly different between the ganglia when it was examined by qPCR.

FxRIamide is commonly found in lophotrochozoans and it is also called the S-Iamide peptide, owing to its common xSSFxRIamide structure [15,53,54]. The application of synthetic FxRIamide peptides was shown to inhibit the spontaneous contraction/relaxation cycle of the vas deferens in male *L. stagnalis* [54]. The FxRIamide peptide of PPG and CG was abundant in the mature abalone, suggesting that this neuropeptide is associated with the regulation of sexual maturation in abalone. In contrast, the transcript of *FxRIamide* gene showed no significant differential expression profiles in the PPG and CG between immature and mature abalone. In the invertebrate model system, the mRNA expression level of the neuropeptide precursors was not always consistent with protein abundance [55].

Achatin, which is a GFAD tetrapeptide, was first identified from the ganglia of the gastropod *Achatina fulica* [56]. In the supraesophageal mass (SupEM) of egg-laying female, *S. officinalis,* the transcript level of achatin was 100 times higher than that of the male SupEM [13], suggesting that achatin is an important neuropeptide that regulates the female molluscan reproductive system. A higher abundance of the achatin peptide in the PPG and CG of mature female abalone supports this suggestion, although the levels of *Achatin* transcripts in the abalone ganglia were not clearly in agreement with the peptide levels.

At the peptide level, the neuropeptides CCAP, FMRFamide, and pedal peptides A and B showed significantly higher abundances in the PPG of mature abalone. CCAP is typically found in the neurons of mollusks [12,13,15,57] and crustaceans [58,59,60]. In mollusks, CCAP-like peptide sequences show larger variability when compared to a common sequence, PFCNAFTGC, of crustaceans and insects, and CCAP triggered spawning in the ripe Sydney rock oyster, *S. glomerata* [12]. In the present study, we also detected a high expression level of a degraded peptide of CCAP in the PPG of the mature abalone. This finding is in agreement with the high expression of CCAP transcripts in the SupEM of the egg-laying cuttlefish [13] and the presence of the CCAP receptor in the reproductive systems of the cuttlefish *S. officinalis* [61], which implies an important role of CCAP in molluscan reproduction.

FMRFamide has been identified in diverse animal phyla [11,62,63]. In mollusks, FMRFamide is involved in many physiological regulation processes, including cardioexcitatory activity [63], reproductive activity [64,65], and glucose metabolism [66]. We also detected a higher abundance of the FMRFamide peptide in the PPG of mature female abalone, although the mRNA levels were similar between the immature and mature abalone. During the spawning period of *H. asinina*, *FMRFamide* mRNA expression increased from the lowest detectable levels at 36 h pre-spawn to the highest levels at 12 h post-spawn [14]. In *A. californica*, the *FMRFamide* mRNA levels increased from the young to mature stage and then decreased in older animals [67]. In addition, FMRFamide from the subpeduncular area of the brain influences the secretory activity of the optic glands, which control the maturation of the reproductive system in *Octopus vulgaris* [68]. Together, these studies provide a convincing argument that FMRFamide is a reproduction-associated peptide in mollusks.

Pedal peptides were first identified from the pedal ganglia of *A. californica* [69]. In Hdh, three pedal peptide transcripts encoding pedal peptide A, B, and C, and their peptides were detected in the ganglia. Mollusks have several paralogous genes that encode pedal peptides, including four in *A. californica* [70] and three in *L. gigantea* [35]. In the present study, the higher abundance of pedal peptides A and B in the PPG of mature female abalone indicates their close involvement in the sexual maturation process, which is in agreement with the detection of pedal peptides in the central nervous system and hemolymph in *S. officinalis* egg-laying females [13].

By contrast, four neuropeptide precursors, including B-type allatostatin (AST-B or WWamide), CTP, LFRFamide, and tachykinin-1 were significantly less frequent in the PPG of mature abalone. In arthropods, including crustaceans, ASTs inhibit the synthesis of juvenile hormone, which is an important regulator of development and reproduction [71]. Although the counterpart of the juvenile hormone has not yet been discovered, and its biological significance in mollusks is unclear, several AST-B precursors have been characterized in two gastropods, *L. gigantea* and *Deroceras reticulatum* [35,53]. Molluscan AST-Bs are similar to insect AST-B, which is also known as myoinhibiting peptide or prothoracicostatic peptide, sharing a N-terminal tryptophan (W) and C-terminal Wamide [72]. AST-B could suppress the spontaneous contractions of the hindgut and the oviduct of *Locusta migratoria* [73], indicating a function as a reproduction-associated neuropeptide.

The CTP family was initially characterized as an insect calcitonin-like diuretic peptide, DH31, with a primary role in promoting fluid secretion of the Malpighian tubule [74]. In the protostomian lineage, there are two types of CTP: (1) CT-type peptides that are structurally similar to the CT-type peptides in deuterostomes, owing to an N-terminal pair of cysteine residues, and (2) DH31-type peptides that lack the N-terminal cysteines [75]. Starfish *Asterias rubens* CTP caused the dose-dependent relaxation of acetylcholine-induced muscle contraction [40], and CTP expression was also associated with other muscular organ systems, including gonoduct, suggesting that CTP may be involved in the regulation of gametogenesis. In mollusks, the molecular characteristics and physiological roles of CTP neuropeptides are poorly understood, providing opportunities to address not only the importance of the intramolecular disulfide bridge for the functional evolution of the CTP family, but also to determine the biological significance in maturation.

LFRFamide is widely detected in mollusks [13,15,35,40,53]. The localization of LFRFamide transcripts in *L. stagnalis* during parasitization showed expression in the buccal and cerebral ganglia, suggesting that the parasite induces LFRFamide gene expression to suppress host metabolism and reproduction [76]. Thus, the lower occurrence of LFRamide peptides in the PPG of mature abalone indicates an inhibitory function on growth and/or reproduction in abalone.

Finally, tachykinins with a conserved C-terminal pentapeptide, FXGXRamide, have been reported in mollusks [13,15,35,53]. Tachykinins display multiple functions in the nervous system, different kinds of muscles, and especially in the gut tissue [77]. A recent study indicated that the expression level of tachykinin was related to feeding status in the bivalve mollusk *C. gigas* [78], indicating a role in signaling in the energy budget for gonad maturation.

## 5. Conclusions

In this study, we analyzed de novo transcriptome and peptidome of the neural ganglia from immature and mature female Pacific abalone. This information represents the first significant contribution to establish a platform of neural peptide dataset of Pacific abalone and to further understand the pathways that are closely related to the regulation of the reproductive process. Thus, our results highlight the key neuropeptides that are differentially expressed during the sexual maturation of abalone, which are available in regulating the reproductive function and to contribute to the seed production of Pacific abalone.

## Figures and Tables

**Table 1:** Statistics of transcriptome sequencing, assembly and annotation of the *Haliotis discus hannai* ganglia.

**Assembly**	Total no. of raw reads	329,064,126
	Total no. of clean reads	305,530,780 (92.8%)
	Total no. of unigenes	76,684
	Average length of unigene (bp)	741
	>500 bp unigene	32,477
	>1000 bp unigene	15,480
	GC content (%)	45.25
	Proteins with complete ORF	4379
**Annotation**	Blastx and Swiss-Prot Blastx only Swiss-Prot only	21,891 5001 674
	GO	15,585
	KEGG	11,728

**Table 2:** List of significant differentially expressed genes between immature and mature *Haliotis discus hannai.*

Putative Function ID no.	Gene Name	Description	Immature vs. Mature	
			Log2FC	*p*-value
**Neuropeptide signaling pathway**				
TBIU004747	NPF	Pro-neuropeptide F	–8.82	7.73 × 10^−16^
TBIU007725	FxRI	FMRFamide	–2.01	0.001
TBIU006586	Sort1	Sortilin	–2.11	0.013
TBIU017900	Glra2	Glycine receptor subunit alpha-2	–3.29	0.0099
TBIU032133	Pkd1l2	Polycystic kidney disease protein 1-like 2	–9.48	0.054
**G-protein coupled receptor activity**				
TBIU024402	GNRHR	Gonadotropin-releasing hormone receptor	–7.28	3.23 × 10^−5^
**G-protein coupled receptor signaling pathway**				
TBIU011409	RAPGEF2	Rap guanine nucleotide exchange factor 2	–4.91	0.0001
TBIU007260	CELSR2	Cadherin EGF LAG seven-pass G-type receptor 2	–5.09	0.00034
**Oxidoreductase activity**				
TBIU021351	Gsto1	Glutathione S-transferase omega-1	–2.85	4.57 × 10^−5^
**Steroid biosynthesis**				
TBIU020046	HSD3B1	3 beta-hydroxysteroid dehydrogenase/Delta 5-->4-isomerase type 1	–13.8	3.26 × 10^−5^
**Cholesterol/Isoprenoid biosynthesis**				
TBIU019957	hmgcr	3-hydroxy-3-methylglutaryl-coenzyme A reductase	–13.8	2.42 × 10^−10^
**Lipid catabolic process**				
TBIU001813	MtsPLA2	Phospholipase A2	–11.9	5.66 × 10^−5^
**Gluconeogenesis**				
TBIU022277	PPCK1	Phosphoenolpyruvate carboxykinase, cytosolic [GTP]	–3.74	6.87 × 10^−5^
**Carbohydrate metabolic process**				
TBIU014745	Idgf4	Chitinase-like protein Idgf4	–12.9	9.53 × 10^−6^
**Translational initiation**				
TBIU023683	eif3c	Eukaryotic translation initiation factor 3 subunit C	–2.59	1.57 × 10^−5^
**Other pathways**				
TBIU021499	Cyp3a11	Cytochrome P450 3A11	–13.2	0.0001
TBIU019312	Tie1	Tyrosine-protein kinase receptor Tie-1	–12.9	2.91 × 10^−6^
TBIU016501	Bai1	Brain-specific angiogenesis inhibitor 1	–13.2	0.00016

**Table 3:** List of neuropeptides up and downregulated in the ganglia of *Haliotis discus hannai,* as determined by mass spectrometry at immature and mature stages.

Neuropeptide Family	Log FC^a^ (Mature vs Immature)	*p*-value	Peptide Sequence and PTM^b^	Mass (Da)	Mature^c^	Immature^c^	*p*-value^f^
Branchial ganglion (BG)							
MAP-1	4.15	7.09 × 10^−3^	SVILTSILLQERRYDRMS	2179.18	0.92 ± 0.18	0.42 ± 0.04	n.s.
Tachykinin 1	−3.06	4.75 × 10^−2^	EALDDNTAASLYKLLQPAYQSTVAE^d^	2710.33	ND	20.60 ± 4.89	N/A
			pQPHFGFHGVRamide	1162.58	3.29	4.57 ± 0.75	n.s.
			TELGFGYVGSRamide	1183.60	10.30 ± 2.91	17.64 ± 1.72	n.s.
MAP-2	−1.35	4.61 × 10^−2^	GGGFGPASNPDSWSEVYRTGN	2153.94	0.26 ± 0.00	0.21 ± 0.01	n.s.
MAP-3	−2.60	4.42 × 10^−2^	AGIANQVTRILPIQVLSPDDLM(O)^d^	2379.28	ND	0.21 ± 0.14	N/A
Cerebral ganglion (CG)							
Allatostatin B (=WWamide)	−2.74	6.01 × 10^−4^	QWSNFHSWamide^d^	1089.48	ND	0.10	N/A
			AGWDNGFASWamide	1108.47	5.78 ± 1.42	4.11 ± 0.47	n.s.
			NWNQFITWamide	1106.53	4.14 ± 0.56	2.56 ± 1.29	n.s.
			WGNFGTSGKKWASSDFPAWamide	2127.00	7.21 ± 2.93	8.47 ± 0.07	n.s.
MAP-4	−1.69	4.18 × 10^−2^	YVHFNIGNDHQVS	1528.71	0.52 ± 0.15	0.80 ± 0.15	n.s
Pleuro-pedal ganglion (PPG)							
Achatin	2.41	3.83 × 10^−2^	EENEFSDNLHTY	1496.61	1.19 ± 0.02	0.02	n.s.
			GFGDKRGFGD^e^	1054.48	0.08	ND	N/A
FMRFamide	1.57	3.09 × 10^−2^	AGEDGEEFEDDDEGLEAD	1940.69	15.59 ± 1.68	1.41 ± 0.27	*
			DGEDEKRFMRFamide	1427.66	0.47 ± 0.15	0.42 ± 0.01	n.s
			DGQDKRFMRFamide^e^	1297.64	0.06 ± 0.01	ND	N/A
			FMRFGKSGEEE	1315.59	0.90 ± 0.21	0.29 ± 0.03	*
			SGDDEKRFMRFamide	1385.65	0.17 ± 0.03	0.12 ± 0.10	n.s.
CCAP	2.94	1.57 × 10^−3^	SEAPTKAVAQRVMRLPDDI	2096.11	19.14 ± 3.59	0.56 ± 0.16	*
Pedal peptide A (PP-A)	1.29	1.22 × 10^−4^	SFDSINKGSGLSGFM	1545.71	13.82 ± 1.09	1.63 ± 0.30	*
			PFDSISSGGGMAGFA^e^	1399.61	4.75 ± 0.56	ND	N/A
			PFDSIASGRGIAGFA	1464.74	24.22 ± 11.65	2.74 ± 0.04	n.s.
			PLESIAAGRDLSGFV	1530.80	59.38 ± 3.57	3.69 ± 0.52	*
			SFDSINAGSGLSGFA	1428.65	5.67 ± 1.63	0.95 ± 0.12	n.s.
			PFDSISGSSAFSDFA	1533.66	3.53 ± 0.25	0.69 ± 0.25	*
			PFDSIASGGGMAGFA	1383.61	4.31 ± 0.65	0.29 ± 0.07	*
			PFDSIASGRGIAGFA	1464.74	24.22 ± 11.65	2.74 ± 0.04	n.s.
Pedal peptide B (PP-B)	1.67	4.50 × 10^−4^	SPENDLSSFY	1044.45	2.24 ± 0.68	0.17	n.s.
			SNEDLSGFY	1030.42	6.24 ± 1.44	0.17	n.s.
			SRGDGLSNFY^e^	1114.50	2.97 ± 0.74	ND	N/A
MAP-5	9.73	1.29 × 10^−5^	RSLSLDDTYWVDNVD	1796.82	76.19 ± 13.23	4.84 ± 2.49	*
MAP-6	2.61	1.73 × 10^−2^	MGIGKGAQSFGDGQNNDQ	1822.79	0.61 ± 0.21	0.28 ± 0.02	n.s.
MAP-7	2.83	7.90 × 10^−3^	STIFDRMGRFYamide	1390.68	3.69 ± 1.31	1.57 ± 0.41	n.s.
MAP-8	3.05	8.73 × 10^−3^	TETRAAFWDNAAGRQPY	1952.91	1.12 ± 0.55	0.04 ± 0.02	n.s.
CTP	−1.189	1.93 × 10^−2^	AVLANTQRIIKAE	1425.83	1.95 ± 0.46	1.53 ± 0.25	n.s.
LFRFamide	−1.72	2.90 × 10^−4^	AKEEQDSEAAVVAPSEHH	1989.90	21.92 ± 1.66	22.93 ± 4.51	n.s.
			NFHWGRETEE	1303.56	29.83 ± 6.31	47.67 ± 3.33	*
Tachykinin 1	−1.63	2.16 × 10^−4^	EALDDNTAASLYKLLQPAYQSTVAE	2710.33	8.13 ± 2.44	14.17 ± 4.98	n.s.
			pQPHFGFHGVRamide	1162.58	5.40 ± 0.78	6.68 ± 1.60	n.s.
Sensorin-A	−1.57	1.05 × 10^−3^	RSLDQSERRLVA	1428.78	34.12 ± 6.33	31.79 ± 3.58	n.s.

^a^Peak intensity values were normalized to the mean intensity of all peaks within a sample and then to the mean of the individual peptide ions across the samples (Table S7-2), ^b^Post transcriptional modification ^c^Peptide peak intensity with means (divided by 100,000,000) ± SEM, ^d^Neuropeptides identified in the ganglia at immature stage only, ^e^Neuropeptides identified in the ganglia at mature stage only, ^f^Neuropeptides with differential abundance are indicated with asterisks (*) (*p* < 0.05) using Mann-Whitney U test. n.s., not significant; ND, not detected; N/A, not applicable.

## Supplementary Materials

The following are available online at https://www.mdpi.com/2073-4425/10/4/268/s1, Figure S1: Example of peptide identification and quantification by MaxQuant: SQVTEDDLLQTVSR spectrum, Table S1: Information on the primer sets used in this study, Table S2: Summary of the transcriptome annotation and stage-specific expression data of abalone ganglia transcriptome, Table S3: Functional annotation of unigenes based on KEGG categorization, Table S4: List of DEGs obtained from mature and immature groups, Table S5: List of enriched gene sets from mature and immature groups, Table S6-1: Summary of neuropeptides identified in Pacific abalone ganglia, CG, PPG, and BG, at immature and mature stages, Table S6-2: Peptides identified in the BG at mature stage, Table S6-3: Peptides identified in the BG at immature stage, Table S6-4: Peptides identified in the CG at mature stage, Table S6-5: Peptides identified in the CG at immature stage, Table S6-6: Peptides identified in the PPG at mature stage, Table S6-7: Peptides identified in the PPG at immature stage, Table S7-1: Quantitative comparison of proteins identified in Pacific abalone ganglia, CG, PPG, and BG, at immature and mature stages, Table S7-2: Quantitative comparison of neuropeptide precursors identified in Pacific abalone ganglia, CG, PPG, and BG, at immature and mature stages, Table S7-3: Results of quantitative analysis of identified neuropeptide in Pacific abalone ganglia, CG, PPG, and BG, at immature and mature stages. Supplementary File 1: Peptide sequences for blast queries. Supplementary File 2: Putative precursors for Pacific abalone neuropeptides deduced from transcriptome analysis. Supplementary File 3: Sequences of the precursors of novel maturation-associated peptides (MAPs).
