# Supplementary material for: Neural Ganglia Transcriptome and Peptidome Associated with Sexual Maturation in Female Pacific Abalone (Haliotis discus hannai)
Source: Genes (Basel). 2019 Apr 2;10(4):268. doi: 10.3390/genes10040268 (PMC6523705; doi:10.3390/genes10040268)
Supplement: Supplementary file 1 [file genes-10-00268-s001.zip › Supplementary Fig. S1.pptx]

## Slide 1
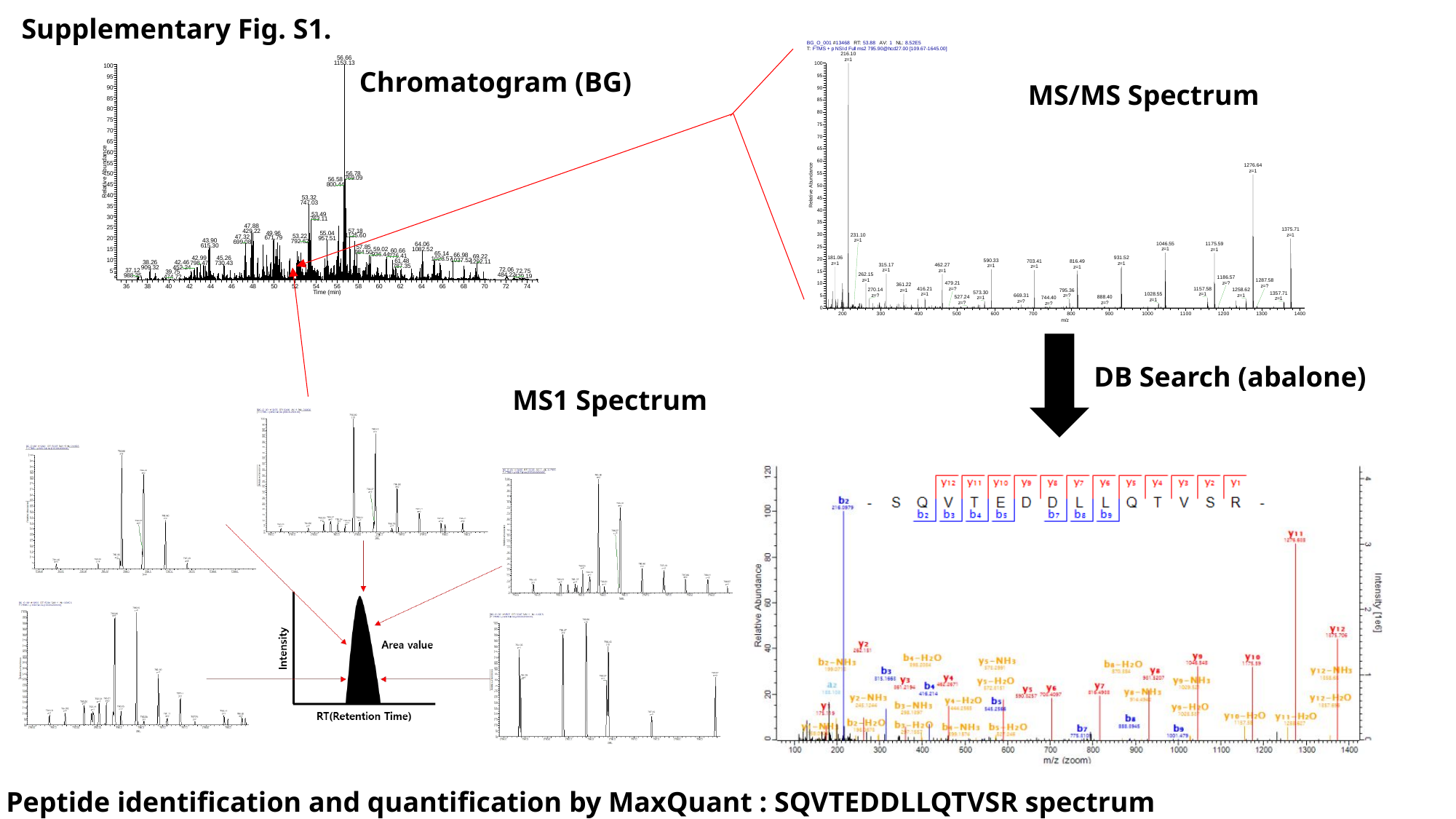

Supplementary Fig. S1.
56.66
1153.13
100
35
30
25
20
15
10
5
36
38
40
42
44
46
48
50
52
54
56
58
60
62
64
66
68
70
72
74
Time (min)
95
90
85
80
75
70
65
60
55
Relative Abundance
56.78
50
769.09
56.58
45
800.44
40
53.32
747.03
53.49
763.11
47.88
429.22
57.18
49.96
55.04
1125.60
53.22
47.32
671.79
957.51
43.90
792.62
699.08
64.06
615.30
57.85
1082.52
59.02
60.66
984.50
65.14
936.44
66.98
776.41
69.22
42.99
45.26
1028.57
1037.52
61.48
1292.11
42.46
38.26
798.47
730.43
787.35
452.24
909.32
72.06
37.12
72.75
39.75
484.22
988.35
429.19
474.72
Chromatogram (BG)
MS/MS Spectrum
DB Search (abalone)
MS1 Spectrum
Peptide identification and quantification by MaxQuant : SQVTEDDLLQTVSR spectrum

## Slide 2
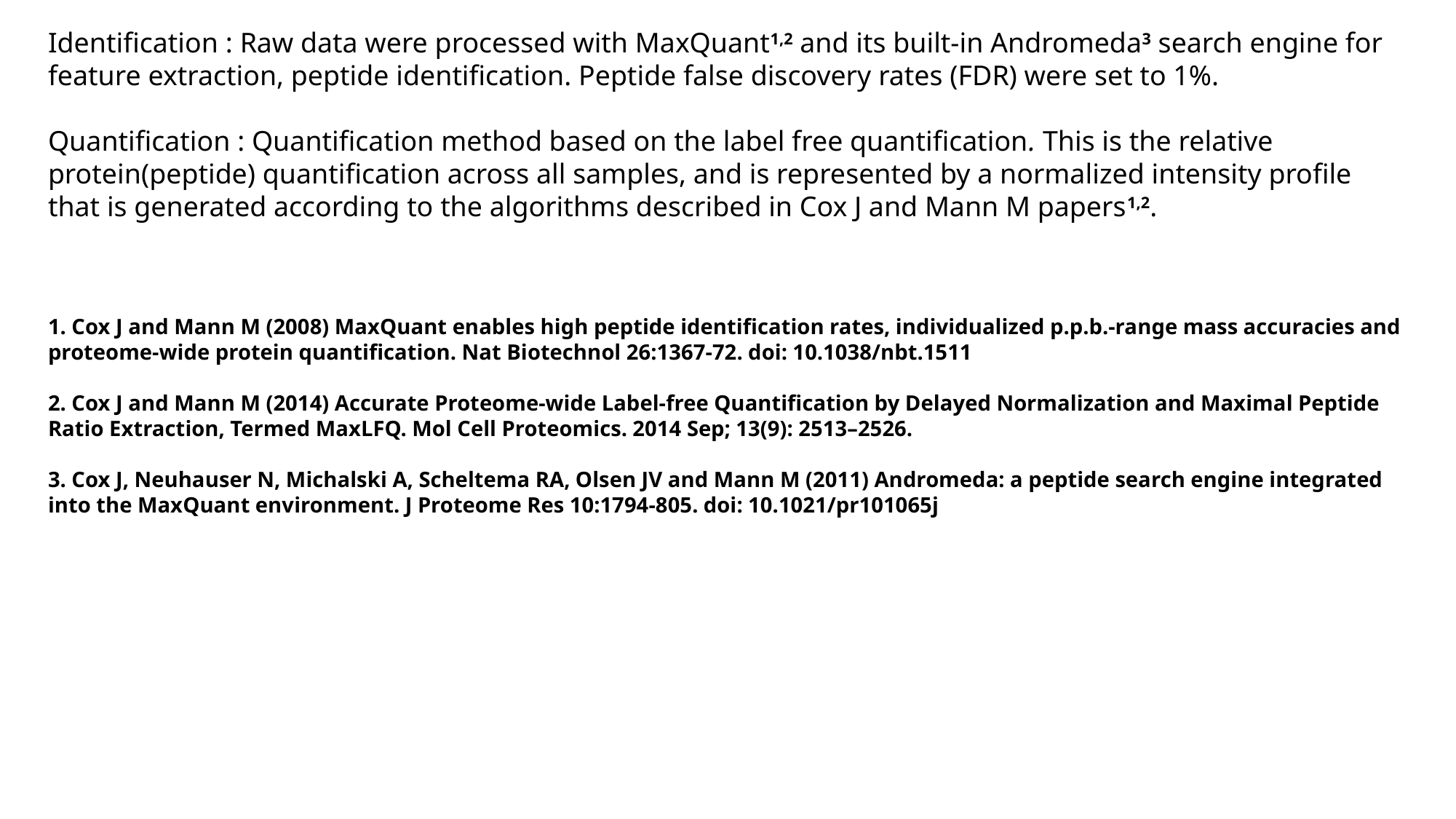

Identification : Raw data were processed with MaxQuant1,2 and its built-in Andromeda3 search engine for feature extraction, peptide identification. Peptide false discovery rates (FDR) were set to 1%.
Quantification : Quantification method based on the label free quantification. This is the relative protein(peptide) quantification across all samples, and is represented by a normalized intensity profile that is generated according to the algorithms described in Cox J and Mann M papers1,2.
1. Cox J and Mann M (2008) MaxQuant enables high peptide identification rates, individualized p.p.b.-range mass accuracies and proteome-wide protein quantification. Nat Biotechnol 26:1367-72. doi: 10.1038/nbt.1511
2. Cox J and Mann M (2014) Accurate Proteome-wide Label-free Quantification by Delayed Normalization and Maximal Peptide Ratio Extraction, Termed MaxLFQ. Mol Cell Proteomics. 2014 Sep; 13(9): 2513–2526.
3. Cox J, Neuhauser N, Michalski A, Scheltema RA, Olsen JV and Mann M (2011) Andromeda: a peptide search engine integrated into the MaxQuant environment. J Proteome Res 10:1794-805. doi: 10.1021/pr101065j
