# Supplementary material for: Neural Ganglia Transcriptome and Peptidome Associated with Sexual Maturation in Female Pacific Abalone (Haliotis discus hannai)
Source: Genes (Basel). 2019 Apr 2;10(4):268. doi: 10.3390/genes10040268 (PMC6523705; doi:10.3390/genes10040268)
Supplement: Supplementary file 1 [file genes-10-00268-s001.zip › Supplementary File 1.docx]

>APGW_pfu

MEVTKFLLLCTAIAQHLILVSSDGELIDKRRPGWGKRNVDNLVDDEDDYVEAEKRKPGWGKRSDIDSQMDKRKPGWGKRDFSEIGEIDKRKPGWGKRSMDDLNNIIKRRPGWGKRSGDLYDALQMYKRRPGWGKRSFPESLILDKRRPGWGKRSFVDIFDTAEDNDTSDINKRRPGWGKRSDIFSVERRRPGWGKRAPGWGKRSDLVDNCELIDLEVERLLQEIQKIEEYRLQQCGTPSEDGITVPAYN

>APGWa_C.gigas

MESPSLYLIVIALVLGIVSTDEELIDKRRPGWGKRGSLDFGSNNLPNSIIEKRKPGWGKRGMEDEFEMNKRKPGWGKRAPGWGKRSFNEDLNNFIEKRRPGWGKRTSDLMDALNAMEVNKRKPGWGKRSEMEKRKPGWGKRTVPELSETDLSYSSIPTDSFSVLDKRRPGWGKSWRNITDLTAEDNHLRHQILATQSGKNKGVIHYWNSGSFRQSSESRLSPKGIGYTKNDEPDRFYTTTSVNDPACDMQGFNLVILPDPRKSSTEIFMSPRVWVLTLGQGQMDV

>APGWa_M.edulis

METLNIFLVIFSLLGTIIIASSSDESSERKKRDLDTIDDTNNDFLTADKRRPGWGKRSFDDDILNNLDKRRPGWGKRSDMLFDSEEIEKRRPGWGKRSSSLYDDEKRKPGWGKRSSALLDDLSLYNSIVKRRPGWGKRSDTFKVDIRRPGWGKRTPGWGKRSGPNMCMDFQDEILQLYKLLNEAEKLHSECEALNI

>APGWa_A.californica

MLLAKISVVVLLLAIDGTSSSESTDNVVLSSSPDSQKAATSRHKRAPGWGKRSSLNDEDLFADSDSAQELLDSVAALKRAPGWGKRFSGLMSEGSSLEAKRAPGWGKRGQEIDVDEDGSEQEKRAPGWGKRAPGWGKRAPGWGKRAPGWGKRAPGWGKRAPGWGKRSGGDYCETLEKMVDAYIYKAVEVDSRRLADCGSGEGTNEPFRK

>APGWa_T.diomedea

MRLTKFSQLSVMLAQIVLLISIAVIDLTQSSDSAAAFNSAKKVTSSENSLSPNRHKRRVTDEALFGGDGDTQQAVEALKRAPGWGKRFFDGMAEAEKRDPGWGKRSSDFDDDKRAPGWGKRAPGWGKRSPGWGKRGWGKRAPGWGKRAPGWGKRSDDSDLCQRLDTIADSFLMEARKVNAIFLKECGSLETGNDPFRK

>APGWa_L.gigantea

MYFSTHLAALFFSLFVLLIQCATSEELSSSLLEAEEEKRAPGWGKRNILSSSEKDFLESKRAPGWGKRSLSALLDDADDWSDDVSQDYSKRGWGKRSLESFAADKRAPGWGKRAPGWGKRAPGWGKRAPGWGKRAPGWGKRAPGWGKRAPGWGKRSSDECSTVENEFNYYISKAVQTQIKLLTNCFESGEKINSPLA

>APGWa_H.asinina

MSSFINSLTLGVVVVLSVLSCSLGEDQVASVTGDKEKSDFSQRTLDSVHVLKKRAPGWGKRSLDEDVDSDDDMSYDSESPEDILSGADLSKRAPGWGKRTLDILEDYTKRAPGWGKRDSLDVKRAPGWGKRDIDMDKRAPGWGKRAPGWGKRAPGWGKRAPGWGKRAPGWGKRAPGWGKRSDTSCAGIDEEVDYYIYRAVQAEARRILECGSKYNGNDVLRK

>Achatin_pfu

TSLNCFVVLMMTLPLVSLGISDSRLQGSETDGADSGQYPYSLENGIQDKRGFWDKRGFGDKRDISNEKLALLQEMMPQRRVSFGFIHYPRIPLRQRHFFRSNPKLVALLRKEVGKRGFWDKRGFGDKRSDNTDSNNFHLYSDI

>Achatin_C.gigas

MAQWLEHQALEVLNMRTPLLILLISLAFTFGTAENHLLEQYENDEDIQHQLQNEAYYKRGFWDKRGFWDKRMSHKDFSHGSDDSSWNNLILLLKDYIRYKHESNKKEQVFSNGLQNSH

>Achatin_a.fulica

MASSPHYLLLVVVLVVTLTHITASDFEFLEPDQDVEPFEEDVFLLKRGFADKRGFADKRGFADKRGFADKRGFADKRGFADKRGFADKRGFGDKRDEISPALLRLLQHSYQSQPLPLRGHSAFSRLLARQGLWE

>Achatin_H.lucorum

MASVSHLLLLAFTLATAFNLIASHELDYLEDADLDSDEEDFLGKRGFADKRGFADKRGFADKRGFADKRGFADKRGFADKRGFADKRERISPLVLRLLQRSYQNQHCH

>Achatin_L.valentiana

MASLSLYFLATVAMAVALNLIAASDIEYSESDSDLGSFEEDEYISKRGFADKRGFADKRGFADKRGFADKRGFADKRGFADKRGFADKRGFADKRENINPLVVKLLQGGFLGKNDQGTGHSSFSRLLARQGFLE

>Achatin_L.stagnalis

MTSLSHYALLTFVVLVALDYLAANELEFLEAEAEGEPFEESDLILSDFDDKRGFADKRGFPDKRGFADKRGFADKRGFADKRGFADKRGFPDKRGFADKRGFADKRGFWDKREQMNPLLVKLLRHSQHQPLPYGQNSFSRLLAQQGFFE

>Achatin_L.gigantea

MLPQNILIWLSVLVASVGITVFADELEFTDAIEAVDGPRLIVAEGNEILDKEDATKRLFGDKRLFGDKRIFLENPEHKRLFLDKRGFGDKRGYGDKRGYGDKRGFGDKRGFGDKRGFADKRGFGDKRSSASSLRPFLLAMLDNFGSRRPKNSYAYWSHRPGLLIRNGRSA

>Achatin_T.diomedea

MLPQNILIWLSVLVASVGITVFADELEFTDAIEAVDGPRLIVAEGNEILDKEDATKRLFGDKRLFGDKRIFLENPEHKRLFLDKRGFGDKRGYGDKRGYGDKRGFGDKRGFGDKRGFADKRGFGDKRSSASSLRPFLLAMLDNFGSRRPKNSYAYWSHRPGLLIRNGRSA

>Achatin_A.californica

MTSSAQCLLLTMLLVATLDFLYAEDIGFAETDSELTPFAGAVADHVLKEGFGKRGYFDKRGFFDKRGDASKRGFFDKRGFFDKKSYADSKDEESNAALSDFAEDKRGFFGKRYYGSKREEISPRVLNLLLRNYQIHPSPLNGHNAFSRLLAKQGFWEN

>Achatin-like_C.gigas

MAQWLEHQALEVLNMRTPLLILLISLAFTFGTAENHLLEQYENDEDIQHQLQNEAYYKRGFWDKRGFWDKRMSHKDFSHGSDDSSWNNLILLLKDYIRYKHESNKKEQVFSNGLQNSH

>AllatostatinB_pfu

MKCLIHCAIVITTLTFSLCEIYDEHKEADELDNISPNLQKDLHLKSSLSDSRTADKRAWQTSSWGKRKWPGTNVWGKRKWMNVWGKRKWASMGTWGKRSDPLIDNGLENIHKRDTEHSFPLKRKWNQFVTWGKRSMPIQRKWASVSLWGKRSQDNYNEPEKRKWSALTSWGKRDDVGDINSDKNKRKWAMSAWGKRTNPIDGDNDMNKKWALASWGKRNDIYNTPSLQDNDAKSDNTVDKRKWSNFSSWGKRMSIQNWRKRPWLWAKRRRGWSAMSSWGKRSSLDTKENDDRQLLSV

>AllatostatinB_C.gigas

MLCHLEQLLLSCIVLCLVKVCASLKAQDEASVNDHDIVRRQAMGHSFGDELEGFDLDPKRVNNWNQFPAWGKRLSKRRWSSLGAWGKRSWLDRLISANNNWGKRWKSMSNSWGKRQAPSEFDGLSDDYINIKKRSVDSKFSHNSRNKRSIPTELSPEQNEEKRRWSSLSAWGKRSDDDEKRRWSSLSAWGKRSNPEAIDDNDSDNISKRKWSSFSSWGKRGDPVDLSKRLYSYWQNRLMTNNPWMERRGWNAFSSWGKRSMD

>AllatostatinB_A.californica

MTLHLASPFILLFTIAYSLTSAVQGLEPLPAASLSDSPASGADVPPLPSSAATNAAVDKEWLRQKLEEGQFLPQQDKRWGGINSWMTHRLGGPSERDSSQDSLDKQLLVNNVQNYDDSSKRKWSKFSSWGKRDASEETPEGGEGEDGLGAVKKWKNMAVWGKRAEDGLDKRWKQMATWGKREDGDVLGLGTDKRWKQMASWGKRLDDSDRDKKWKQMSVWGKREDNGEPLDKKWKEMSVWGKRDTLDDPEKRWKQMAVWGKRQGLDDRNDKRWKQMATWGKRNSSENYDKRWKQMSVWGKRDGDGDLDKRWKQMSVWGKRDGDGDLDKRWKQMSVWGKRNGDGDLDKRWKQMSVWGKRDGDEDVEKRWKQMSVWGKRDGDADLDKRWKQMSVWGKRDEDGNLDKRWKQMSVWGKRDEDGNLDKRWKQMSVWGKRDGDDNLDKRWKQMSVWEREREREWV

>AllatostatinB_L.gigantea

MDLKTILCLIIYSLLLQISHAEEQLANDIELSNSLNPVDKRAWKSSYLNTWGKRWNPRYNLRGYQRMPIWAKRWTNSGLITWGKRSADTEIPIHKRKWNQFITWGKRSGVPSIVKRSVGDELVPWGKNKDTLPELNTSSDNLDNKLIDLETTPSTDKLSLDEKRASDKGWNGFTTWGKRANKDWSSLSTWGKRGQNKDWSSLTTWGKRGHDRDWNSLTTWGKRANKDWSSLSTWGKRARENDWSALSTWGKRANNKDWASLTTWGKRANDRDWNSLTTWGKRAKGNNWSGLTTWGKRANKDWNSLTTWGKRANKDWNSLTTWGKRGNKDWSGLTTWGKRGNKDWSGLTTWGKRGNKDWSGLTTWGKRGNKDWSGLTTWGKRGNKDWSGLTTWGKRGNKDWSGLTTWGKRGNKDWSGLTTWGKRGNKDWSGLTTWGKRSPDATSEDSGELSTLDKKDIKGWNGLTTWGKRFAGDKNKWSSLTTWGKRDDNNKQDDKKWAQLSTWGKRSPEDAAELWKIYDSNGDGIMDKEEMVSFLRSAASQKDSQQDEKS

>AllatostatinC_Scaffold473623.

EDKFDTGSRLRVLTEALKEAYKRELNYYNLQEARIINQISALESQKKSIQARKRSHVQCLVNVIACYRKRK

>AllatostatinC_C.gigas

MELTQSVFVLKLYAAVVAVLLVAEVHAQPQKFSTEIQQTGDESSTDLNLFKMALREAYNRELEFYEQQEAQIVKQLAALENDRNQIRERKRSHIRCLVNVIACYRKK

>AllatostatinC_C.rhizophorae

MEISHSVFVLKIYALVLAVLLATEVHAQPQKFNTEIQQTSDDSSAELNLFKMALREAYNRELEFYETQEAQIMKQIAALENDRSHLRERKRSHIQCLVNVIACYRKK

>AllatostatinC_M.galloprovincialis

MNFSKSLSIIVKFLTVLLVLVLSTHAQMDVDEKALSTQAVEDYRFMKSILEVFYKSQLEQFQEQEAFIQQQLDILHDQREAVRTKKRSHVQCLVNVVACYKKRR

>AllatostatinC_P.dumerilii

MVDFTAHTLAIVLIINCICIAFAEPAPSELGSVGDYENEHYKSLQGGMLERRLREDIEREMTDVKKLEHQLMGHLNLIQEKKRQLEIKKRQPVQCLVNIVSCWKRK

>AllatostatinC_H.diversicolor

MRTAVFLLFLVAFVVLCQMVSCMSESEKAALSGQGSSSDTVLLKRSLRQAYEDQLAMLQEAEEDLTQQIAALQQQRQQITQRKRSHYMCLVNLITCYRKRK

>AllatostatinC_L.gigantea

VLTLTLFCICLNEVSSNKILPGEEHRQLVQITEESSNNDRLLKRSTLQDAYESHLAMLMEAEQSLTKQIEELKLRREELSNRKRSHVMCLVNLISCFRKR

>Allatotropin_pfu

MVPRTMQILKLMLVFFLTVLIVDALPSKSLSHGRSKRGFRQSIIDRMGHGFGKRADSSLYDTYLDNEPQHGFMTVEEFAGNIMQRSDLAEAVVEKFIDSNKDGIISAEELLSKPSE

>Allatotropin_C.gigas

MERARITRYIMQMVKVVLVTVLLTLCVIVDCLPHSSTKMRQKRGFRQSIVDRMGHGFGKRANTDLYDPYLTKTPNLMTADELTTNILNSEDLAQAIVKKFIDLDEWARIFFVHTLKYGVPIGHFDLRYYYRSITFATPHPRLRNFALLPSTFYLLNSVVFCAQLDWYTSLLEKKRVPWVLGILPHCQKCHRALRWTNQMETFVTTVRGQIKIPKRQMSAHAKPVLQKPPPIKFKSAPPPEAKSNELSPLLKVVLGMFPLTAYGLAYWQWQRRKWKINLLKTIDERTAADPVPLTDLLDTADMNDLDYTKRNPLDVNPTQDPKNPRLFSNRDINAIASYLGTDPVFVDSDLESSVEGGPIGGQTNIKFNNRHMEYVLTWLSLAIGMSVLWRNQAFPRALTKRYTRTEIMIGFGKITGNDALGTK

>Allatotropin-like_A.californica

MLSAPSIAHTGVALLVLMCLCPFSQSTEASLSRAKRGFRLNSASRVAHGYGKRGYASSSGAVPYPELARDVLDNLRAEEEEKELEWSIMSVDELASLLQSHPKLARALVKKFVDINGDNLVTAEELFRPPTRK

>Allatotropin_M.californianus

MRFCGLLALFSLSIVIVDSSPALSRHKRGFLAGVHDRMGHGFGKRTDPILSNYVDDIDKDDLMTVEDLVRHIMQSEVLADAIVRKFIDINDDGSVSYQELLRKLVR

>Allatotropin_H.asinina

MMRTTLLMLGLLLVAVVASLPHNTHSRQKREFRANSADRVAHGYGKRTYSSWENSFNTDSKHLLSVEDLANLVANNPTLAEALIRKFVDVDDDGFVGNNELFERSNE

>Allatotropin_L.gigantea

MKLTLVLVTVTLTITILANAYPQSPSLSHHRSKRGFKANSASRVAHGYGKRGFPSWKNYFQDGGSDVLSVEDEMAELVAENPSLAKALLRKFVDTDNDGIISTTELLGTKQMLK

>Buccalin_Pfu

MDPYMFRGYLGKRMDSRMFSGQLGKRALDRKMFISQLGKRLDNRMFFGRLGKRMDYRMFSGQLGKRGLDNHMFVGHLGKRLIPVRNSAYYYRYGSRGRPIYTQTRGMDRFSFAARLGKRDAQNGTLTFSRQVFFP

>Buccalin_C.gigas

MWSTNYATTVFGFFCFVQVFVLTVSQHISSHNDDYTKHLENIKFLNKEAEISPKQPADDDVDFGNSDTADDLSDLTEEEKRALDRYSFSGSLGKRGLDRYSFYGGLGKRALDRYGFFGGLGKRALDQYGFAGSLGKRALDRYSFMGGLGKRKLDQYGFAGRLGKRALDRYGFVGTLGKRKLDQYSFMGNLGKRRLDSHRYFGSLGKRALDRYGFFGGLGKRADTLGNSQENIQGADKDEKFEQKRLYPYWYYRQGGSPIYTQTRGIDRFSFAARLGRR

>Buccalin_C.rhizophorae

MRSYNYAKTVFGCFCLIQLFLCAVSQHISSHNDDLTKHLESIRFLNRQTESSPKHTDDDTDDFRNSDISEDLSDLTDEEKRALDRYSFYGGLGKRGLDRYNFYGGLGKRALDKYGFFGSLGKRALDRNIFSGSLGKRALDRYSFMGGIGKRKLDRYGFAGRLGKRELDRYGFTGSLGKRRLDQFGFMGGLGKRRLDSHRFIGGLGER

>Buccalin_L.gigantea

MAARKHELVLVLTSVLCFVSSIVGDPNVPSDSQDNSALTQDDFAKRGMDKFGFAGGVGKRGLDKFGFTGQLGKRDMDSFGFAGQLGKRGLDQYGFTGQLGKRGLDQYGFTGQLGKRGLDQYGFTGQLGKRGLDQYGFTGQLGKRGLDQYGFTGQLGKRGLDQYGFTGQLGKRGLDQYGFAGQLGKRGLDQYGFTGQLGKRGLDQYGFAGQLGKRGLDHYGFAGQLGKRGLDQYGFAGQLGKRGFDQFGFAGQLGKRGLDHYGFAGQLGKRGLDQLGFTGQLGKRQMDIFGYRGQLGKRQSIDKYSFLGAGIGKRSVKNTAGIKKDDA

>Buccalin_L.stagnalis

MDRYGFFGGIGKRRLDRFGFYGGIGKRDAGGFDDWNGATEDLEKRQLDPFGFSGGIGKRKMDRFSFTGGIGKRRMDRYGFSGGIGKRRLDRFSFTGGIGKRRMDPFSFTGGIGKRRLDRFGFAGGIGKRGLDKFGFAGGIGKRRLDRFNFAGGIGKRTPDDFEDNVVDFEKQADEVRELDKRSTDTAESHMVA

>Buccalin_A.californica

MAHHRGHRHILLYVSLALSLGLALAEDATDPSDDTGSFDDVEAVSEEADLDPYSMSQELNKRPNVDPYSYLPSVGKRAFDHYGFTGGLGKRKIDHFGFVGGLGKRQIDPLGFSGGIGKRYDSFAYSAGLGKRGMDSLAFSGGLGKRGMDSLAFSGGLGKRGMDSLAFSGGLGKRGMDSLAFSGGLGKRGMDSLAFSGGLGKRGMDSLAFSGGLGKRGMDSFTFAPGLGKRGMDSLAFAGGLGKRMDGFAFAPGLGKRMDSFAFAPGLGKRGMDSLAFAGGLGKRMDSFAFAPGLGKRMDSFAFAPGLGKRGLDRYGFVGGLGKRGMDHFAFTGGLGKRDSGEASGDLEEGKRGLDAYSFTGALGKRGLDRYGFVGGLGKRGMDDFAFSPGLGKKRMDSFMFGSRLGKRGMDRFSFSGHLGKRKMDQFSFGPGLGKRGFDHYGFTGGIGKRGFDHYGFTGGIGKRQLDPMLFSGRLGKRSSSEQEEEDVRQVEKRSTTEEQSSKSL

>Buccalin_A.kurodai

MIKNKGHRQFLVYVTLALSLGLALAEDATDPNHDTGSFDDVGAVSEEADLDPYSMSQELDKRPNVDPYAYLPSVGKRAFDHYGFTGGIGKRKIDHFGFVGGLGKRQIDALGFAGGIGKRYDSFAYSGGLGKRGMDSLAFSGGLGKRGMDSLAFSGGLGKRGMDSLAFSGGLGKRGMDSLAFSGG

>BursiconA_pfu

MACLGYTIAECNIRRVVQIVRYGNCQPKYVFTFACGGTCSSYTRPSQTQNTIERFCECCREEEKRMGRIMVLCPRDSNGFRRVPFYIQVPTSCRCRPCSILPDNIISPEDELLNGKKRSYTNSTKLNS

>BursiconA_C.gigas

MLVAFVFILGATTEISEARCNKRFITHVIRKRNCYPRALLSVACLGTCTSYTKPSVDNPGSVERFCECCRESSSRQETVNMICLDEATRGLTNISLSVMIPTSCMCRPCSVLPDTIVPAEQHIFQH

>BursiconA_Pfu2

MLEGLEWKKARKKEDIYRLCLFYKINKGSNRKDVSYHQRDEVTTTILVPSRSPNQGLIHIGCHFSREPSETVQYTTTTRDRSACQRRVIAHIVRYHNCQPRRILSLGCAGYCPSKVYVSRFVDNEVDTYCQCCRESQKIFRRMRFLCFRPSVGSRMHPVILPVMLPTSCRCRPCSFLPDHIVPSERYLMPIIQDNKRSINVTNNYFNPGPTTQDDFNDFLPDEPVITRAVIVTSSLDEHQTEEDHDNDNISVETDHMQPEPSAKGDADDRP

>BursiconA_A.californica

MKAQNVVKKNEGSSLVNAIISESPVSSSSSTCRHRIETTLIAVPGCVPKSLLITRCQGSCHSRSVPEFDTDRQEVRHVEHCSCCKPMVMKFRAVRFTCPRRRQRFFVKRFGFPFRCACRPCANVPEVTAEQPYDY

>BursiconA_M.californianus

MTEISIMVTKLIAASITLQVVASLCARQRTIHTIQYRDCRPKRVLSFACSGTCSSYSKPSSLQPGEFVKHCECCQENDTITRQTRVMCPNEGGETPFKYVVISVSIPVSCMCXPCSQFPLSNAQSEQDISPHESGPKIPPTFVFWYNELKTKKNFVTGK

>BursiconB_C.Gigas

MAKSGVFLTFLGVVVCVQGRLFRTSPETCSTERTEMDITRTLTVTHEGVLKTVDCVARVSVNECEGSCLSTASPNVHTYPDFKKDCKCCKEGRLVSRQIALQECYSNGVVLAGVQPTTSITEPADSDCFPCTN

>BursiconB_C.rhizophorae

MVTSGNLFVFLGIFACTGGILFRTRETCNTEQTEMDITRTLTVTHEGVLKTVECVARVRVNECEGVCLSTASPNVHTYPDFKKDCTCCKEGRLVTRQIALQECYSNGVVLAGVQPTTSITEPADCACFPCTN

>CCK-SK_Pfu

MKNCMECLIWTYISLAVCLTFALSLPVGNSKTDFDRISKIYNSLKMLQHKRNDFIKSENKDSFNRRNMDSKYSDDIGLFLALGEPIRVSDDNTNEGQNLEKRQGVWDFDYGLGGGRFGKRSFGDYSLGGGRFGRDVDHVDPDFEDNX

>CCK-SK_C.gigas

MKNSLEHLLISSVLVLCPLFTLTKGNSHAQNSIIQLSKLFSNLQDIHDAKQTQEENTKDQVRSDTRKQRGEIELILALGAPVHVESLQHNDKDTQTDTDKSAWDVDFEDDQQDVEKRQGAWDYDYGLGGGRFGKRFDYNFGGGRWGRDVDHVKQGQAKMKNSLEHLL

>CCK-SK_A.californica

MEPQLLSVVILVLCACAASALPTNSRREDLSHLVTLLGKLKNIERARQNSEGSAQAHAQEAWEGPRVSSFPETEVTADVIPDEVSAAKGYLGQRPATGVVNKRQGAWSYDYGLGGGRFGKRSYGDYGIGGGRFGRDVDHVDLSDATENEITS

>CCK-SK_GastrinII_Human

MQRLCVYVLIFALALAAFSEASWKPRSQQPDAPLGTGANRDLELPWLEQQGPASHHRRQLGPQGPPHLVADPSKKQGPWLEEEEEAYGWMDFGRRSAEDEN

>CCK-SK_H.americanus

MRWTSWTAAVLVVMAAFMLSGGVSAPARPSSLARVLAPVVRQRLEESHLPPALVEELVQDFEDPELLDFHDAAGKREFDEYGHMRFGKRGGGEYDDYGHLRFGRSLTHSDQHHHHDTTVN

>CCK-SK_C.quinquefasciatus

MARLSVTILATLILYLAYQTLASEAVPTGSGNSKLLSSSVDTSENLLSDENLNKLQTAWFKSAYNRRSSPVGGSDARAAVATYPVLRRTAPAVAAPNPMGYFADISLLDDEDIQKRFDDYGHMRFGKRGGEGDQFDDYGHMRFGRRQCAEPESPQSVESKEMSCKGAFGS

>CCAP_C.gigas

MVSFQQSFATCVGILTILSFCQCSQYSYADELLQKNENSKLGVKKLAALLANELLSEDKDITQKDKRVFCNGFFGCSNGKKRSNSMNLNPLDYPVDAPEPETKTDFRKRLFCNTGGCFGRRKRSTSQTLERRLNNRGSKDGNQ

>CCAP_A.californica1

MLPVDDTPSLTDLMTSGVAMSSGGGSKSPTFKRVFCNGFTGCGGRFRGRRRQLNEALGKRRLSQQRVGKRPFCNTLGCYNGGRKRHEEEEVESAKVEEQLKELVSTSSRLPVLSPRGGWNLDEKVKRLFCNGYGGCQNGGKRWGKSGMGTKQTGASSRNLALPLSDESLKKLSTMKRFFAGGYDNDMEGLIDSLKR

>CCAP_A.californica2

MTSSVLSPTSSFLLVLVLNVMLASIPLSAVTADEFFKQPAESKTTVKRSTSGESKPPRLLELIEMRDAVSSMLPVDDTPSLTRNFDGTSGVGDGLQAGGSQVPRLFKRVFCNGFTGCGGRFRGRRRQLNEALGKRRLSQQRVGKRPFCNTLVVIMVGVNATKKRR

>CCAP_M.californianus

MLIKKRPFCNGFFGCGNGKRSYDVPVIRDEQRQPQQKRLFCNMGGCGNGYGKRTLYSALLDRLQPVADDKSL

>CCAP_I.paradoxus

MTMMHSTPSCVPVGFFLQPVYVLLICTLVQAASSQAQESTSQIKSSESNDLSMKIRHLILRALEQQQQQQQQQAAAADGYTDQEDIALPPYKRVFCNSFGGCRSLKRLFNDEPGSLNPASEDSVQPTGEADIEKRVFCNSYGGCKSFKRVSANAMKNPALSKNRQR

>CCAP_L.gigantea

MICSHFHCLLLASFIVLTVSEDFSPLYLNENEDTNDLLKDTDSLPSSNNIYDSIPPARPPFPLLSLLFSKLKMEGSEKQAVIKRVFCNGFTGCGGRHRGRRRQYTARILKRPFCNNWGCGNGKRALEKVPVPFGKNLARKRLFCNNYGGCRGGKKRTLYSNWLGKLNGVADNL

>Cerebrin_C.gigas

MRTETCVTWVVCSLCLITLSVCRPIKDVEGEAFASDNEERLLLAAKLLQQMQQRQYGIRYYPIIPTHKRNLGTVDSLYNLPDLLYRGKR

>Cerebrin_A.californica

MFGYRSLLVLLVTLSLCLLLQSSHCSAVRTYGNDLDARARREIISLAARLIKLSMYGPEDDSFVKRNGGTADALYNLPDLEKIGKR

>Cerebrin_L.gigantea

MNSCTKCLLLLVFVGLTVLVKLSESSPLRPYQTQIEEKSRQDIITLAARVIKIAMYGSNQYDVIKRNAGTVDHLLNFPDLSVGK

>Cerebrin_L.stagnalis

MSFMQNVSLPYRTLLVVMAVLALTWLIQGTSASAVRHQEEKIRQEIIKQAAKIIKLSMMDSDWDPINKRNGGTADAIYNLPDLLEVGRR

>Conopressin_pfu

MPLFLYDSEACFIRNCPPGGKRSMDILQHGVRECLSCGPGNLGQCIGTNICCGDFGCLMGTKDSEICAKENDSSTPCSVKGRLCGVQNRGKCVADGICCDFDTCSLNSKCKINSDRGNSEILSLINQLLEDRDYRD

>Conopressin_L.stagnalis

MMSSLCGMPLTYLLTAAVLSLSLTDACFIRNCPKGGKRSLDTGMVTSRECMKCGPGGTGQCVGPSICCGQDFGCHVGTAEAAVCQQENDSSTPCLVKGEACGSRDAGNCVADGICCDSESCAVNDRCRDLDGNAQANRGDLIQLIHKLLKVRDYD

>Conopressin_A.californica

MSHSSMSPLSVRTFVLVAGLAVISFSVVADACFIRNCPKGGKRSMDMQLLGQRQCMACGPEGIGQCVGPNICCSPHFGCHIGTPETEICQKENQSTSPCSVRGETCGYRDSGNCVANGICCDSESCAANDRCRLRKETSRIGFDDTQSSRAEVLKLIQKLLRAKEED

>Conopressin_A.kurodai

MSPLSVRTFVLVAGLAVISFSVVADACFIRNCPKGGKRSMDMQLLGQRQCMSCGPDGIGQCVGPNICCSPHFGCHIGTPETEVCQKENQSTSPCSVRGETCGYRDTGNCVANGICCDSESCAVNDRCRLRKETSRSGFDDTQSSRAEVLKLIQKLLRAKEED

>Conopressin_L.gigantia

MCFSSFQYILSLFVFMCVIFGANSCFIRNCPTGGKRAIEASEIGHKCMSCGPGNVGQCVGPNICCGRFGCYIGTKETEICEHENDSTVACRVEGKLCGSRQQGQCVANGICCDSEACSQNPKCEEEEEETKEEHPREKILHLLQRILEENSFH

>Conopressin-neurophysin_C.gigas

MQKMECCKILHLSLPQVSLLLLMFTTLSSCCFIRNCPPGGKRSMGIVSQPTKECMSCGPGLLGQCVGPDICCGPFGCQMGTSESNICGKENESTTACAISGPPCGSRNQGNCVADGICCDTGACSFNTKCKLNSEPRDVQILSLLKTLGLIETKAYNNAGMS

>Cephalotocin_O.vulgaris

MSQNCFAIVQLLFVLFTVCSLFIATTDGCYFRNCPIGGKRATPMSEQGSNQKCMSCGPNGEGQCVGSNICCHKDGCIIGTLAKECNEENESTTACSVKGVPCGTDGQGRCVADGVCCDESSCFTTDRCDRENHRSMAMQKLLEIRDGIYYKK

>Conopressin_E.scolopes

MLLIVVLPLISLVEGCFWTACPIGGKRSTSEIKECMSCGPEGKGRCAGPNICCHKEGCVIGDLAKECQQEDEGTTVCEVKGLPCGAEGQGRCVADGVCCDTSACSTNSHCGPVQALSSSRRQELLTLLKRLINKVN

>Elevenin_pfu

MPKLSCEGLVLSLILICMVTYTFAKPRRRFCENFPFARRCIGVSAKRDVRGRYDMSKRRDDQVSTLDAVEKLLMEKSSLENEESYEDYGDGWDFINGKEDTMENDYPSNILKKIIKRKSVNTDIISQERMPTVWVDYRERK

>Elevenin_C.gigas

MQVTTIENVILAFVLFTMTVAGQIRARRRFCETYPFARRCLGVAAKRDVTGLHTENSREQRLLEIENMLENERFTERTESPETLFPTTYVKRSPVSLLEQILNNRRRFGLKRQSGSCGI

>Elevenin_Lottia

MNRTMAEKVFSIFGLLLIVLVLITDSRKLDCTKFVFAPKCRGVAAKRNTDYNFPIIPSHQRLQSKIVPSLEDNWTSEDDDSLQFLKGLRYLISKMNNKQPSPPYSTDDSRFWRKFFPSR

>Elevenin_I.para

MFRVHQSTFQKFCVWALVLLILVNLNVQAQGKFELTKKLNCRKFIFAPRCRGVAAKRMFPSSTSYPALKQQSVDERNYLNSNHDNTVREILLNYLLSRIEGRSVFDNGVNSDSPDSDLSSY

>L11_neuropeptide_A.californica

MPCTPNSHRLLLVTALCLLITSLFAQQKRRPRIDCTRFVFAPACRGVSAKRALDPRAVTLDENVAPSFSDLDDEILRSFLAYNRHKQQEQAAPRPDAPSSLLRLVAQRGLRHRTPHSLHRPVPWSVEQSFLKTGSDGALSWPNSIKSLQED

>ELH-like_pfu

MFTSFFQLDKNAIKKVGTFISPIRKRELTENNYEYMSNMRERAIYILAIMVVLSNIGGTVSENDDSGSSEEKPAFIEIEDSPEVSKRTYISLNGDMRSLAKMLMRHYGNRSVKRPVENYTSLRKKLYALGKRDAKPTLRDLLKDWIFKQTKRQRLSVNSALASLADMVSADGHRRMKEEMSSNHQRLLGLGKR

>ELH_C.gigas

MKMKLNVMAVIVTALFVSVDAFILQDQDNQPDLSEADSIPVEKRGRLSLTADLRSLARMLEAHRKRFIASRFPYDSIRKKLFRYGKRSPVPETLEYNEGEDRSNALDSEFPSLKVEEESPIYNIKRQRLSVNGALSSLADMLAANGRQRMMSEMAMNRQRLFGLGK

>ELH1_Lottia

MLKLTVLSVFLLYVIMTSYAMPRSYSQVPEELSSQDQSRDYYEPLNAILHRLRELILMQPHSLRETIRYTQITPTTPIPIAMKKRSRLSINQELKSLANLLVLRENKRREAQKTKLRSKLLSIGKRSMPQNQDRDHVIEETPVSDRDMFLTELFAEISEQEMTYVLGQVLGSEIAAKGGNEYQGQERLPYEI

>ELH2_Lottia

MKMEWNMNKSLSVLVLCLFIFVVDDAHSYSMNRKSESSRHIRSASDIPDDRIQDSKASFHENYFGTDPDHQNLYRERLESMFSDQKKRMDIHQQQVSLKNLINLIHETSNRIRKLSKTERPDMDDDENVRIKTVVKTWSKNDPENGRDNVISGYKTEVGNPKRAGRLSINGALSSLADLLVSENQRRDRLESMELRQRLQYLG

>ELH_Aplysia

MKRPNNRPTNTMSLILCLTLSSLCVSSQSASVHGKNFATNRAVKSSSPFVVLSPDDNVVSMSGENGYRSALREAFDKSSRDYDDNGEDVFSNEKRRLRFHKRRLRFDRRDQDEGNFRRFPTNAVSMSADENSPFDLSNEDGAVYQRDLRAPRLRFYSLRKRAAGGMEQSEGQNPETESHSRRKRSVLTPSLSSLGESLESGISKRISINQDLKAITDMLLTEQIRERQRYLADLRQRLLEKGKRSSGVSLLTSNKDEEQRELLKAISNLLD

>ELH_Lymnaea

MKMSGLLSKPDYGVVGIVFTVVFCCWCSSSTTHALSIAEPGRDRYDKRSPTGHGVEVVESGEDYGSNRPQPVYGDEDEEDSADVYVGSDESSSGEKTRLTAAKRRLRFNKRRLRASKRRLRFHKRRVDSADESNDDGFDRKAREPRLRFHDVRKRSATAEEGSENAEIEESHLGNSRSRRSAGSAPSSANEVQRSKRLSITNDLRAIADSYLYDQHKLRERQEENLRRRFLELGKRGSAFFDHIPIIFGEPQYDYQPFK

>ELH-like

GSGVSNGGTEMIQLSHIRERQRYWAQDNLRRRFLEK

>ELH_A.dactylomela

LRKRAAGETEQSEGQNPETESHSRRKRSVLTPSLSSLGESLESGISKRISINQDLKAITDMLLEEKIRERQRYLADLRQRLLQKGRKSSDVSLLTSNKGDEERELLKT

>ELH2_A.parvula

AISLLMCLILSALCASSESAVVHGDDFAAERAVKSSPYLVLSPADEMVGMSTANEAFDKSPSYYDDDDDDFVNNEKRRLRFHKRRIRFHKRPQEVSGLKPVMMSRASASADENSLFDLYNTDGAMYQRELRAPRLRFYSLRKRAAGDEDKAEEHNPETESHSRRKRSALTPSIRSLRSSLESGIAKISINQDLKAIADMLIVEQKQEREKYLADLRQRLLNKGKRSSEVALAASDKGDEERELLNTLSNLLE

>Diuretichormone44_P.dumerilii

MAMPTPLIQGLLSVCVLIALISLSLALNDDSAEFESSEGLHKRAVKLSVNNGLSALANSMKDKELEDGIQTSHAQLFRMGKRPSFSFNQDLKSLADAHGMHSAENSRARLLNLGKRVMFSVNGGLQSLADSMNEGPSHGNLMALGKRPAFSVNHGLDTLAQAMGSDGSSQSKLLSLGKRPAFSVNHGLDTLAQAMGSEGSSSQSKLLSLGKRPAFSVNHGLDTLAQAMGSEGSSSQSKLLSLGKRPAFSVNHGLDTLAQAMGSEGSSSQSKLLSLGKRPAFSVNHGLDTLAQAMGSEGSSSQSKLLSLGKRMQLSFNQGLQSLADSMSNDGGRSRLMNLGKRGQLSINNELQSLAHSFKPNGDSHMSLLNLGKRAPLSVNAELHSLAHSFRPNSQASLLDLGKRAPLSINAELHSLAHSFNGDGEGRARLLNLGKRVPLSINAELQSLAHSYQPNSQMRLMSLAKRVPLSVNQGLVSLASSMRGDRAEASRNFLQSLGKRAPLSIGQEMQSLSDAYNSHSGNPLLNLGKRMALSVNHELRTLGDAMRHGQAAPSLLSLGKRFDNQNDHEHVRIMKRSPGARRLSINSELWSLAQMARDARSHEDRKNAQALLDHLGKRSV

>Diuretichormone-I_A.mellifera

KRLESKRIGSLSIVNSMDVLRQRVLLELARRKALQDQAQIDANRRLLETIGK

>ELH-like_A.gossypii

MRILTIAWILVVVTWCCDSAVIYEPMVQNTRYYEPRIQDLELLDKNFFDMASIEKKNGAMQGESPRSRPSLSIVNSLDVLRQKLMYEVARRHVDENQKVLSQNHQILKNLGKRSLFPFLEVPRRF

>Diuretic_hormone_44_D.melanogaster

MMKATAWFCPVLLTLLCATRLVCTAQRGAVGAGGAAGGSGAAAGGAEVGGSGRTNGYPLDYPDGTRNSQDDFLLAKRNKPSLSIVNPLDVLRQRLLLEIARRQMKENSRQVELNRAILKNVGKRVVLRGGGGGGGSGAGGLAPKVSRRYRQQWPVERELERERQRERERERDAVREEQLDRQQLLPWKHFPSQLWSYGWALSPYKESSQLQFADSQQSASTGPQSQALPKQLQLLSYAKKPLDVAGMSLARHRVSGNEANETNHENDDGNGASKNPARYVDDGDNEGEDSYNDVGTEGVGLGLGMGVGLGLERFEVLEDKPNWANEEPNELVVVNANDRVPWSFPYRFHKSQHNVN

>Diuretic_hormone_44_A.gambiae

MKATLWLWTAVMALLCVSLQVSALPYSDSRVVQENSLLSLKRTKPSLSIVNPLDVLRQRIILEMARRQMRENTRQVELNKALLREIGKRSSNLYDGSEYPPDYTYYDRKLYNQHVQPMPDHGSPAEDELEAVVESLLRNQGLTARDGNYAGKLQRTRFINGRPMPAAAAASLRGMASNPMAQHDQPQQLSSLFSNEEEREDLKTKDQALRQSLSVDQDLDQTNGGNGEEDEDQDGHQAGRVSGTGTGVLKPMQDTDNLLEQPNQPSVSSLSSSSSSDRNSGTEFGPRYVYGMYKNRYAN

>Diuretic_hormone_44_Bombyx_mori

MMWWAVWCAAMVAGSVFTAAAPPTDSIDLMQMDPSLADDESLGFAMQSLSGRYAAAPWLYLLADVSHDPQRMAEFSQSSGRARPKRKMPSLSINNPMEVLRQRLLLEVARKQMREANQRQAVANRLFLQNVGKRGAWGEPASYLYNN

>Urocortin_H.sapiens

MRQAGRAALLAALLLLVQLCPGSSQRSPEAAGVQDPSLRWSPGARNQGGGARALLLLLAERFPRRAGPGRLGLGTAGERPRRDNPSLSIDLTFHLLRTLLELARTQSQRERAEQNRIIFDSVGK

>Corticotropin_releasing_hormone_H.sapiens

MRLPLLVSAGVLLVALLPCPPCRALLSRGPVPGARQAPQHPQPLDFFQPPPQSEQPQQPQARPVLLRMGEEYFLRLGNLNKSPAAPLSPASSLLAGGSGSRPSPEQATANFFRVLLQQLLLPRRSLDSPAALAERGARNALGGHQEAPERERRSEEPPISLDLTFHLLREVLEMARAEQLAQQAHSNRKLMEIIGK

>FFamide_P.fucata

IFHFRCFRKLSLCLLVIATVLVLVQYSSSEKSRGLSRLVGQQPLLFGRRGVNPNMNSLFFGKRAAEQSVLEDILMEKCTNVLATCQRIMQGRDSVSEEIWYV

>FFamide_C.gigas

MKIYSIIPIVIALVAVIVLKTSASKENSRGLTRLVGQQPLLFGRRGMNPNMNSLFFGKRAVDRSTLDDIIVEKCSRIMAACQEYAHERMGEDDI

>FFamide_H.asinina

MNCKIPCLLLVLTFCIMSFTHAQANPRNLNRLVGQQPLLFGRRGVNPNMNSLFFGKRAPSMNNMDVRTMCNAVLSACAAWQADMTDN

>FFamide_B.glabrata

MIQQPLLFGRRGVNPNLNSLFFGKRSSGMEQLVSIREIKHACSVLNSYFDNMESLIGEDESQM

>FFamide_L.paradoxus

MSGRRNLLVPASLVLILSAILFFGHETQQVEAAYSPTRGQQNTHSFGRRGLNPNVNSLFFGKRGGSEQEALSNTEMGRKCLAAMSMCNMYFETNTINES

>FFamide_P.acuta

MKLLSVALSLAIILAMCESTEASKALKDLLEQQHLIYGRSKITPSMNSLLFGKRSQAENFRDMKRQLNTYLESMDALINDDESQV

>FFamide_L.gigantea

MVAKGTVGILIVLFNTICANIDLNYNQVPVANPLLFGRRGINPDMSSLFFGKRSGNSDHRDLRKMKDTCKAVLSSCKILFSDYEDDTVRNKVQDGFGRFK

>FCAP_pfu_aug1.0_28033.1_11883.t1

MKMHKSHSHMTLKLFTICIRISLNVMYLYGKKNPKISDYGISTFSPDASNIRYYGNSLRYYFHKGIVTWIHKASPGSTKRHLDPQSVTWIHKASYGSTKRHMDPQSVTWIHKASPGSTKRHLDPQSVTWIHKASPGSTKRKYSNT

>FACP_C.gigas HG965451

MKMHSVLYLGLLFSICYQTSAGDNKVQRYDPSEYNDLLQLLQGWTLTTPSASMTTLPPLDNLGGAVVHKGYRESGDIEGDLYQQEPKSDNGVPENKVSSDSLDEDYFERFQDKYPSESEFDKVSETYNDEDPSDDKEKRVLNSLGGFSLHGYKRGLSSLGGLNLHNFAKRGLNSLGGMSLHDFKRGLSSLGGMSLHNFNKRGLNSLGGFGLHNYKRALSSLGGFSLHGYKRRLSSLGGMSLHGGLNKRGLYRPGRMSLPGGFSKRGLSSLGGMSLHGGFSKRGLSSLGGMSLHGGFSKRGLSSLGGMSLHGGFSKRGLSSLGGMSLHGGFSKRGLSSLGGMSLHGGFSKRGLSSLGGMSLHGGFSKRGLSSLGGFRLHGYKRALSNLGGMNLYNFKRSLNDLEKNNNSESPYDNLESSDIMSGTMDASKHKRSVPSEPRTDN

>FACP_Pinctada_ingi|262320415|gb|GT281393.1|GT281393 pmaximaP0014K14_687 Adult silver lipped oyster (Pinctada maxima) Pinctada maxima

MKRGFGIHGGFKRGLSSLGGFGLHGALKRGLNSLGGLSLHSGFKRGLSSLGGFGLHGGFKRSSDSEDEAFNGEEKRALSSLGGMRLQQYKRDVSSDSNKLNTNKDTEEKLKSKRSVDSNDNLLTKDSEHINKVYMK

>FACP_A.californica_gi|325297025|ref|NP_001191518.1| feeding circuit activating peptides precursor [Aplysia californica]

MTFAASFRALLCVLFCAALVHCKTRTKRYVPHSELWRILAVVDELQREQAAEQRQEDALALALRSDIAGGGGGGQLADNVRWFPETYDYGALADRDVDKRVFDSLGGYEVHGFKKRGSLDAIPQDTDASSDKRALDSLGGFQVHGWKRALDTLGGFQVHGWKRGSGAEKRQVDRLGGFQVHGWKKRALDSLGGFQVHGWKKRGTGGQMHASSPRVVPWGSRSLLADTQSGHRWKRDTELVENRQTTGQQTEVNKRALDSLGGFQVHGWKRSGEAGKRQVDSLGGFQVHGWKRADDQGKRALDSLGGFQVHGWKRFDNSAGEKRALDSLGGFQVHGWKRAGDKKSLDSLGSFQVHGWKRFDNDISGQKRSLDSLGSFQVHGWKRSDQDNKRALDSLGGFQVHGWKRADDDGKRSLDSLGSFQVHGWKRADEDDKKSLDSLGSFQVHGWKRGDEDDKRSLDSLGSFQVHGWKRADEDDKRSLDSLGSFQVHGWKRSDEDDKRSLDSLGSFQVHGWKRSDEDDKRSLDSLGSFQVHGWKRADEDDKRSLDSLGSFQVHGWKRNSPGLKRALDSLGGFQVHGWKRNNEYYSGAENEKRALDSLGGFQVHGWKRDQPGEKRSLDSLGSFQVHGWKRNLNNLGSFQVHGWKKNSADEMGDKPGVESYQDNSGKILSGKAQEFEGGDETGDIHGVVRTLSGVDASGKERENIKELDAKFKTNDGGVGVEHIFVDNVKSADDDVPSAGQM

>FACP_Lottia_gi|163312613|gb|FC557999.1|FC557999 CAWC939.fwd CAWC Lottia gigantea from head (L) Lottia gigantea (AMQO01002371.1)

MLSKVYMLCLEIFICCKISSILTSQLETKVTQKNEVRREKELYPLMNKKSEESFTISDYDDVDESAEKRYFDTLGGAFVHSFKRNSNPSKNKQFFEKLKNLLKVSKESSHKRALDDLGGINVHSFKRGLDDLGGVNVHGFKRGLDDLGGVNVHGFKRGLDDLGGVNVHGFKRSLDDLGGINVHGFKRGLDDLGGVNVHGFKRGLDDLGGVNVHGFKRGLDDLGGVNVHGFKRGLDDLGGVNVHGFKRGLDDLGGVNVHGFKRGLDDLGGVNVHGFKRGLDDLGGVNVHGFKRGLDDLGGVNVHGFKRGLDDLGGVNVHGFKRGLDDLGGVNVHGFKRSLDDLGGVNVHGFKRGLDDLGGVNVHGFKRGLDDLGGVNVHGFKRGLDDLGGVNVHGFKRGLDDLGGVNVHGFKRSFDGLGGMNVHGFKRGFGINVHRFKRGLDDLGGIHVHSFKRNFDDLGGMNIHGYKRALRDLNTIQADDFKRKLNSHGGMNVHGNKRGFDDLGGIDVHSFKREAGDLDETESNHVQHIENNASTNKR

>FACP_Misuhopecten_gi|266704586|gb|GT570646.1|GT570646 dlsmcb0_005726 Japanese scallop adductor muscle normalized Mizuhopecten yessoensis

LGGMWIHGYKKRGLDRLGGAYLHGFKRAMDNDGDNSKRSLDRLGGAYLHGFKRAMDNDDVDSKRSLDRLGGAYLHGFKRAMDNDDVDSKRSLDRLGGAYLHGFKRAMDNGEVDSKRSLDRLGGAYLHGFKRSMDNGEVDTKRSLDRLGGAY

>FACP_Mytilus_gi|223024814|gb|FL490716.1|FL490716 Mg_Nor01_50P13 Nor01 Mytilus galloprovincialis

LEDTVNPNSAEELRMFLESNMEKNNEEKRGLDTLGGMSYLKRGLDTLGNMGFHSYKKKGLDTLGNMGFHDFKKRGLDTLGNMGFHDFKKRGLDTLGNMGFHDFKKRSLDT

>FMRFamide_pfu_aug1.0_182.1_58006.t1

MRAWSYVGLLAAIIASWFGGKVRADDIERWSRWCFMNQEACSSVLSEYLEKRFLRFGKRALAGDAFLRFGRSYPREDKRFLRFGKRDDSGEIGVDEILKAALSRIQNIERASGVKIRKRRSAGPEPLKDVPEEPEQDNKEADDKDEEDKSADIKKRETDEAKDKRFMRFGRDSQDAEGGEGETEKRFMRFGRDPDADAEKRFMRFGRDPSEADKRFMRFGRDPDKRFMRFGKRFMRFGRDSLEADKRFMRFGKRASDLEDNDATFAKEDKRFIRFGRGGEQDKRFMRFGRSGGEDDKRFMRFGKSVDDEKRFMRFGKSGEADKRFMRFGKSIDDEKRFMRFGKSVDDEKRFMRFGKSVDDDDMEADKRFMRFGKSVDDEKRFMRFGKSVDDEKRFMRFGKSGDQEKRFMRFGRGGDNADKRFMRFGKKSDDKRFMRFGRDSADKRFMRFGKK

>FMRFamide_Crassostrea_gi|405957910|gb|EKC24087.1| FMRFamide neuropeptide [Crassostrea gigas]

MGTWTYLCLLVAFLLNWFTIETSANDLIDDCYRNPELCQEVGILFGQQQPVDKRFLRFGKRALSGDHYIRFGRNSDDKRFLRFGKRGEQGSVEDDLREALNKVIKFKQETGLHLRKRRSADPPLVKDVPEDKDSNSTEKEDSASEKHKRETDEVSEENKRFMRFGRTPAEDDPTYMKRFMRFGRNPDLEKKFMRFGKDGNEKRFMRFGKREDNDNMVTDDKRFMRFGRDPKDDTLMERFVRNGRSGDDKRFMRFGSTAKKIISR

>FMRFamide_Mytilus edulis CAA10949.1| neuropeptide FMRFamide precursor

MWTKSYATLIVAAIINWISVKVHADELSRWCLDNQEICSNLIQNLRDTDDTNAQKRNFLRFGRALAGDHFFRFGRSPYQTEDKRFLRFGRSGGGGGFDNVGLADILKAALVKVESANQNGLKIRKRRSIDAVKDVPEKKSVTDNTEPEAEIKKRNVDNSYATNEDSADENLKAADKRFMRFGKRFMRFGKRENADKRFMRFGKRGDEDFGEEEGDDTYGVEDKRFMRFGRGGTEDKRFMRFGRAGEDKRFMRFGKRADENFGEDEGDETYGVEDKRFMRFGRGGTEDKRFMRFGKSMDNDDEKRFMRFGKSAEADKRFMRFGKSLEADKRFMRFGKSGDDEKRFMRFGKSVDGEDKEKRFMRFGKSTEDKRFMRFGRDPAEKRFMRFGKSTTEDKRFMRFGRK

>FMRFamide_California sea hare_A25790 FMRFamide neuropeptide precursor

MRPWCQLALLACLSLKWLTSHVTAESFLCDDSELCENGYLRFGRSMSVEEPHFRLERRSYPPVVYHKRFLRFGRSQEPDIEDYARAIALIESEEPLYRKRRSADADGQSEKVLHRARREAESEHKSLEEVSPDTKQDVEKRDADDVLDAEKRFMRFGKRFMRFGRGSSDDDESGDDDVQDLTDIGDGLGGEGEVNKRFMRFGKRFMRFGKREDGEPDKRFMRFGKSMADNDLDKRFMRFGKRFMRFGKSLPDSEVDKRFMRFGKSVDGDVDKRFMRFGKSVDGDVDKRFMRFGKSVDGDVDKRFMRFGKSVDGDVDKRFMRFGKSVDGDVDKRFMRFGKSVDDDVDKRFMRFGKSVDGDVDKRFMRFGKSVDDAVDKRFMRFGKSVDSDLDKRFMRFGKSVGSDEVDKRFMRFGKSVGSDEVDKRFMRFGKSLGTDDVNKRFMRFGKSLGTDDVNKRFMRFGKSLGTEDVDKRFMRFGKSLGTEDVDKRFMRFGKSLGTDDVDKRFMRFGKSLGTEDVDKRFMRFGKSLGTDDVDKRFMRFGKSLGTEDVDKRFMRFGKRFMRFGRSVGDSKYRSASSESVMTTDSKQTTEQATNKS

>FMRF1_Haliotis asinina_ACD65487.1| FMRF1

MRPWTSVALLAVVLIKWISCINGFSDFCNKPVNSRICAILSGGPPQNEEKRFLRFGRTLAGDSFLRFGRQFYRIGKDGDDMEKRFLRFGRSDPDLDDVIRASLLAYSLDDSPNNRRRRSVATAPVEAKAVEAGNKDIEKRDADELTSEDKRFMRFGRSGEDEKRFMRFGKSGEEEKRFMRFGKSGDAEKRFMRFGRAGEEEKRFMRFGRAGEDEKRFMRFGKSGEEEKRFMRFGRAGEDGEEIEDEDEGIEADKRFMRFGRDGEDEKRFMRFGKSGEDEKRFMRFGKRFMRFGRDGQDKRFMRFGKRFMRFGKRDSGEGSSEKAETAES

>FMRFamide_Lymnaea stagnalis AAA63280 FMRFamide precursor protein

MYSPTLIVCLSFFHSAVTKRFLRFGRALDTTDPFIRLRRQFYRIGRGGYQPYQDKRFLRFGRSEQPDVDDYPRDVVLQSEEPLYRKRRSTEAGGQSEEMTHRTARSAPEPAAENREIMKRETGAEDLDEEKRFMRFGRGDEEAEKRFMRFGKSFMRFGRDMSDVDKRFMRFGKRFMRFGREPGTDKRFMRFGREPGADKRFMRFGKSFDGEEENDDDLYYNESDADSNDDVDKRFMRFGKSAEEKRFMRFGKSQDASRDKKEFLRIGKRESRSAEVENNIQIAAKQS

>FMRFamide_Loligo pealei_ACI22791 FMRF-amide precursor

MRCWSPCSLLVVIVIYCLSSHTSEAFDLAQACVESQRLSLLPICDTIFAVQQEGAQQSADDGMRSKRFIR
FGRALSGDAFLRFGKNVPDLPFEDKRFLRFGRAAPQLDDLLKQALQRVESLQKADETSVRRKRSTDAAPQ
NNAENPEQKNDSAKITKRYIDDVEDSDVKRFMRFGKRFMRFGRNPSDAGNKLTEKRFMRFGRDPEKRFMRFGKSDDKRFMRFGRNPSDVEDELEEDKRFMRFGRGGEDDEEEAEKRFMRFGRDPEKKFMRFGKSGEDKRF
MRFGRNPDEQEADKRFMRFGRGGEDDEVSTEDKRFMRFGRSADKCKGCLEG

>FMRFamide_Lottia FMRFamide Lottia gigantea v1.0 sca_20 : 1727885 - 1727986

MKSWSSVGLGLVLTLKWLCTISLADALDCSLSENGRGLCDLMGDGGSADKRFLRFGRALAGDGFLRFGRQFYRIGRSPTFEDKRFLARFGRAGVDESLSDLASAFRPVMQYGPYKNDILYRKRRSAETSVAPKDAESSENSEIMKRDATDPQAEKRFMRFGKRKRFMRFGKRFMRFGKRDGEEEKRFMRFGRDPDKRFMRFGKSIDNNKRFMRFGRDGEDEKRFMRFGKSVETQSLDDEDVDNIDSYDKRFMRFGKRFMRFGRDIDDPEYEPEEQKRFMRFGKRFMRFGKRDGEEEKRFMRFGKREGEQEKRFMRFGKRDGEQEKRFMRFGKRDQTVEKEDKTATA

>FxRI_P.fucata_pfu_aug1.0_810.1_22317.t1

MHIPPIHGLFLFTISFGYVLAKSVNEIENELDLIKDVEAGSKLFNHNNKFTGGIDSNPYTFDPDTSDDDNSTKGKRYSSFVRIGKKDNEKRLSSFMRIGRSGLEGDWSDDVVPIPSDNKRVSSFVRIGKGNTEYEDPTKRLSSFVRIGKNMANEGVYMDDNAKRFSSFVRIGKNTHDIDDQTYPEKRRISSFVRIGRGPVKRLSSFMRIGRGLTNESAQTDEKSTGDDMSSDSEVSKRGFIRIGKIPSSAFMRIGRKALLAQLMKDRYLRPSRLGQSSFVRIGKRDGNSDFX

>FxRI_C.gigas_EST CU987545

MLRPHHVIIVGLFYCYTTNAEINENKLLHPIKTEEGANEILGDKADDKRSRGFFRIGKKSAVENEANDKKFDSKTVKEEDNYIPEKIQLIRVVNSESETPIYVPVEFDPESSDDTADEDEKRASGFFRIGKSAENVDKRKGFFRIGKSVDQNPMNKKASGFFRIGRTPIDKRGKGFFRIGKSLNEMDEKRASGFFRIGKSALNDKRSRGFFRIGRSKGFFRIGKAFPLDGEKRASGFFGLAEIHLKNEEKASKFFRIGKSVNSKEENDKRASGFFRIGKKCSGDSDKAGDNLTEDKSQSNPENEDTSESFNRNSDEPVRRASQFFRIGKSSSNKVTKRSSGVNSSPEQNLNLNKRAFFRIGKVPTSAFMRIGRQHLLQSLVSDPLYRNGRIQQSSFIRIGKRSMSDNHLIDDEQSDSSL

>FxRI_L.gigantea_1 ESO97689.1

MECDESSVRRHQPILWSKDSLLSVIDIGQHLHMTTFDEMIQLTRQIMEINLVFSLLLVCSISFVLSHPFTDDSNNDDQDLKSAIQDGDTVPQAVKRPSSFVRIGRNPSSFVRIGKAFGRFIRIGKNDPNKRLSSFVRIGKSDPNKRISSFVRIGKSQEFNQEPEKRQSSFVRIGKSPELNENEIPNKRYSSFVRIGKSMDDGSLENPDKRYSSFVRIGKNIENELTNAGLEKRPSSFVRIGKSYFAEPGDMDAEKRLSNFVRIGKSGLEEPEMEQKRAFVRIGKIPSSAFVRIGRMPLYDAILQKPLGYYNVARRMGKSSFVRIGKRNNEA

>FxRI2_L._gigantea_2 EST FC795696

MDDGSLENPDKRYSSFVRIGKNIENELTNAGLEKRPSSFVRIGKSYFAEPGDMDAEKRLSNFVRIGKSGLEEPEMEQKRAFVRIGKIPSSAFVRIGRMPLYDAILQKPLGYYNVARRMGKSSF

>GGN_C.gigas_EST CU996337

MRNPSSFVLFSATLCVYFTVQISSVSASKCKGPWANHMCFGGNGKRSWSPPVQEPEMNRKDDELGRTMLRNVLLKRLNTYPSMSSYYSDSQSFYPMGSDFTEEGDSMSRENELRQLLKEQILRKEMAALVGDDDVYE

>GGN_C.virginica_EST EH649000.1

RNPSSLVLFSATLCVYFTVQISSVAAAKCKGPWANHMCFGGNGKRSWTAPLPEVDKRDDDVGRTLLRNVLLNRLDNYPPQGGYYSDSESFNPIGSDSTEEELPYNREQQ

>GGN_A.californica_EST GD211276.1

MELTFGSSFTVALLSCLYISLITCPVVTAKCHGRWAIHACFGGNGKRSDPNMSPSTDELPPTLLRQLLVFRRSTSQQ

>GGN_A.kurodai_EST EY423989.1

MELTLGSSFTVALLSCLYISLITCPVVTAKCHGRWAIHACFGGNGKRSDPNLSPSADELPPTLLRQLLVSDVQRLSRLLREPEAPVEDAELNPNPEFEPEQSNEQLMPRLAGIPPHW

>GGN_D.pealeii_EST JK324610.1

MGRHFILFVLTSALLFCISNAVKCKWYDHICLGGNGKRSAMGQSEQDQQLLKILTQEFVGQPHKRESLLTDDDDDDDDDTLFPHAKPFDQVYGRANLSPAIKRLAKLILPDETQN

>GGN_L.gigantea_EST FC746004

MEVRCTTLFAVTSLLYLTISVSLVSGKCSGRWAIHACFGGNGKRSDPSLTDNTENSRQETLLRQILLPQTYEYHKSNDALLQDDDINTYEKSHEESQRMRDLRALNILLKTLMMEQKVRTENSVMA

>GnRH-like_Pfucata_pfu_aug1.0_23475.1_47585.t1 GnRH-like

MFSKLQILSVALVLVLVLHSVLAQNYHFSNGWHPGKRTFDTNSCQFRPKVKSLIARMIEVRMSSFSSKLGSLLSFPLENSFYKAC

>GnRH_C.gigas_gi|325494719|gb|ADZ17180.1| GnRH-related peptide precursor [Crassostrea gigas]

MKVSPCTQVIVMVLTLGLLCEVHAQNYHFSNGWQPGKRSYRGCTVRPEIRSILIKIIEDEVERIQKCSHSNIEDVFSLIQEKTGVDAREV

>GnRH_Uroteuthis edulis_BAH09303.1| gonadotropin-releasing (Swordtip squid)

MSTSPVTSTLRRMVFLTCAIFLLSLCMQTQAQNYHFSNGWHPGGKRSGIPDMQCHFRPQTKALIEKLLDEEITRILTTCTNTVNDIADLQ

>GnRH_Octopus vulgaris_BAB86782 GnRH-related peptide precursor

MSATASTTSSRKMAFFIFSMLLLSLCLQTQAQNYHFSNGWHPGGKRSALSDIQCHFRQQTKALIEKILDEEINRIITTCTGPVNEIADL

>GnRHMizuhopecten yessoensis_BAH47639 GnRH-related peptide precurso (Scollop)

MSSYTQILVAQLLLAGLLVAVVSGQNFHYSNGWQPGKRAPMMTSGTQLCSFRPHIKALLLWIIEDEVKRIKSCGSSGYDDIINLLQSKQSGLPLSSGMPSDSQ

>GnRH_Aplysia californica_NP_001191482 preprogonadotropin-releasing hormone

MACRITSATTTLFSILLLIVIAELCSAQNYHFSNGWYAGKKRSSASLLRPEALGSSSSSSSNSGLDVTDADSSRGGVSSLSGRGFGVGELRLVDSPCSIRLETLALINKLMQEEAARIQRTCVANVPSGLRELLEGAASKLESENKW

>GnRH_Tupaia belangeri_GnRH AAB16838 (northern tree shrew)

MASSMLGFLLLLLLLMAAHPGPSEAQHWSHGWYPGGKRASNSPQDPQSALRPPAPSAAQTAHSFRSAALASPEDSVPWEGRTTAGWSLRRKQHLMRTLLSAAGAPRPAAVPIKP

>GnRH-like_Octopus vulgaris_gnrhrp AB037165

MSATASTTSSRKMAFFIFSMLLLSLCLQTQAQNYHFSNGWHPGGKRSALSDIQCHFRQQTKALIEKILDEEINRIITTCTGPVNEIADL

>GnRH_Loligo edulis_GnRHpreproform AB447557

MSTSPVTSTLRRMVFLTCAIFLLSLCMQTQAQNYHFSNGWHPGGKRSGIPDMQCHFRPQTKALIEKLLDEEITRILTTCTNTVNDIADL

>GnRH_Mizuhopecten yessoensis_pyGnRH AB486004

MSSYTQILVAQLLLAGLLVAVVSGQNFHYSNGWQPGKRAPMMTSGTQLCSFRPHIKALLLWIIEDEVKRIKSCGSSGYDDIINLLQSKQSGLPLSSGMPSDSQ

>GnRH_Aplysia californica_GnRH EU204144

MACRITSATTTLFSILLLIVIAELCSAQNYHFSNGWYAGKKRSSASLLRPEALGSSSSSSSNSGLDVTDADSSRGGVSSLSGRGFGVGELRLVDSPCSIRLETLALINKLMQEEAARIQRTCVANVPSGLRELLEGAASKLESENKW

>GnRH2_Homo sapiens GNRH2 NM_001501

MASSRRGLLLLLLLTAHLGPSEAQHWSHGWYPGGKRALSSAQDPQNALRPPGRALDTAAGSPVQTAHGLPSDALAPLDDSMPWEGRTTAQWSLHRKRHLARTLLTAAREPRPAPPSSNKV

>GnRH_Cyprinus carpio_salmon gnrh AF521130

MEGKGRVLVQLLMLVCVLEVSLCQHWSYGWLPGGKRSVGEVEATFRMMDAGDAVLSIPADSPMEQLSPIHIVNEVDAEGLPLKELRFPKRRGRV

>GnRH-like_Ctenopharyngodon idella_salmon gnrh EU981285

MEWKGRVLVQLLMLVCVLEVSLCQHWSYGWLPGGKRSVGEVEATFRMKDAGDTMLSIPADTPMEQLSPIHIVNEVDAKGLPLKEQRFPNRRGRV

>GnRH_Fundulus heteroclitus_GnRH1 AB302265

MAVKTLSLWLLLAWTVGLLSLGSCQHWSFGLSPGGKRELDVSPDRLDSIFEGLAHVGAPCSVPGCAEESPFAKIHRLKGLLVRVHEREHGHQALKQ

>GnRH_Rachycentron canadum_salmon GnRH AY677173

MEAGSRVMVQVLLLALVVQVTLSQHWSYGWLPGGKRSVGELEATIRMMGTGGVVPLPEEASAQTQERLRPYNVINDDPSHFDRKKRFPHN

>GnRH_Oncorhynchus masou_GnRh S44614 (Cherry salmon)

MDLSSKTVVQVVMLALIAQVTFSQHWSYGWLPGGKRSVGELEATIRMMDTGGVMALPEETGAHIPERLRPYDVMSKKRMPHK

>GnRH1_Petromyzon marinus_GnRH1 AF144480 (Parasitic lamprey) (1)

MALRGQSLTLLLLATALLVSLNYAQHYSLEWKPGGKRDLEVSHTRELEQELEPPSNAFECDGPECAFSRVPNTKLIRELASYLSQRNYDRKGALK

>GnRH3_Ichthyomyzon fossor GnRh3 AY307174

MALRGQSLALLLLASALLVSLTHTQHWSHDWKPGGKRDLEAMRPLLEELEAPDSAFECDGPECAFARVPSSELDREIMSYLSQKNYQRKVMK

>GnRH3_Petromyzon marinus GnRH3 AY052628 (III)

MALRGQSLVLLLLASALLVSLTHTQHWSHDWKPGGKRDLEAMRPLLEEELEAPNSAFECDGPECAFARVPTGELVREIVSYLSQKNYQRKVLK

>GnRH3_Geotria australis_GnRH3 AY307172

MALRAQSLALVLLSASALLVSLTHSQHWSHDWKPGGKRNLEAMRPLLEQELEPPSGAFDCDGPECAFGRVPSGELIREIVSYLSQKNFQRKVLK

>GnRH3_Mordacia mordax_GnRH3 AY307173

MAPRAQSLALLLLVSALLVSPTHSQHWSHDWKPGGKRDVDATRPLLEELEPPSSAFDCDGADCAFARVPSSELIRDLLSYLSQKNHQRKVVK

>GnRH2_Thunnus thynnus_GnRH2 EU239502

MCVSRLVLLLGLLLCVGAQLSNAQHWSHGWYPGGKRELDSFGTSEISQEIKLCEAGECSYLRPQRRSLLRNILLDALARELQKRK

>GnRH2_Epinephelus coioides_GnRH2 FJ550191

MCVSRLVLLLGLLLCVGAQLSSAQHWSHGWYPGGKRELDSFGTSEISEEIKLCEAGECSYLRPQRRSILRNIILEALARELQKRK

>GnRH-like_Alvinella pompejana_CAGA34466 mRNA sequence

MDLRMTTIAVTLTVLVIMSSVHSVYSQAYHFSNGWFPGRKRSIDVYSSSPYRMDSALKSSVKECQIRPQVEQLVTDLIQTELVRIVNECHLV

>GnRH-like_Lottia_FC805607

MMPVPLKYFGLALTLALVTELAVGQHYHFSNGWKSGRKRSGGVSNLCEMRPELINYINTLLSEELNRIKNTCNLNTEDKDSDVDFSEGAFSRLQNGLKLAADRKWKK

>GnRH-like_Lottia FC796606

MMPVPLKYFGLALTLALVTELAVGQHYHFSNGWKSGRKRSGGVSNLCEMRPELINYINTLLSEELNRIKNTCNLNTEDKDSDVDFSEGAFSRLQNGLKLAADRKWKK&CQDIKNLMNKTKPTN

>GnRH1_mus_gi|109733046|gb|AAI16900.1| Gnrh1 protein [Mus musculus]

MSLRVDMILKLMAGILLLTVCLEGCSSQHWSYGLRPGGKRNTEHLVESFQEMGKEVDQMAEPQHFECTVH

WPRSPLRDLRGALESLIEEEARQKKM

>GnRH_chicken-II-type_gi|89274017|dbj|BAE80724.1| chicken-II-type gonadotropin-releasing hormone [Gallus gallus]

MAPGGCLLLALLLLAGTAQGQHWSHGWYPGGKRDLSAPQAIPAWSPWLVPSRGRKSRYDPPAKSKASRLKVPPPVDPAELYVVTERYRQHRLVLSALRSIFRSEVVQRKREAQRDAEDSAALSEEHRLLMAWNDAENARQRARR

>GPA2_P._fucata_pfu_aug1.0_9176.1_53302.t1

MTNCYVRDYRFQASKPFLTKSGYLLPCSDVITVKSCWGRCDSSEIADYKIPFKISNHPVCTYSGAKKRIVRLQNCHPLHPDPYYVIYDALECACTTCNPEYISCENLNG

>GPA2_C.gigas_EKC24490.1

MWKRCVWLVVLAAFSAVCAVDLDSVTACLVREYNLFAQKPHVTPTGDVLECSGFVKVNSCWGRCDSSEIADYKIPFKISNHPVCTYSRVQKRRVRLPNCHPEHPDPYYVVYDALACSCRYCNSKYTSCETLNG

>GPA2_A.californica_EST NP_001191597.1

MSCLLARPSSCHLVDPRTTLSCHVRSYQFRVTKPPVISEDGQILRCSGIVTVNSCWGRCDSSEIGDYLMPYRISHHPVCTYTGRVPRQVTLSGCEDYPDPTFEVFDAAGCECRLCDSDYTSCENLNG

>GPA2_L.gigantea_EST FC741802.1

MRGWIREKNKLLIIIVLLLTVISTIENTPNFAARIGLVCRKRAYVLNESRPWIDMHGIRHPCVGTFRVWSCWGFCDSFEIGDYKMPFKISKPPVCTYFSRRSRFVRLNCPGPPNPEVEFFDATGCKCRRGNSEFESWENLRG

>GPB5_pfu_aug1.0_9176.1_53302.t1

MAITSLVIVTLILCYHGSLISAALNLEEMTNCYVRDYRFQASKPFLTKSGYLLPCSDVITVKSCWGRCDSSEIADYKIPFKISNHPVCTYSGAKKRIVRLQNCHPLHPDPYYVIYDALECACTTCNPEYISCENLNG

>GPB5_Cg_gi|405958354|gb|EKC24490.1| Glycoprotein hormone beta-5 [Crassostrea gigas]

MWKRCVWLVVLAAFSAVCAVDLDSVTACLVREYNLFAQKPHVTPTGDVLECSGFVKVNSCWGRCDSSEIADYKIPFKISNHPVCTYSRVQKRRVRLPNCHPEHPDPYYVVYDALACSCRYCNSKYTSCETLNG

>GPB5_Aplysia californica_NP_001191597 glycoprotein hormone beta subunit

MSCLLARPSSCHLVDPRTTLSCHVRSYQFRVTKPPVISEDGQILRCSGIVTVNSCWGRCDSSEIGDYLMPYRISHHPVCTYTGRVPRQVTLSGCEDYPDPTFEVFDAAGCECRLCDSDYTSCENLNG

>GPB5_Lottia gigantea_FC741803

MRGWIREKNKLLIIIVLLLTVISTIENTPNYAARIGLVCRKRAYVLNESRPWIDMHGIRHPCVGTFRVWSCWGFCDSSEIGDYKMPYKISKHPVCTYSSRRSRIVRLNCPGHPNPEVELFDATGCKCRRCNSEFESCENLSG

>LASGLVamide_pfu_aug1.0_2190.1_44266.t1

MDPLASGLVGKRMMDPLASGLVGKRMMDPLASGLVGKRMMDPLASGLVGKRAFDDLASGLVGKRNDQYVKDVESGKGLSRHMADKRLMDDLASGLVGKRSTGWINPAQRQNLFADTSDSHMEKRSLSNGPFRRFPMYNSFDKRPFDFLASGIIG

>LASGLVamide_C.gigas_EST CU986442.1

MATTKSNAVLFTTIIYVVYTIGPIASQKSFRYDENLYQDQDETLVEKRQFDRLASGLIGKRRLDSVASGLVGKRRLDTIASGLVGKRRLDSIASGLVGKRRLDSIASGLVGKRRLDSIASGLVGKRRLDSIASGLVGKRRLDSIADGLVGKRKMDYHSNTYPDFPAAEKRMMDSLASGLVGKRMMDSLASGLVGKRTMDSLASGLVGKRTLDSLASGLVGERQSFPDFSKGGNEKTENTGQAFGSKTFSEFQLSKRNFLNHLTIKQTFLNFEQYFSFLA

>LASGLV_L.gigantea_LASGL1

MMYNIVCLAFLCLVTDKYCVCLVRVAKRSIENFDEEDADFIPASYPYITKRLYHGLSFYPFQHKRPFDYLASGLIGKRAYGRFGDYRDFMWKRPFDTLAAGLIGKRSFETTQPKFLSKRSIRGMYLDKLNMQNINEGSMASPVVTKRNIDSLANGLVGKRPMDSLASGLVGKRPMDSLASGLVGKRHMDSLASGLVGKRHMDSLASGLVGKRYMDSLASGLVGKRHMDSLASGLVGKRHMDSLASGLVGKRHMDSLASGLVGKRHMDSLASGLVGKRHMDSLASGLVGKRHMDSLASGLVGKRPFDTLASGLVGKRPFDTLASGLVGKRPFDTLASGLVGKRPFDTLASGLVGKRPFDTLASGLVGKRPFDTLASGLVGKRPFDTLASGLVGKRPFDTLASGLVGKRPFDTLASGLVGKRQMDSLASGLVGKRLVDSLASGLVGKRPMDSLASGLVGKRHSLNDSSSQRGTDSELKEQVGKGNSDETDNDDISEQFLNEQISSDKQNPEN

>LASGLVamide_L.stagnalis_ES574531

MDTPLAVFFSLVIMISLTVSTDSASNNLDKRSNNRHRYQPRVSRPYSEYRGGFVWKRPFDEIGSGLIGKRSFDDIGSGLIGKRPFDEIGSGLIGKRPFDEIGSGLIGKRPFDEIGSRLIGKGHLMKLEV

>LASGLVamide_Lottia_LASGL2 FC774474

MTYLSGIMMYNIVCLAFLCLVTDKYCVCLRVAKRSIENFDEEDADFIPASYPYITKRLYHGLSFYPFQHKRPFDYLASGLIGKRAYGRFGDYRDFMWKRPFDTLAAGLIGKRTIDSLASGLIGKRSFETTQPKFLSKRSIRGMYLDKLNMQNMNEGSMASPVVTKRNIDSLANGLVGKRPMDSLASGLVGKRPMDSLASGLVGKRHMDSLASGLVGKRHMDSLASGLVGKRYMDSLASGLVGKRHMD

>LASGLVamide_Aplysia_gi|7327284|gb|AAB27696.2| LASGL [Aplysia californica]

MAHHRGHRHILLYVSLALSLGLALAEDATDPSDDTGSFDDVEAVSEEADLDPYSMSQELNKRPNVDPYSYLPSVGKRAFDHYGFTGGLGKRKIDHFGFVGGLGKRQIDPLGFSGGIGKRYDSFAYSAGLGKRGMDSLAFSGGLGKRGMDSLAFSGGLGKRGMDSLAFSGGLGKRGMDSLAFSGGLGKRGMDSLAFSGGLGKRGMDSLAFSGGLGKRGMDSFTFAPGLGKRGMDSLAFAGGLGKRMDGFAFAPGLGKRMDSFAFAPGLGKRGMDSLAFAGGLGKRMDSFAFAPGLGKRMDSFAFAPGLGKRGLDRYGFVGGLGKRGMDHFAFTGGLGKRDSGEASGDLEEGKRGLDAYSFTGALGKRGLDRYGFVGGLGKRGMDDFAFSPGLGKKRMDSFMFGSRLGKRGMDRFSFSGHLGKRKMDQFSFGPGLGKRGFDHYGFTGGIGKRGFDHYGFTGGIGKRQLDPMLFSGRLGKRSSSEQEEEDVRQVEKRSTTEEQSSKSL

>LFRFamide_pfu_aug1.0_26025.1_55118.t1

MFNVFKFISIILEQINLQHGPDMHVSALADVSSSERILQKRSDPNDDISDSYEFTDADLDELVKRGSLLRFGKRGSLLRFGKRGSLLRFGKRGSLFRFGKRYDEHVEKSYPYTDDDSSWMDSTGSKRGRSLFRFGKKRSSLFRFGRSADKAPHTPFRFGREEVY

>LFRFamide_Crassostrea gigas_FQ665026 mRNA sequence

METKTLLIWVFAAFLAFIEVSTASKESKLNHEESEVKSLKRRSNPMDDVTDTYDFSDEDLNELMKRGSLFRFGKRGALFRFGKRSEEDKRGSLFRFGKRMDGYYVSPYASSENFPEKDDKRGSLFRFGKRSSLFRFGRSVDNEKPHTPFRFGREEEDEI

>LFRFamide_Argopecten irradians_Bay scallop_CB415749 mRNA sequence

ARGICGAALFLAIIDLAFTDDGKLVDSDQSEIKKRMAPDIEYEGYNPYSDLSDEDLAEIIKRGSLLRFGKRGSLLRFGKRGSLLRFGKRRSILRYGKRFDDQDENMELPLEDYADAGSDDKRGTLFRFGKRRALFRYGRSADKPHTPFRFGREEEYLEK

>LFRFamide_Idiosepius paradoxus_DB917861 mRNA sequence

MEIKVMSLLATVFTVFLVQINCEDLHKIQTDPSGIGNIIGLPDGDDSELSRSPIIDESALGIDDMDKRNSLFRFGKRGNLFRFGKRGNLFRFGKRGNLFRFGRAGNKDDPENEGLKRTIFRFGKRDGGLEDLYDYEDPTSQQVVAAPSPGAGDKRGSFFRYGRSRTFFRYGRSTDKNAEKRPHTPFRFGREEE

>LFRFamide_Haliotis diversicolor GT867779

MDSSQILSILMLVFAMVLTGMALGTKEEQDSEVAVAAPSEHHKRSVDFPLSQDLLDDDAYDMDKRGSLFRFGKRGGLFRFGKRGTLFRFGKRGSTLFRFGRGGSDDLWMPVNEEEQAAKRNFHWGRETEE

>LFRFamide_Aplysia californica_ACJ76422 LFRFamide precursor

MEPRPITLSLAVLLAALCTTCATGSAKLVSSSEDNTNSHTDGLTRDHQPQKRSAAAILSPADDALLVRADEGEEEGVGGSYPGIPLSLPGTADKRSTLFRFGKRGAGTLFRFGKRGSLFRFGKRGGALFRFGKRGGALFRFGKRGVASGSGAFPLADDSDVKRTLFRFGKRSNLDLLRDVLSQYQLSAYPDAYPDAYPDAYIDDVDSKRGVDGFHWGDDQ

>LFRFamide_Lottia _154926

MEFKSIICILVGFLFIATVTCLKSNHLLSDDEPNSIRTVSVLDALGEADDDDVSANDGLLAISEWFPRVVFGPSYKPDKRGSLMRFGKKASMLRFGKRGSLFRFGRNNPLPLYLQAPIRSEREDIPKTKKVPDSFHWGREAE

>LFRYamide_pfu_aug1.0_53964.1_05542.t1

MDSVRFVLTFGLIIAVFTYTSFGGPVNCNHICMNAIASPYCNCEIRSPFRWGKRSIKIPFRFGKRNYQDSYLNTDSYYIRSRFFGKKVTLKFMLEQSSRHYALLRHERFHAKYKYVPSNSFISGNDTGCAHIFIQ

>LFRYamide_C._gigas scaffold895

MSAQRNLLLAVVGLCFCFQLLPVKALNCNDICMSGIASDSCNCNVKLPFRWGKRSTLRLPFRFGKRSSSSMESYGDDR

>LFRYamide_Haliotis diversicolor GT866929

MSRTILLFLSCVVLCCVFTSVSARYLDCTKECYYNRYYTLCDCPSPKMPLRWGKRGVRVPFRWGKRSFDNYQQGSYGDSWYDSAPLDLSAYFEDIA

>LFRYamide_Lottia FC573115

MPTKIAIISVCLVIAVVLDDVACRYLECPDGCFTTTDSPCVCPFSSLDKRGVRLPFRYGKRNSFLRYGKRNNFFRYGKRGSIFRYGKRDEDSLSDSSVESNNPYNILQSDYSTQ

>LRNFVamide_pfu_aug1.0_46.1_21939.t1

MGGTLKSICLKLFAKKTFISCLCVVLLHSFSVTAEVNEDSIREKRDADSPLYGNDETKQTNLNNENNRQTSMDDSGLPNDEFEKRMRYFVGKREDGPAFIDKRTRYFLGKRLMSEAELHKRLRYFLGKRADENDYYKRMRYFVGKRDGDSGYDTAKRMRYFVGKRTGDADDEVEKRMRYFVGKRMRYFVGKRDDEVNDTINDLPEKRMRYFVGKRGEAQDSLADIGENEISKRRRYFLGKRLRYFLG

>LRFNVamide_APLYCIA Q86MA7 PRQFV APLYSIA

MSSQLLICSVFVLFTFGPNSFPSCLAQEQAGNSDATQLSADAKAPESAKDKSGDVQNDGTKSVRSKRDLEIDFGSGDVQKRAREFVGKRAAPPVFQTPLVQDKISGFIPSETESPVIGEFAFPGSVFMDDEEALGAEEEPMDDEDLEFYKRPRQFVGKRGIDDYLLQEKLKDFIEKRPRQFVGKRPRQFVGKRPRQFVGKRPRQFVGKRPRQFVGKRPRQFVGKRPRQFVGKRPRQFVGKRPRQFVGKREADPSFLFEDKRVRDFVGKRSLDFLGGANWYNPYDVTMEPQSEGSDLQGFSKRPRQFVGKRDAFDMFEFSKRPRQFVGKRDQDEMFDFSKRPRQFVGKRDLQEFFDLSKRPRQFVGKREFDDEIDFSKRPRQFVGKRENDDDFDLSKRPRQFVGKRENDDEFDLSKRPRQFVGKRENDDELEFSKRPRQFVGKREDDEIDFSKRPRQFVGKRENDGEIDFSKRPRQFVGKRENDDEIDFSKRPRQFVGKREDGEIDFSKRPRQFVGKRENNDDLDFSKRPRQFVGKREVDDEIDFSKRPRQFVGKRENDDDLDFSKRPRQFVGKRENDDDLEFSKRPRQFVGKRENDPLLDFSKRPRQFVGKRENDDDLDFSKRPRQFVGKRENDPLIDFSKRPRQFVGKRESDGDFELSKRPRQFVGKRDVDGPGLSKRPRQFVGKREDYDIDFAKRPRQFVGKRGNEDEFEMSKRPRQFVGKRNFEELDQDFLRHMHDILDKRIPQFVSLPSLTAAKRVREFVGKRSDAAFLETLRHLRDYVGGQDEQNVSEFSYQHPYPSDLNDVGLIQQKRIREFVGKRGGDVDDINTTYRLGDFVSQPMSFVEEPSWLCRQLNAFGIS

>LRFNVamide_Lottia full sca_43:1667926-1670130

MWSQFLLFLTLTLIIQLKPVIAGDLNDLKETQGDNKNVKTIGVDEEEIKNIKRLRNFVGKREHVNDEDTSFKDRIGRIHSLDLVSSDKRLRNFLGKRSETDSFFQQKRLRNFLGKRLYTSNDKRLRNFVGKREDFTTEDKRLRNFVGKRNDLISEKKRLRNFVGKRDEFTSGEKRLRNFVGKRDEFTSEEKRLRNFVGKRDEFTSEEKRLRNFVGKRSDRDFSEENSLQKVDDNNQKSGQNQEKRLRNFLGKRNVFDVDVKRLRNFLGKRIFTDSSLHKRLRNFVGKRSDDILEKRLRNFLGKRYNVDSSEGPFSYQVKRLRNFLGKRDYFKYGDAVKRLRNFLGKRDNPFKLEYSNFKTGNDLADSFKTSSGAGPVSKRLRNFVGKREYFESKDKRLRNFVGKREKTHDNLNEEKRLRNFLGKRQGQSQSQLESDNITEQKRLGNFVGKKRDIRDSEDDNDEPMQKRLRNFVGKREEDGDIEEEKRLKNFVGIRDSNDDNAVEKRLRNFVGKRSAETVEKQDYPDTFSRKRRSLDDYEEFYNPSKRLRNFVGKRSHSDYFHDISEEKRLRNFVGKRQSSYFSYPEKRLRNFVGKRLRNFVGKRSEYDLNGDFTPWYLEEQKRLRNFVGKRDGPYLSYPKYPVQKRLRDFLGKRSSSLDDTEYSQDQTYGIDKRLRNFVAKRSPSEVYGTLYNEPSVHQPHKRFEEVIRKRVARAVSSQFVPVIHKRLREFIGK

>LRFNVamide_Crassostrea gigas CU990208

MRVPVFLYSFVVFLGVCCVDEVISTDKDIESSSQEIDHQRDFATLNKRRYGDDDFQKRLRYFIGKRSDTGDKEKRIRYLLGKRFAFDGNVNKRLRYFLGKRDFSDTKKRTRYFIEKRSDELGSHDLEQNMRHILEKQESDKVNPGLEKRMRYFLGKRMRYFLGKRANSATSNSDSHNESSKRTRYFLGKRAGTRYFLGKRFRYFLG

>LRFNVamide2_Crassostrea gigas2_EST CU988206.1

MKLTFGTILTLLWAFSSIKAKPAEEQTKAAGKADDRVKRSPFVPKFIGKRDDENDDALLSLEAAIRDELLSQEPFDIYPSDADDAFEREARMNRQLFVGKRDSNPLGKRTAPILGFKRFSPREELKRSTILVPEDISEFDMNKRGNLPMFVGKRRAPFFVGKRRMHLVVGRGGNMNNPKLVGKRGTPLFVGRRRADTDDKPIYYSYIALRRSVPDELRMRDSIADSLLNGDNFYSAADTGSGVLDIPDDEDSLSNSSSQKFKRFETPVFIGKRNSLIADLASAKQAHSQLLQKRFEPPMYVGRRSEQPTQAHRAQVQYQQGSIS

>LUQIN_C._gigas_EST CU97729

MIWVFCTPKEDQKGHLEPGIMLCHLEQLLLSCIVLCLVKVCASLKAQDEASVNDHDIVRRQAMGHSFGDELEGFDLDPKRVNNWNQFPAWGKRLSKRRWSSLGAWGKRSWLDRLISANNNWGKRWKSMSNSWGKRQAPSEFDGLSDDYINIKKRSVDSKFSHNSRNKRSIPTELSPEQNEEKRRWSSLSAWGKRSDDDEKRRWSSLSAWGKRSNPEAIDDNDSDNISKRKWSSFSSWGKRGDPVDLSKRLYSYWQNRLMTNNPWMERRGWNAFSSWGKRSMD

>LUQIN_A.californica_NP_001191480.1

MTLHLASPFILLFTIAYSLTSAVQGLEPLPAASLSDSPASGADVPPLPSSAATNAAVDKEWLRQKLEEGQFLPQQDKRWGGINSWMTHRLGGPSERDSSQDSLDKQLLVNNVQNYDDSSKRKWSKFSSWGKRDASEETPEGGEGEDGLGAVKKWKNMAVWGKRAEDGLDKRWKQMATWGKREDGDVLGLGTDKRWKQMASWGKRLDDSDRDKKWKQMSVWGKREDNGEPLDKKWKEMSVWGKRDTLDDPEKRWKQMAVWGKRQGLDDRNDKRWKQMATWGKRNSSENYDKRWKQMSVWGKRDGDGDLDKRWKQMSVWGKRDGDGDLDKRWKQMSVWGKRNGDGDLDKRWKQMSVWGKRDGDEDVEKRWKQMSVWGKRDGDADLDKRWKQMSVWGKRDEDGNLDKRWKQMSVWGKRDEDGNLDKRWKQMSVWGKRDGDDNLDKRWKQMSVWEREREREWV

>LUQIN_I.obsolete EST FK719020.1

MQRMMELMLVACVLVVMTSLLQPTAATPSWKPQGRFGKRGGPSDYLGIQKKPHGWRPQGRFGKRSLASSSSPSSSSFEQSIDAELLKSAAVSGAIEVPVELLSTGENGMFLRLGVKPCSITGMDGIPPCTGASEGASETF

>LUQIN_I.paradoxus EST DB915092.1

MKTSEIIISITALLLIALTIGDGAPAPKWRPQGRFGKRVSELNEDPLWVILSSENEKRDELLPYGVANKPVHFENLLCVPVGVKNAYKCTRSDS

>LUQIN_L.gigantea ESP02754.1

MKLSEMIMCIMAVLLVTITLGNGAPQWRPQGRFGKRVNPTLSLLLHGDQEKQFLNHEKPFISEDKPYIPEVTDEISVERLTSDARDNLSKFLHRLCSESGLENVPRCSWSRKTSERSSDPIRLI

>cardioexcitory peptide_Lymnaea_gi|4105585|gb|AAD02473.1| cardioexcitatory peptide precursor [Lymnaea stagnalis]

MKTSQIMISTCCVLSVIFSLCYATPHWRPQGRFGKRTTEDDNVPDFGISRLQDIPIELMFSKNELSRRNSKPRLCSTSGVVDTRPAMHKSMRCK

>Mytilus inhibitory peptides_P.fucata.pfu_aug1.0_10475.1_31931.t1

DIDPALDEFEGSEGEIYKRSHKPIYVGKKSYDEPKDDVSNIDGAYASDLDTLDNDLRYLIQHSDAFQRDTRYKPRIVGRSDENPMDGEWMEKRRSPLFVGKRRAPIFVGKRRMHLIVGRGLDRAPKFVGRRRSPLFVGRRSNGRNYRDPVFSYVVMRRSVSSSGNAAPFSQASSAQSLLLALDDQSLADKSRQGRYIHPTAQAQGHVAQFALPQHKRFVSPTFIGKRTDEPNNLDSYDPDNSGFDIDHQPMVVHKRFEVPMFIGKRYSDTIENRENWNAEHSGAINWACSKYEDFIVRVKRLASKLLVQGYLAQRLKSSMRKFFGRYGDIIKAYQISLSRMVSDILNFD

>Mytilus inhibitory peptides_C.gigas_EKC39623.1

MKLTFGTILTLLWAFSSIKAKPAEEQTKAAGKADDRVKRSPFVPKFIGKRDDENDDALLSLEAAIRDELLSQEPFDIYPSDADDAFEREARMNRQLFVGKRDSNPLGKRTAPILGFKRFSPREELKRSTILVPEDISEFDMNKRGNLPMFVGKRRAPFFVGKRRMHLVVGRGGNMNNPKLVGKRGTPLFVGRRRADTDDKPIYYSYIALRR

>Mytilus inhibitory peptides_A.californica_NP_001191614

MCTRPGLAALLVLMTSCASSFSRADTQSASAAALSAASADAQAARQQQEQHLVAQQQQQQQQQQQHSNNNEPQQRAPSLDPYYRSLLDGSQGGQLFAPAQPVSQPDLSPDFSNPMGSSLSQSGTPEDSDTKVDTRGAAPKFFGKKRGQAPRFFGKKRAMAPKFFGKKSSEFPTSNSEQLALDTRGSPRFFGKKSFPESNREQRGSPRFFGKKRFDENVDIDERAAPRFFGKKSSGESAGDSGYISVASRGSPRFFGKKQDDDIMIAARGSPRFFGKKRSDDNVALDLRGSPRFFGKRQSSDLDDEISVALRGSPRFFGKKRADDEDILLGERGSPRFFGKKRANDENISFSLRGSPRFFGKKRSDESDDDNIGLVARGSPRFFGKKRSDETDDENIGLMARGSPRFFGRKRSDGLDDGGNIIDVATRGSPRFFGKKRSNSDSSDKSSDSALSSSESGRQTRQAPRFFGKRYVDEHHVSKRAAATAFPLIIEARQAPRFFGKREYRYPPRGSPHFIGKRFSLYRSPGKYSLSSPYMSAKEFKETFRRSDPFFMGKRTAELNEEGSDDFTNDDTDDENEYDETVLFKRGAPRFVGKRGAPRFLGRRGAPRFIGRRGAPRFVGKRGPPRFIGKRDLDWYQKALCAEADILELDDCADFLGNDDVKRQAPRFIGRKRGEDVSERDYAQLLEALSRLQAIKQIKARIQNEKRLWVPGMVGRRSEYNLGPFDEFVDESMER

>Mytilus inhibitory peptides_A.kurodai_BAC41320.1

MCSRPGLAALLVLVMACASSLSRADTQSAGVAALSPASADARAAPQQQEQHLVTQQQQQLQQQQHSNNNEPQQRTPSLDPYYRSLLDGSQGSQLFAPAQPVSQPDLSPDFSKPGGSSLSQSGTPADSDISVDTRGGAPKFFGKKRGQAPRFLGKKRGMAPKFFGKKSSEFPESNADDLVLDTRGSPRFFGKKSLTESDREQRGSPRFFGKKSSDEGGRSFENVNLAERAAPRFFGKKASGDDDYISLASRGSPRFFGKKRDEGIFPDDIMLATRGSPRFFGKKRSDDNVDSISFDLRGSPRFFGKRQPGGLDDDIAVALRGSPRFFGKKRSDDEDILLGVRGSPRFFGKKRSETENISLSLRGSPRFFGKKRSEESDDDNIRLVARGSPRFFGKKRSDGLEDNGNSIDVMTRGSPRFFGKKRSDSDISEESSDSAVSLSSSESGKQTRQAPKFFGKRYSDEHHVSKRAAATAFPYIIEARQAPRFVGKREYRYPPRGSPLFIGKRFGLYRSPGKYSLSSPYMSAKEFKETFRRSDPFFMGKRTAELNEVRSDDFADDDADDEDEYDETVLFKRGAPRFVGKRGAPRFLGRRGAPRFIGRRGAPRFVGKRGPPRFIGKRDLDWYQKAMCEEADILGLDDCEDFVENDDVKRQAPKFIGKKRSDDISDRDYAQLLEALSRLQAIKQIKARIQNDKRLWVPGMVGRRSEYNMEPFDEYVDESMER

>Mytilus inhibitory peptides__L.gigantea_ESO85156.1

MKSILVTSVVVVAGTLLTLVQALGADKFAEPATQKHSKIPTAALSHSGDTLLNTDAHQTNPSNIWKSKSKFDLDALPVVNRQDVQSHYAQRRSYDTPMFLGKRDLKNLSLRFKRATDLEDATEFSDDFDKRGVDIPQFIGRRRYDTPQFIGRRGYGVGVPAFVGKRAFRAPAFIGKKAFDAPSFVGKRKLTAPSFIGKRGLDVPAFVGRRAYAPPQFIGRRGMDVPAFVGKREFQTPMFLGKRAFSAPSFVGKRGLDAPTFIGKRKEESFRDLLATLQAYREYRRLMQTDKRFDAPLFVGKRSDYENDNLAAETLSRRFQCIPQWVFRMRLNVVDLNMSFIGGYKQNYFSLNILDDVQTKILLPLDDLHKVDSSKMDVPTVQLWDADMPRPNGARFLKLDPAHALGFLGQEASPVSKRYLEFVGKRDNDKRYAEFVGKRAGFGEKRYSRGSLSEYLSKRLRIRRPEFVGKRSNQKRYLEFIGR

>Myomodulin_P.fucata_scaffold1943.1 : 19966-31846

WIILLYNTGAEDEVLETNRQNAPLSRVRRGGLSMLRLGRGLQMLRLGKRAMPMLRLGRGASDSITQEQIRYIISLLLQEENYDNEPFRRQIPFPRYGKDLSSMQELIEELQRMPSQERRTDMYDLDEDSPRQIRPGPRPGRFRRSTAHMLQPDVSQEEAEVNSVERAPPLPRIGREQEDGEGEEGDIKVPMVKGNSDYYIDVAKDGSYYVADKRGMPMLRLGRGMPMLRLGKRPFKMLRLGRGSEGSDDEKRGMPMLRLGKRPFKMLRLGKRLESESESDKRAMAMLRLGRNSX

>Myomodulin_Aplysia californica NP_001191658 myomodulin neuropeptides

MWKILETCSCFLVVAVLSGLGKAQPESFSGSAVTDDSTSGANKRGWSMLRLGRGLQMLRLGKRGGSLDALRSGHQVPMLRAGRGSPDTSGRLDANELYAVLSAILDEPRDQSRRQPPLPRYGRDNNGVARDLLDALASDGESSSNFDLLSSLNNGPSYFRPAPRGGRYKRSLPDAGPADYPSLEDYLVQSRQFARPYSSRAVALPRIGRFSGSPRLQAKAVPRPRIGRQESQMREAKSAE

>Myomodulin_Aplysia californic_NP_001191423.1| myomodulin

MQVYMLLPLAVFASLTYQGACEETAAAQTSSDASTSSASSEHAENELSRAKRGSYRMMRLGRGLHMLRLGKRGGPVEPESEENLETLLNLLQGYYSDVPEYPSEFDDTDLAYPYEEYDAPAHPRYRRSTPPTDGVVAPDVLQKGSSEFEDFGDSQLDESDEGYYGYDPENYLYGDFEDYLEPEEGGLGEEKRSLSMLRLGKRGLSMLRLGKREGEEGDEMDKKQDESLNDDFENDDIKRTLSMLRLGKRPMSMLRLGKRPMSMLRLGKRPMSMLRLGKRPMSMLRLGKRPMSMLRLGKRPMSMLRLGKRPMSMLRLGKRPMSMLRLGKRPMSMLRLGKRPMSMLRLGKRDDDEKEKKSLNMSRLGKRSTQ

>Pinctada maxima GT282875

MMKYLVPLILLLHCYHKVTGAEDEVLETNRQNAPLSRVRRGGLSMLRLGRGLQMLRLGKRAMPMLRLGRGATDSYTPEEIRYIISLLLREENYDNEPFRRQIPFPRYGKDLEWQTLLEELQNQMPSQERRTDIYDLDEDSPRQIRPGPRPGRF RSTPMLHPDISQYEKEKRK

>Myomodulin_Mytilus galloprovincialis_FL499954

MKYSFTLLVVLYSYVVAGSSKVTESSHDDGQLNRVRRGGLSMLRLGRGLQMLRLGKRAMPMLRLGRGVESYTPEEIRVIINTLIGEERGDRQVPLPRYGKDVEVQMLLQRLLGDPQFQERRSMSLYDADDDSPRLINPGPRPGKYRYHRSLPDVPPQNVYDEEKFEDIN

>Myomodulin_Lottia_Myomodulin 1_FL499954

MFSVNFPLLLVSCLFLADLVYSEEEVSDTKTHDDSLSRTKRAGLNMLRLGRGLQMLRMGKRADFPMLRMGRSEPQYTTDEDDAIRQLIIDILTQQAADRDYFYPESYPYPPPQPRYRRSVSEFPQKEIDMQDLQKITEGGEEEMKQQEAENYPEIEGYPVFADDVEYKNEDEKRPMNMLRLGKRPMNMLRLGKRPMNMLRLGKRPMNMLRLGKRPMNMLRLGKRPMNMLRLGKRPMNMLRLGKRPMNMLRLGKRPMNMLRLGKRPMNMLRLGKRPMNMLRLGKKSDEDHSIETRKINMLRLGRSD

>Myomodulin_Lottia_Myomodulin 2 FC775836

MNGLSHIIPVFLLNLGLTCLLCNGAPTEDSLSRVRRGGWSMLRLGRGLQMLRLGKRTHPSGLDIYLDRDLHERQVPLPRYGKDLDWQNFLERHMLGNTEMDEEKRESNLPLTDPFLSEAIDGYPHMRPAPRGGRFKRSAGRFRYYPEVRSEERAVALPRFGRLIEKELKHKDGNDESKERAVPAPRFGRNPH

>Myomodulin_B.glabrata_ES751452

MDLFSSSAASVKSAGRFPRRSIFIGRFPRRSIFIGRFPRRSMLIGRFPRRSMFIGRFPRRSIFIGRFPRRSIFIGRFPRRSIFIGRFPRRSIFIGRLPRRSILSDLLSISSSNSSSKLASPLLPSLNILSCLLSLVKISSSVALLPSLSIFIDRFSNSSKLSASSPPSLGSSSSEPSGKT

>Myomodulin_H. asinina_GT275969

KRPMNMLRLGKRPMNMLRVGKRPMNMLRLGKRPMNMLRLGKRPMNMLRLGKREDETEGEEKRALGMLRLGKRSDEKVDGDAGVSTQQ

>Myomodulin_C.gigas_gi|405963046|gb|EKC28655.1| Myomodulin neuropeptide 2 [Crassostrea gigas]

MFLSLISGSADSIDESHSRVRRGGLSMLRLGRGLQMLRLGKRGMPMLRLGRSNGLSETDEDFMYPDESELDEGRRQVPLPRYGKDLQQQLQLEWLQSVLDSDLENNGVRIIRPAGRPGRFRRSLKEDNGEEKDSEERHIPHPRIGRLIQLDDLKYPTSNLGSYYLTDSNVYTDKRGMPMLRLGRGMPMLRLGKRLQADDSKRGMPMLRLGKRTNVQADSASQQETNQNKRGMPMLRLGRNAN

>Myomodulin_Pinctada_gi|262326550|gb|GT282875.1|GT282875 pmaximaP0021I07_718 Adult silver lipped oyster (Pinctada maxima)

MWICGFLCFVVVLNFRMMKYLVPLILLLHCYHKVTGAEDEVLETNRQNAPLSRVRRGGLSMLRLGRGLQMLRLGKRAMPMLRLGRGATDSYTPEEIRYIISLLLREENYDNEPFRRQIPFPRYGKDLEWQTLLEELQNQMPSQERRTDIYDLDEDSPRQIRPGPRPGRFXRSTPMLHPDISQYEKEKRK

>NdWFamide pfu_aug1.0_16542.1_32706.t1

MGNRCNVTKMFALDLQDKMRKVTVCLFLITMMITFNYSKAWYRGKRSNDADIESPYEPLENRHPLKREVDLSEKIGDDLKMDIIEEHD

>NdWFamide_L.gigantea EST FC678236.1

MKICLTLVVLMAVFVTSDAWWFGKRDLNGDAPPSAVKAALISLCSQYGDNTSSDPMAQDVRNVCQALKEASNNRRTIIS

>NdWFamide_A.californica_EB190874

MTSSIYGFITLSVVALISQTTCRSLDLLLDGDFNNGLASFDGSSKWSRLPLEFLAAFDLDPHQAQGQHLAEAPEAPLMEAMKRSRGPSPRRLRSYLRRAAGLRGMKRKMFWQPLGYMPASARAHNNVPEVVNENSQDSGTNVFRYG

>NKY_P.fucata pfu_aug1.0_3855.1_52103.t1

MKIPLLLSFSLCVTMTLGKSLSEAFRGEWKQDRPSRMLSIISNQERAMDLIRSLIRMTEDKNKRFYLEDEDYDPSLESEEDKMKNIVSEDVKSVSKRKVFWQPLGYVPASLRMTGGSREQSGESSNHAGGNILRYG

>NKY_C.gigas_scaffold43574

MPRYNNCSYVLCAITTIIFAIIATSESRDLSESDLMNFISTDQDRDFKVLSQLLKAEVLKRKLRDLSLDSNDIDLSDELMNELSSEKRRRQYLPSLLTVRKRKVFWQPMPYVPHNARHNSRDKDAGASNDIPNSAAILRYG

>NKY_I.paradoxus_DB914896.1

MTVNAFVHVMCMLALLVACAYSLPKRTDHASTLRFLQQNGLSDADSRALLHAYILGKLSGADGSFGKELETSEYPTIKRKAFWRPMGYLPFENHAGSGASSSNDNAAGGGSASAVFRYG

>NKY_R.decussatus_AM852749.1

MMSLCRVHVLFLFALTQLLCVSAFAAFVPDTNEPIDDITYDEISKSNTNQDEDGQFYKTLSKILASYLHRLEQRQVGEGYLSNTNMPYYKPSKRTSDVFTGLAKRKVFWQPLGYNPNGGSGENRGSSGSGRGPVFRYG

>NKY_H.diversicolor_GT868448.1

MQAYVIVAVLSALLTLCTCMTIDQSHAALSGVSRVKRQTLPDEYDDDDARMASLYNLLLSRIAELSAREAYPNTFADFKRTSSREINPDNSPITRKRKVFWQPLGYLPASARIGNQGGSSXAAKDKTGSSIFRYGK

>NKY_L.gigantea_Lotgi1 sca_10:2671719-2672204

MDIKIGETFWLWLLLAMFCTVYSRAASEYDLERPANSPYLDPESNIQPSFRYYLSLLSNSKPKFYPVDKILKRKMSWRPLGYLPAAVRMAGNDADKVSQATEDGSQMFRYG

>NKY_A.californica NP_001191477.1

MTSSIYGFITLSVVALISQTTCRSLDLLLDGDFNNGLASFDGSSKWSRLPLEFLAAFDLDPHQAQGQHLAEAPEAPLMEAMKRSRGPSPRRLRSYLRRAAGLRGMKRKMFWQPLGYMPASARAHNNVPEVVNENSQDSGTNVFRYG

>NPF_C.gigas_EST CU983945.1

MQTSSLLAVLLVTLLSVWTVLGNDSLLPPNRPSRFSSPGQLRQYLKALNDYYAIVGRPRFGKRDSEFSSYGQEPRSDGILSSSRDREDFAGPQWW

>NPF_A.californica_EST EB290938.1

MQRVILVVLLLSCMAVLSVRADNSEMLAPPPRPEEFTSAQQLRQYLAALNEYYSIMGRPRFGKRGDSFRKKKKVTDCAG

>NPF_A.kurodai_EST EY423701.1

MQKVILVAFLLCCMAVLSVRSDDSEMLAPPPRPEEFTSAQQLRQYLAALNEYYSIMGRPRFGKRGDAFRKREFFRTNGERYPDDAAAWTEFQ

>NPF_L.stagnalis_EST ES291525

MHKLLLVSLLVLSLAVMEVLCTEAMLTPPERPEEFKNPNELRKYLKALNEYYAIVGRPRFGKRNGARLSDMFRPSGDDFGDYSANWGDF

>NPF_L.gigantea_EST FC808552.1

MQKLVLSLLILAAVVMLEVTSQDSMLAPPDRPSEFRSPDELRRYLKALNEYYAIVGRPRFGRSVNKRSAADYLFKPSHKNE

>NPF_I.paradoxus_DB917827.1

MHKSSVVLLIVAMLFAVQVTCQGGMFAPPNRPAEFKSPEELRQYMKALNEYYAIVGRPRFGRSISSSRIDLSDLSGKSRKK

>NPY1_C.gigas_NPY1_OYG_10019288

MIQTVTRQIVVLGLLLTVTFSVTFSAPLDPDLESMALVPPSRPSGFQNFQEMQRYLMELNRFYNMMSRPRYGRDVHQRNRQKLKKISNLLHLASLSEM

>NPY2_C.gigas_NPY2_OYG_10007228

MLSSQICLLFIVTASWFCLNTASQLENMEMLAPPERPSSITNIDELRRYIKELNMYYLILARPRYGRSVSNKKPVRSQPQS

>NPY_L.gigantea_ESO85363.1

MQKLVLSLLILAAVVMLEVTSQDSMLAPPDRPSEFRSPDELRRYLKALNEYYAIVGRPRFGRSVNKRSAADYLFKPSHKNE

>NPY_I.paradoxus_EST DB919572.1

MQKTLLTTVVIAVVLIVQISSQEIMLSPPSRPAEVRNPNELREYMKALNEYYAIVGRPRFGRSLFSKKLGPSYLNEGLKAGENSE

>NPY_A.californica_NP_001191635

MQRVILVVLLLSCMAVLSVRADNSEMLAPPPRPEEFTSAQQLRQYLAALNEYYSIMGRPRFGKRGDSFRKREFFRTNGERYPEDAAAWTEFQ

>NPY_L.stagnalis_CAB63265.1

MHKLLLVSLLVLSLAVMEVLCTEAMLTPPQRPEEFKNPNELRKYLKALNEYYAIVGRPRFGKRNGARVSDMFRPSGDDFGDYSANWGDF

>Opioid_P.fucata_pfu_aug1.0_20062.1_47355.t1

MKFQWCSFAVICVIVSASLVLGDKESVIPVSDTGEGTRCDADMNTAQTCLACSRLPQDIAVQLTDCCTEDKAFQFCQICTKNSDECLKEAYSVEEANIGDISGPNDFDLGSNDYNKRFGTSFMGSSKFGKRFGRLFFGRSGGSSYIYGKRGKRYRKLGMGSGGFLFKRSDDGDYMDKRYGKLNMGGGSGFLYGKRDQDMDKRFGQGYDGKSSKYFFPDEDKEKRFGKLFMSGKRYGSLFMGRX

>Opioid_C._gigas_EST CU990987

MLMLVFLGMFCLSAADKENPIPVSDTGEGTRCDADLNIAQTCIACSRLPQIIAVKLTDCCSEDKAFQFCQICTKNSDDCLKEAFSINNLEKGVVLDGYSDVEDVPPEAIADNDMNKRFGTLSMGGSSGYLYGKRDLDKRYGRLGFGGSRGFIYGKRSDDKRFSRLGISGSSGYFFGKRDMGVDKRYKSLGMGGSTGFFYGKRSDDEKRFGTLGLGGSSGYLYGKRDSDKEKRFWRLGIKGSNEYLYGGKRKRFGQLGMDSSSGYFYG

>Opioid_L.gigantea_EST FC753982.1

MKNTIITMVSSLHFLVFVVSAIESKTQPETSTELDLCKANSDVNSVCQACSKLPYGMSMSVESCCHDYQILTMCDSCTSDPVRCMKDVLDLETNIVEQKTNPEVSNEDTDDVDDDESLEVKKRYGRMFFGRRPILYSNYLNKRFGRLFFGGRRQLFTPIKTYKQKRYGRLFMNKKSEGSNGAIEEVPDKRYGRLFMTPYLDKRYGAFRIGKPSSARYFWK

>Opioid_P.dumerilii_EST JZ398989.1

MCDSNKDINAVCKICDVLNGPQKGNCCSDPDALLWCHLTVDEVMRTEGGQGEESAEDSGEGDHSEDSEESNEMDKRYGSLFSNWRWYSHRNDRSANANAEEAKRGVEKRYGNLLGGLFSNSNYGKDKRYGDLFSNSNYGKDKRYGGLFSNSHYGKDKRYGGSLFGRLFSNKGKRYGNLLGHVFGRSVDDKRDGQAEKRYGSLFTPMFGGKKDKRYGSLFTPMFGGKDKRYGSLFTPMFGGKG

>Pedal peptide 1_pfu_aug1.0_7601.1_02469.t1

GFNKRPFDSITYGSIGGFKKRTFDPISYYGSMGRFRKRTVDPVSHKSSLGNLNKRRFDSIAYLSSFGGISKRRFDSIAYSSGFGNLKKRPFDSINYASSFGGFG

>Buccalin_C.gigas_gi|405950613|gb|EKC18589.1| Buccalin [Crassostrea gigas]

MSPMKAPVVSLAALIILLISQALAEDDQKIKGELCIPLRKYDFKLAVCFDSDTTRFTKTIESAIDNGPEIEGIPGYLKLIKNDNIMPADKRRFDSIAYGSSFDRLKNKKSVDSSGSERYLFSGVQKRFPFDSIAYRPGGFVKFTSKRIPYLYGNFKYGTHGLFAKKKTFDSIAHDFGSFGGFSKRTPDSTYSQIDDFGGLAKKSFDSIANSAGTFRRFSKRSVHSKRLGESKLISRRKRSFDSISHQSGRFGLFSKRRSFDSIANLSGYFGGFSKRYGLDSIAHGPGSFGGFSKKYGLDSIAHGPGSFGGFSKRYGLDSIAHGPGSFGGFSKKYVMDSIAHGPGSFGGFSKRYGLDSIAHGPGSFGGFSKKYIMDSIAHGPGSFGGFSKRRSLDSIAHGPGSFGGFSKKHSLDSIAHGPGSFGGFSKKRALDSIAHGPGSFGGFSKKRGLDSISHGSGGFGGFSKRFDSIGNKSGMFSRFSKKSADDKEYVKNSAD

>Pedal Peptide2_C.gigas_PP2 EST CU996563

MFTHHKTLFSLMAVLLLSIAAAKSSPAPTDYNRSDTEDIETKEQRSGEETDESTELFDEKQLGSMGTQEYSKKSMDSINGARGFRGFAKKHYDSIGGSRGLMGFNKKNYDSIGGSRGLQGFNKRYYDSIGGSRGLQGFNKKNYDSIGGSRGLKGFNKRYDSIGGSRGLKGFNKRYDSIGGSRGLQGFNKKNYDSIGGSRGFQGFNKKNYDSISGSRGLKGFNKRYDSIGGSRGLKGFNKKNYDSIGGSRGLQGFNKKNYDSISGSRGLQGFNKKNYDSIGGSRGLQGFNKRYYDSIGGSRGLQGFNKKNYDSIGGSRGLKGFNKRYDSIGGSRGLKGFNKRYDSIGGSRGLQGFNKKNYDSIGGSRGFQGFNKKNYDSISGSRGLKGFNKRYDSIGGSRGLKGFNKKNYDSIGGSRGLQGFNKKNYDSISGSRGLQGFNKKNYDSIGGSRGLQGFNKKSYDSISGSRGLQGFTKKGYDSISGSRGMKGFNKRHYDSINGSKGLSGFVKRSYDPISGSHGFSGFVKRETNTNGSKNSESFPEDALPEPTIDE

>Pedal Peptide3_C.gigas_PP3 EST AM854447

MWKLSFGAIKMQSLLNVLLVTLTISCCINRATGTADEERSSRHKRMLDRLGSGFIKREPDSKIDDLVPKLRYYLAKAISEQDDDSSPTEVDKRRLDRLDMGFIKKRRLDRLSTGFIKRDDDTEYENENEDGEDQMDKRYLDRLDSGFLKRFVPGEDKRYFDRLGSGFIRRSNFDRIGSGFIKRRFDRLGSGFIKRRFDRLNSGLIKRNLDRLNSGFIKRQRLDRLGSGFIKREEMDKRRLDRLGSGFL

>Pedal Peptide_A.californica NP_001191625

MRTAAICVVLLLCTARSQCSDQQSQNEGESQPANEEKRKFDKISSLSGGFGGFGKRDGEGGSTEETGDVEGKNSEQTTEEASEAGDVTDEANSSMAKRGFDSISSVSGFGGFGKKSGEKRRFDSINSSAFSGFGKRPFDGIDSVSAFSQFGKRPFDGIDSVSGFSQFGKRRLDSIAGSSGFSNFGKRRFDSIANNNAFSDFGKRRLDSIAGSSGFSNFGKRRFDSTANNNAFSDFGKRRLDSIAGSSGFSNFGKRRFDSIANNNAFSDFGKRRLDSIAGSSGFSNFGKRRFDSIANNNAFRIWQKAI

>Pedal Peptide_H.lucorum_AAB51694

MELSRIVALAFVVLCVCAANEETNEIIANEDSRAEKRSNVLLEKIDDNSEIHASELDKLTSDSEIEAEDVEDVDKRPFDSISGSHGLSGFAKRPFDSISGSHGLSGFAKRPFDSISGSHGLSGFAKRPFDSISGSHGLSGFAKRPFDSISGSHGLSGFAKRPFDSISGSHGMSGFAKRPFDSISGSHGLSGFAKRPFDSISGSHGLSGFAKRPFDSISGSHGLSGFAKRPFDSISGSHGLSGFAKRPFDSISGSHGLSGFAKRPFDSISGSHGLSGFAKRPFDSISGSHGLSGFAKRPFDSISGSHGLSGFAKRPFDSISGSHGLSGFAKRPFDSISGSHGLSGFAKRPFDSISGSHGLSGFAKRPFDSISGSHGMSGFAKRPFDSISGSHGLSGFAKRPFDSISGSHGMSGFAKREDALEDDRAFTITGEEEDDVLENLK

>Pedal Peptide_L.stagnalis_AAP57098.1

MGLVKFTLISVFILCVYAANEEVRESVSETVENSEADFEEENDLDKRPFDSISGSHGLSGFAKRPFDSISGSHGLSGFAKRPFDSISGSHGLSGFVKRPFDSISGSHGLSGFAKRPFDSISGSHGLSGFVKRPFDSISGSHGLSGFAKRPFDSISGSHGLSGFVKRPFDSISGSHGLSGFAKRPFDSISGSHGLSGFAKRSFDSTELSGISKIPVEPLFEDYEWNGENEWDSDQDAYPYEASVGKRPFDSISGSHGLSGFAKRPFDSISGSHGLSGFAKRPFDSISGSHGLSGFAKRPFDSISGSHGLSGFAKRPFDSISGSHGLSGFAKRPFDSISGSHGLSGFAKRPFDSISGSHGLSGFAKKRSE

>PFGx8amide_P.fucata pfu_aug1.0_19111.1_40122.t1

MIPFYRVSSFILLISTFTVAQYDQYKTIDKDPSALESEETADDDANLADDKVKRPFGGIFGKPYNIGKRPFGSIFHQDYDVGKRPFGGSYSNPYKIGKRPFGGSYGNSFKVGKRPFGSSYSKSFMIGKRPFGGTYKSWFKMGKRPFGGSYRGSFKVGKRPFSTYGGSFGLX

>PFGx8amide_C.gigas_EST CU986338

MAGIFQGSLLLLLFYNVASTSIHVQLIPSDTPMAGNDGMYHVEAKRPFGGVYGRPFTMGRRSSDFGNSHLATKRPFGRVFGRSYKLGKRDFVPPESHITVKRPFGRIFGNYRTNVHASKQKQTKRPFGSVYRISYSKRPFGGVYGNSYKIGKRYVLPQDEHNEVKRPFGKVYGRSYKFGKRPFGQVYGSRYGKRSVGSFDDEAAIILENTSPSEHNGQKLFVKRPFGKIYGKRYDVDKRPFGQIYSPRRPFGKRPFPGSVDFPMLNKRPFGKMYGRSYRQKKSPYKRYTDGKGSFVNIPDSIVDEPDSLVLPGPSDRISNQPYPAFFEDPGQKDAVFVEAGGYDVNVPGFEGQQRIRIWEKYTQT

>PFGx8amide_L.gigantea_EST ESO91628.1

MEYSVRYIMFILSCVILFCESTASFEEAVPSNYREAGNEVYNVQPPDTTDVDKRPFGRLFGPTWSMSKRPFGSLFGSYPSIRQTPVYRTVVEKRPFGRMFGDSFNTGKRSEMAKRPFGRMFGDSFNAGKRSEMTKRPFGRMFGDSFNAGKRSEMAKRPFGRMFGDSFNTGKRSEMAKRPFGRMFGDSFNAGKRSEITKRPFGRMFGPTFFDGKRSEVSKRPFGRMFGPGAFGKRYFEDDLPEDGTEYLEAPIEDLEKRFSWTLSGYKLGRK

>PFGx8amide_L.stagnalis_EST AAP57098.1

MGLVKFTLISVFILCVYAANEEVRESVSETVENSEADFEEENDLDKRPFDSISGSHGLSGFAKRPFDSISGSHGLSGFAKRPFDSISGSHGLSGFVKRPFDSISGSHGLSGFAKRPFDSISGSHGLSGFVKRPFDSISGSHGLSGFAKRPFDSISGSHGLSGFVKRPFDSISGSHGLSGFAKRPFDSISGSHGLSGFAKRSFDSTELSGISKIPVEPLFEDYEWNGENEWDSDQDAYPYEASVGKRPFDSISGSHGLSGFAKRPFDSISGSHGLSGFAKRPFDSISGSHGLSGFAKRPFDSISGSHGLSGFAKRPFDSISGSHGLSGFAKRPFDSISGSHGLSGFAKRPFDSISGSHGLSGFAKKRSE

>PKYMDT_pfu_aug1.0_32039.1_26587.t1

MQVKFEYSFSLQTDHAVDKRPKYMDTRDILDPFVQLTWISLQQLAAEGKINPELLNVVGDDEEKVGKIDRTNNTVDKRGHLRICLRRFGSHFKPYPCFRRENNLSSFREKSVNEYTFRYIEMSFPLP

>whitnin_Aplysia californica_NP_001191586 whitnin

MELQACNIFALFVVVVTLSVASSLPASRTDDVLQEASGLALNKRPKYMDTRRDLDVFKDLVLISIQELVDENRLNPALLPEEDAPKPVEKRMRYMGICMKKQYNNFIPFPCLRSGR

>SPTR_Lymnaea stagnalis_AAF36485 SPTR prohormone

MELPTYHIFALFVAAVALSVVTGSPTRTDEVLQEASGLALDKRPKYMDTRRDMDVFKDLVLMSIQELVDEERLNSAVLPEDDAPKPVEKRERYMGICMRKQYNNFVPVPCLRSGR

>PKYMDT_C.gigas_gi|405971647|gb|EKC36472.1| hypothetical protein CGI_10027007 PKYMDT[Crassostrea gigas]

MEISMPMLLCSSLVLVLISSVCSVPIDVARETENDHHLEEKRPKYMDTRDLYAFEKLVYLALKGLVEEGKVNPEVMTSEANEGFLDNQGENGESKEKAGPADKRGHLRICVRRSGSRYVPYPCFRS

>PKYMDT_Idiosepius paradoxus_DB918443 cDNA

QISSFTTRHETDRRTSNSYQDKNIMDSRLLAFVSVCLFLLTSPVFSAPAADPKPHLEQSKDLPISKRPKYMDTRDPQDIFKDLVYLTLQQLVSDGKVNPEVITGSDNGVPNKRGYQGLCLRRTANQRYIAYPCWRSGSK

>PKYMDT_Haliotis asinina_gGT276859

MDVTTTTVLGSLLLALLAVATSSGLPADEGRLDEASLAAEKRPKYMDTRQEDFEMVKQTVLIAMEELVQEGKLNPSVLAVGQGKPVEKRMYMGICMRQSHNHFIPYPCMRSGRK

>PKYMDT_Lottia_PKYMDT_sca_107:575153-586297

MEMRSSTILSLLVVLLLAAPTFCLPPDQGSEIDDLDKRPKYMDTREELSVLKDMVYIVLQELAEDGKINPELFTIHDQKAVVKRMKYMGICMRRTKYNAVVPYPCLRSGR

>RxIamide_C.gigas_EST HS206944

MSYACVVNLWLVCVLINFSGSLCNSIIDDPVQSLQDHLRNDFQDDIPQTKRSVNSCRGHPNLIQCIRAYFSNRFRLRHRAVQDPVEYRNIGKRNQVATSFQDRREDQQESRVDNLHDVYDDLLKATSSDFQGSDDKRAAVDFRSIGRRSFGADFRNIGKRYLGADFRNIGKRMNAADFRQIGKRGYAVDFRNIGKRLGVDFRNIGKRTVLKSDNVYLLSLLCSDTSTMAQCADRIASNTYI

>sCAP-Pyrokinin_C._gigas FQ669931

MIVSGITSFMLLFVLLLQTDAKPTSALRQKRAPKYFYFPRMGRSAFYFPRMGRNQESADQEQSEEDIPCCNVGVSRAWGPEDPEPRRICPAAKCCGHLSSKVYVDSDLTVYEICVSDVAALPMEQKS

>sCAP-Pyrokinin_A._californica_XP_005109114.1

MMDLQGENYLAFPRMGRARPGYLAFPRMGRSQMKTETGTDCCGLGMKSEFVIGQEGKEELRHGACSSSVACCAGLREIVDQKQDGVFFSMCVPDFVASRSSEESSSEVLSKLKSLLQK

>sCAP-Pyrokinin_L.stagnalis_O97374.1

MEITLPRVSLSLAVLLVIVCSVDAQNYLAFPRMGRSGYLAFPRMGRSHFKSETSADVTGCCGVGIKNEFLIGQDGKEEIRSACGARADCCEGLKEVVDQKNDGVYFSMCVPDITFAQASSVRSSEVFNKLKSLLEK

>sCAP-Pyrokinin_L.gigantea_ESO91141.1

MEPSPVKIVACFVIFAVIGTEAMNYMAFPRLGRSGYLAFPRLGRSDGQTIQSSEFEQAECCPEGIKSHWIIIGASKPEIRSRCSPKSVCCEGLEELVGRSSDHIFYTKCTKITDEKTENTVLDRLLRKS

>sCAP-Pyrokinin__T.diomedea_ABU82756.1

MEMTMPRATVSLTLLFVIICTVDAMNYLAFPRMGRSGYLAFPRMGRSQAKAGTAEAIDTECCGIGLKSEFAVSDDGKEELHNICTASVSVCCEGLRELADEKPNGVVYSMCVPDVSKMYPSSYNKLKRLLTK

>Tachykinin_C.gigas_EST CU990972

MVYLQRALFSLVFLITYSVTSATTGQLWDSVESDLDDTIESGDPVHKPLINFLINPKDLHKAGIPESSSEEKRFGFAPMRGKRFNNDVEFPLVRNNKRARFFGLRGKRVPSLVQRLSDEDFKNILGKLLNDACDFSTNSNDADSSLEKRFRFTALRG

>Tachykinin_O.vulgaris_BAD26598.1

MYAIRVSSSRRISCFLQVSLLFSGLLNTLQWGAFASYLPKQLEPQQKNYELDSNNVNSSRYPGHEEIMEFLRKLGFLANTIESRASIDDTDSESAERMKKVNPYSFQGTRGKRLNANSFMGSRGKRVLVNDKRTVSANAFLGSRGKRLVTLLGEKPQLNDLTGVIDKKSDALAFVPTRGRSQSTNDELAYTGPMLLREGRRMNSLSFGPPKGKKYSPLDFIGSRGKKSTFEDYVLNTQNDSPKENYERRASLHNTFIPSRGKRST

>Tachykinin1_L.gigantea1_ESO86763.1

MNQDCGFYILLVSVFVLFSLQIVLADDTELYKNNEEKRFAAFNDFSNPETPYSKQYWFYPHTSQGFLKTLLRYLALNEQSYRNTVKRQRTFGFVGTRGKRGDTFDGLKDFVRKLQDEINRRKYGSMLLDNSLDNSQNGYNKLELKLDQ

>Tachykinin2_L.gigantea2_ESO99687.1

MWIYLATTSLFLLCKVCAINGAAIYDAGSINQNFQEGLNEPLETPLPRVPSPHIIDDAFSSLNKWRNSDDLSKQKILSSFLKEIPVLTNGYAINQYPLSKRHPNFGFMGSRGKRTQDDIGASDFLNTIRPGFRRALETLRSQQMQGLSPELSNSKRQPAFGFHAVRG

>Wx3Yamide_P.fucata_scaffold126921.1:1..1117

MVLRQIPSIWGNNSVFRKDSIIHIRFCMIDFQKCLFSGTLAYPLSSQNNKELQDARLLALIKGINSQSPSNDMDIKNDKRGQGWAIAYGKRGSQKWSMAYGKRNSIFDKERYSQSPRFYPTKRTFSTDQTYYTWGQKPIKRQQGWHIAYGKRSIFLPEREY

>Wx3Yamide_C.gigas_EST FQ667218

MELTGNCCIMSLTVVFMIAQGSSGFSISGSNELRKNADDTLLNLITGMYSAGSYKPDEESQKRGQGWAISYGKRASPGWSITYGKRDSMDEFQQFSAKSRPRYIITKRATPFGVSGFHWNPTPNKRQQGWHIAYGKRFATPLYRR

>PXP_Pfucata_pfu_aug1.0_10475.1_31931.t1 PXP partial length

DIDPALDEFEGSEGEIYKRSHKPIYVGKKSYDEPKDDVSNIDGAYASDLDTLDNDLRYLIQHSDAFQRDTRYKPRIVGRSDENPMDGEWMEKRRSPLFVGKRRAPIFVGKRRMHLIVGRGLDRAPKFVGRRRSPLFVGRRSNGRNYRDPVFSYVVMRRSVSSSGNAAPFSQASSAQSLLLALDDQSLADKSRQGRYIHPTAQAQGHVAQFALPQHKRFVSPTFIGKRTDEPNNLDSYDPDNSGFDIDHQPMVVHKRFEVPMFIGKRYSDTIENRENWNAEHSGAINWACSKYEDFIVRVKRLASKLLVQGYLAQRLKSSMRKFFGRYGDIIKAYQISLSRMVSDILNFD

>PXP_Aplysia californica_NP_001191614.1| MIP-related peptides precursor

MCTRPGLAALLVLMTSCASSFSRADTQSASAAALSAASADAQAARQQQEQHLVAQQQQQQQQQQQHSNNNEPQQRAPSLDPYYRSLLDGSQGGQLFAPAQPVSQPDLSPDFSNPMGSSLSQSGTPEDSDTKVDTRGAAPKFFGKKRGQAPRFFGKKRAMAPKFFGKKSSEFPTSNSEQLALDTRGSPRFFGKKSFPESNREQRGSPRFFGKKRFDENVDIDERAAPRFFGKKSSGESAGDSGYISVASRGSPRFFGKKQDDDIMIAARGSPRFFGKKRSDDNVALDLRGSPRFFGKRQSSDLDDEISVALRGSPRFFGKKRADDEDILLGERGSPRFFGKKRANDENISFSLRGSPRFFGKKRSDESDDDNIGLVARGSPRFFGKKRSDETDDENIGLMARGSPRFFGRKRSDGLDDGGNIIDVATRGSPRFFGKKRSNSDSSDKSSDSALSSSESGRQTRQAPRFFGKRYVDEHHVSKRAAATAFPLIIEARQAPRFFGKREYRYPPRGSPHFIGKRFSLYRSPGKYSLSSPYMSAKEFKETFRRSDPFFMGKRTAELNEEGSDDFTNDDTDDENEYDETVLFKRGAPRFVGKRGAPRFLGRRGAPRFIGRRGAPRFVGKRGPPRFIGKRDLDWYQKALCAEADILELDDCADFLGNDDVKRQAPRFIGRKRGEDVSERDYAQLLEALSRLQAIKQIKARIQNEKRLWVPGMVGRRSEYNLGPFDEFVDESMER

>PXP_Pomatoceros lamarckii ADB11404 FV-amide precursor protein

MTSQRFLFVLLYIAPLSFCEVMYPDLDPEEEAERNRVENLIEQYAMNELIADQLSAGLSASNDANEFQTADEHTNFQSLSKRPRFVGKRDQDEELDIGEFSKRRMFVGKRPMYVGKRNYFGDSVSSKRRMFVGKRDSMDSIDKRPRFVGKRFYMDDSDSLSNEVAKRPRFVGKRSYENDLLDFDKRRMFVGKKAILGQQYDKRPKFVGKRFMLYEDDLDKRSRNIFAKDFDKRPKFVGKRFEPSSDDDFNDLSFLYGEVEKRPRFVGKRRMFVGKRSEEDTSSFDKRRMFVGKRSAFDSTMTDDMPVEVNKNMFSDQSNEKRRMFVGKRDNEIADMDLFKNGGDLDSLVLNSNDKRSIDSPRDRLYTDDNLTTGLDKKAANRFVGKRSVNLKTAVEHARNKEGFLTAGRV

>MIP-related peptide precursor_Aplysia kurodai_BAC41320 MIP-related peptide precursor

MCSRPGLAALLVLVMACASSLSRADTQSAGVAALSPASADARAAPQQQEQHLVTQQQQQLQQQQHSNNNEPQQRTPSLDPYYRSLLDGSQGSQLFAPAQPVSQPDLSPDFSKPGGSSLSQSGTPADSDISVDTRGGAPKFFGKKRGQAPRFLGKKRGMAPKFFGKKSSEFPESNADDLVLDTRGSPRFFGKKSLTESDREQRGSPRFFGKKSSDEGGRSFENVNLAERAAPRFFGKKASGDDDYISLASRGSPRFFGKKRDEGIFPDDIMLATRGSPRFFGKKRSDDNVDSISFDLRGSPRFFGKRQPGGLDDDIAVALRGSPRFFGKKRSDDEDILLGVRGSPRFFGKKRSETENISLSLRGSPRFFGKKRSEESDDDNIRLVARGSPRFFGKKRSDGLEDNGNSIDVMTRGSPRFFGKKRSDSDISEESSDSAVSLSSSESGKQTRQAPKFFGKRYSDEHHVSKRAAATAFPYIIEARQAPRFVGKREYRYPPRGSPLFIGKRFGLYRSPGKYSLSSPYMSAKEFKETFRRSDPFFMGKRTAELNEVRSDDFADDDADDEDEYDETVLFKRGAPRFVGKRGAPRFLGRRGAPRFIGRRGAPRFVGKRGPPRFIGKRDLDWYQKAMCEEADILGLDDCEDFVENDDVKRQAPKFIGKKRSDDISDRDYAQLLEALSRLQAIKQIKARIQNDKRLWVPGMVGRRSEYNMEPFDEYVDESMER

>PXP_Lottia_PXP Vamide

MKSILVTSVVVVAGTLLTLVQALGADKFAEPATQKHSKIPTAALSHSGDTLLNTDAHQTNPSNIWKSKSKFDLDALPVVNRQDVQSHYAQRRSYDTPMFLGKRDLKNLSLRFKRATDLEDATEFSDDFDKRGVDIPQFIGRRRYDTPQFIGRRGYGVGVPAFVGKRAFRAPAFIGKKAFDAPSFVGKRKLTAPSFIGKRGLDVPAFVGRRAYAPPQFIGRRGMDVPAFVGKREFQTPMFLGKRAFSAPSFVGKRGLDAPTFIGKRKEESFRDLLATLQAYREYRRLMQTDKRFDAPLFVGKRSDYENDNLAAETLSRRFQCIPQWVFRMRLNVVDLNMSFIGGYKQNYFSLNILDDVQTKILLPLDDLHKVDSSKMDVPTVQLWDADMPRPNGARFLKLDPAHALGFLGQEASPVSKRYLEFVGKRDNDKRYAEFVGKRAGFGEKRYSRGSLSEYLSKRLRIRRPEFVGKRSNQKRYLEFIGR

>PXP_C.gigas_gi|405975026|gb|EKC39623.1| hypothetical protein CGI_10007081 PXP [Crassostrea gigas]

MKLTFGTILTLLWAFSSIKAKPAEEQTKAAGKADDRVKRSPFVPKFIGKRDDENDDALLSLEAAIRDELLSQEPFDIYPSDADDAFEREARMNRQLFVGKRDSNPLGKRTAPILGFKRFSPREELKRSTILVPEDISEFDMNKRGNLPMFVGKRRAPFFVGKRRMHLVVGRGGNMNNPKLVGKRGTPLFVGRRRADTDDKPIYYSYIALRRSVPDELRMRDSIADSLLNGDNFYSAADTGSGVLDIPDDEDSLSNSSSQKFKRFETPVFIGKRNSLIADLASAKQAHSQLLQKRFEPPMYVGRRSEQPTQAHRAQVQYQQGSIS

>PXP_Hasinina_gi|207118449|gb|GD272561.1|GD272561 4223 Haliotis asinina developmental microarray library Haliotis asinina

MCGMISSSFILLLGSVLTFAEDADRPDPKAFRPSSANLASALIQRNLDLHSFEGPSAGAENSQWDFQDTEPRDPRLLAAPQAFGFLGKPEGDAPLSKRYAEFLGKRVFDKRFSEFLGKRDMATSDLMRQNMDNRLSALLNKRLRYRIPEFVGKRARSQQSGFYEFAGK

>WWamide_pfu_aug1.0_5029.1_59683.t1

MKCLIHCAIVITTLTFSLCEIYDEHKEADELDNISPNLQKDLHLKSSLSDSRTADKRAWQTSSWGKRKWPGTNVWGKRKWMNVWGKRKWASMGTWGKRSDPLIDNGLENIHKRDTEHSFPLKRKWNQFVTWGKRSMPIQRKWASVSLWGKRSQDNYNEPEKRKWSALTSWGKRDDVGDINSDKNKRKWAMSAWGKRTNPIDGDNDMNKKWALASWGKRNDIYNTPSLQDNDAKSDNTVDKRKWSNFSSWGKRMSIQNWRKRPWLWAKRRRGWSAMSSWGKRSSLDTKENDDRQLLSV

>WWamide+allostatinB_Lottia_WWamide or allostatin B

MDLKTILCLIIYSLLLQISHAEEQLANDIELSNSLNPVDKRAWKSSYLNTWGKRWNPRYNLRGYQRMPIWAKRWTNSGLITWGKRSADTEIPIHKRKWNQFITWGKRSGVPSIVKRSVGDELVPWGKNKDTLPELNTSSDNLDNKLIDLETTPSTDKLSLDEKRASDKGWNGFTTWGKRANKDWSSLSTWGKRGQNKDWSSLTTWGKRGHDRDWNSLTTWGKRANKDWSSLSTWGKRARENDWSALSTWGKRANNKDWASLTTWGKRANDRDWNSLTTWGKRAKGNNWSGLTTWGKRANKDWNSLTTWGKRANKDWNSLTTWGKRGNKDWSGLTTWGKRGNKDWSGLTTWGKRGNKDWSGLTTWGKRGNKDWSGLTTWGKRGNKDWSGLTTWGKRGNKDWSGLTTWGKRGNKDWSGLTTWGKRGNKDWSGLTTWGKRSPDATSEDSGELSTLDKKDIKGWNGLTTWGKRFAGDKNKWSSLTTWGKRDDNNKQDDKKWAQLSTWGKRSPEDAAELWKIYDSNGDGIMDKEEMVSFLRSAASQKDSQQDEKS

>WWamide_C.gigas_gi|405955676|gb|EKC22697.1| hypothetical protein CGI_10001623 WWamide [Crassostrea gigas]

MIWVFCTPKEDQKGHLEPGIMLCHLEQLLLSCIVLCLVKVCASLKAQDEASVNDHDIVRRQAMGHSFGDELEGFDLDPKRVNNWNQFPAWGKRLSKRRWSSLGAWGKRSWLDRLISANNNWGKRWKSMSNSWGKRQAPSEFDGLSDDYINIKKRSVDSKFSHNSRNKRSIPTELSPEQNEEKRRWSSLSAWGKRSDDDEKRRWSSLSAWGKRSNPEAIDDNDSDNISKRKWSSFSSWGKRGDPVDLSKRLYSYWQNRLMTNNPWMERRGWNAFSSWGKRSMD

>NPY_A.californica_gi|155794|gb|AAA27772.1| neuropeptide Y [Aplysia californica]

MQRVILVVLLLSCMAVLSVRADNSEMLAPPPRPEEFTSAQQLRQYLAALNEYYSIMGRPRFGKRGDSFRKREFFRTNGERYPEDAAAWTEFQ

>NPY_L.stagnalis_gi|6580601|emb|CAB63265.1| neuropeptide Y [Lymnaea stagnalis]

MHKLLLVSLLVLSLAVMEVLCTEAMLTPPQRPEEFKNPNELRKYLKALNEYYAIVGRPRFGKRNGARVSDMFRPSGDDFGDYSANWGDF

>NPY_H.sapiens_gi|189274|gb|AAA59944.1| neuropeptide Y [Homo sapiens]

MLGNKRLGLSGLTLALSLLVCLGALAEAYPSKPDNPGEDAPAEDMARYYSALRHYINLITRQRYGKRSSPETLISDLLMRESTENVPRTRLEDPAMW

>NPY_R.norvegicus_gi|205761|gb|AAA41723.1| neuropeptide Y [Rattus norvegicus]

MMLGNKRMGLCGLTLALSLLVCLGILAEGYPSKPDNPGEDAPAEDMARYYSALRHYINLITRQRYGKRSSPETLISDLLMRESTENAPRTRLEDPSMW

>NPY_A.japonica_Agi|396083818|gb|AFN84517.1| neuropeptide Y [Anguilla japonica]

MRLNLGTRMGMVMLAVCTLICIGTLAEGYPSKPDNPGEDAPAEELAKYYSALRHYINLITRQRYGKRSSDDTLLTELLLSESTGTSPRSRYVEPSMW

>NPY_C.gigas_gi|405957592|gb|EKC23793.1| Neuropeptide Y [Crassostrea gigas]

MVSVYANLFKTILARVLPSQICLLFIVTASWFCLNTASQLENMEMLAPPERPSSITNIDELRRYIKELNMYYLILARPRCVFGLTHSLIHHTI

>NPY_M.musculus_gi|148666197|gb|EDK98613.1| neuropeptide Y [Mus musculus]

MLGNKRMGLCGLTLALSLLVCLGILAEGYPSKPDNPGEDAPAEDMARYYSALRHYINLITRQRYGKRSSPETLISDLLMKESTENAPRTRLEDPSMW

>SNPF_gi|730169|sp|P41321.1|NPF_HELAS RecName: Full=Neuropeptide F; Short=NPF

STQMLSPPERPREFRHPNELRQYLKELNEYYAIMGRTRF

>gi|163562021|gb|FC808552.1|FC808552 CBGC9737.fwd CBGC Lottia gigantea 15h 18h embryos Lottia gigantea cDNA clone CBGC9737 5', mRNA sequence

MQKLVLSLLILAAVVMLEVTSQDSMLAPPDRPSEFRSPDELRRYLKALNEYYAIVGRPRFGRSVNKRSAADYLFKPSHKNE

>gi|117949199|gb|DB919572.1|DB919572 DB919572 Northern pygmy squid cDNA library Idiosepius paradoxus cDNA clone Ip_aB_035_I23 5', mRNA sequence

MQKTLLTTVVIAVVLIVQISSQEIMLSPPSRPAEVRNPNELREYMKALNEYYAIVGRPRFGRSLFSKKLGPSYLNEGLKAGENSE

>ELH1_P._fucata1

KRTYISLNGDMRSLAKMLMRHYGNRSVKRPVENYTSLRKKLYALG

>ELH2_P._fucata2

KRQRLSVNSALASLADMVSADGHRRMKEEMSSNHQRLLGLG

>ELH_C._gigas

KRGRLSLTADLRSLARMLEAHRKRFIASRFPYDSIRKKLFRYG

>ELH2_C._gigas2

KRQRLSVNGALSSLADMLAANGRQRMMSEMAMNRQRLFGLG

>ELH_L._gigantea

KRSRLSINQELKSLANLLVLRENKRREAQKTKLRSKLLSIG

>ELH2_Lottia._gigantea2

KRAGRLSINGALSSLADLLVSENQRRDRLESMELRQRLQYLG

>ELH_A._californica ELH

KRISINQDLKAITDMLLTEQIRERQRYLADLRPRLLEKG

>ELH_Lymnaea ELH precursor

KRLSITNDLRAIADSYLYDQHKLRERQEENLRRRFLELG

>ELH_Theba ELH

EAERDRRTWSISNALTVLTDMVVEHEQRRLAAEREALKQRLLEL

>ELH_Triton ELH

MTTMAFLLPILAAFFFLGLSSVQGLPTSKASHVTSGVAPERGLHKVTARSSGVKALTKRDISLNQDLKSLANMLLAREYDRILSNRMNREFLRKIGKRGSSSLVGGFEDVMDLLPGEPQEDSLPSWWKCSDCDEENKFGRNSAPPSPPPSPPSPPPSSPPSPPPSSPSPPPPSSPP

>Tpi-achatin

MASVSHFLLLAFTLAAAFNLIALHELDYLEDAELDSDEEDFLGKRGFADKRGFADKRGFADKRGFADKRGFADKRGFADKRGFADKRERISPLVLRLLQRSYQNQALPLTGHNAFSRLLAREGLLE

>Tpi-allatostatinC

MATPGVSKINLPTVVCLVAVALTVVVEMVASDGSSYGQTFQGSEISPVTYHDSPDNGFSGPTLNREDIRREMIRQLLILQ

>Tpi-allatotrophin

MSRPSVSAILGVAVICVCLCDLVVSDASSLRRQKRGFRGDAATRVAHGFGKRGFLHPASESSLQFSLGALDNSPASLEDVSDGVLMSVDDFAELLVNHRNLAKALLKKFVDIDGDGLVSTDELFRPVLKK

>Tpi-CCAP-1

MQELSVATSTSNRFVLTAVCILVCGTRFSTSEPSGDIIQLPGSSERNNQLSFSRPDSFRSISDDEGLRGLSDLLLVKQSMLNLLSKPSPAAADTELETEDQTDASTLTKRVFCNSFSGCGGRFRGRRRQQQPVEKIGKRRLPNLRKRPFCNSYGCFNSGKKRFSPASSKNLGSNMTPLSDALLQDRIKKLFCNGYGGCQNLGKRLGALDAAVAKAAGDEARLSLPLTTRSLLNNLGGMKRVFTSQFDDDIDSLIDSMRR

>Tpi-CCAP-2

MAPENFETTMTVSRSIVLCLSLIIYSSPVFADQPNQLYRQLTLSNTKNTQSGSYPRLSDVLLWKDVLADTLLHPTADGTDHNKIATFPAPQQTKRVFCNGFTGCGGRFRGRRRIPLPEDGFKGRHELGKRPFCNTYGCYNSGRKRSIEAPKEDIHEPSPAEDHRLVTVADQWPWNRIKKLFCNGYGGCQNMGKRLHIQPDSRLTDSGRSVSLPFSKESLDSDGVKKRFFTSGFDDDMDSLIDNMRR

>Tpi-cerebrin

MFPFRTLLTLTTLVAACWLLQTATASPIYTSSIDQKTRQDIFILAIRIAKLAQFGGGSMDDYQKRNGGTLDALYNMPDLEDAGR

>Tpi-elevinin

MTCQAVYRTILTTLACLLLSGNYFNVHVAAARRPGRVDCTRYVFAPVCRGVAAKRNVLPTRSSDEDMSPPNYLDDFVLMSFLSSVREQNLGRTRNNVDNLRQLLKQTLPIEDQRQERIFQEQRNEAEERSLLPKLLVRRQRSPGRTIH

>Tpi-Ffamide-1

MKSSLLFVVISMVAALLVTKSHVTAQRNIKDYVGQQPLLFGRRGLNPNRNHLFFGKRGAINSEVSLKELKSACAMILPYLEDVDSTVDEEV

>Tpi-Ffamide-2

MRSCCGCDTSEGPGTFRILYCRMKITIMLVIAVTMVTIVGAKYHYRETSREVRNQMMGQQPLFFGRRGPNPTTNSLLFGKRRGSGVSLSVLGRPHTWFFPTPYSGQAYRP

>Tpi-FMRFamide-1

MYHKVIEAVVSLTLVALCLGTEDNGEAHILDDDSEFYRFRRSLLQHFDADDEPYTGQNWLSHPRESLSPDLEDSALLRDRREFYRFGRNQLPLEEKRFLRFGRSVHATDSNMGDHTESIRPVYRKRRELGDDSQSEELLHLSKRSVQSNVARGDQKNSEANKNIQKRHTRPGNAKLDNQISENLEPEKRFMRFGKRFMRFGRGDEETGDYEKRFMRFGRDHEDDSDYEKRFMRFGKRFDDDEDVNKRFMRFGKRFMRFGRGNEDEEHNTKRFMRFGKNVDYNEDPEKRFMRFGKSGDYIQDPEKRFMRFGKSIDYIEDPEKRFMRFGKRFMRFGKRFMRFGRQGLENDNQLPNAYSNDIDVGHLEKRSR

>Tpi-FMRFamide-2

MYHKVIEAVVSLTLVALCLGTEDNGEAHILDDDSEFYRFRRSLLQHFDADDEPYTGQNWLSHPRESLSPDLEDSALLRDRREFYRFGRNQLPLEEKRFLRFGRSVHATDSNMGDHTESIRPVYRKRRELGDDSQSEELLHLSKRSVQSNVARGDQKNSEANKNIQKRHTRPGNAKLDNQISENLEPEKRFMRFGKRFMRFGRGDEETGDYEKRFMRFGKRFDDDEDVNKRFMRFGKRFMRFGRGNEDEEHNTKRFMRFGKNVDYNEDPEKRFMRFGKSGDYIQDPEKRFMRFGKSIDYIEDPEKRFMRFGKRFMRFGKRFMRFGRQGLENDNQLPNAYSNDIDVGHLEKRSR

>Tpi-FMRFamide-3

MNSLCLTLAPALLSLICLSSYGWAEDSNGIQTLDEEGSDPFFRHNRQFYRFGRAFVPLWDNADDSLIRKNLLTHWSAIPMSPALDDDVFSRNSRQFYRFGRAYPPYQDKRFLRFGRSQQPDIDEYLQSLHSDQALYRKRRSENVDLKEDGLNRIARSSGANLQSKNTENTKFGKDLQKRETKKEKINVNDDLDIVPNDDKDLEKRFMRFGKRFMRFGRGDEDESYDKRFMRFGKSFDDDQEFEKRFMRFGKRFMRFGRGDENDVREEKRFLRFGKNGNEDEDINKRFMRFGKSGNEEGDVDKRFMRFGKRFMRFGKSAKEDGDVDKRFMRFGKRFMRFGKSAKEDGDVDKRF

>Tpi-GGNG-1

MELTIGASVTFVSCLYLLVATSPVSGKCHGKWAIHACFGGNGKRADPNMNSDSSAEPSLLHRALISDAERLSRMLQLPLIAADPLAENENTAAAERNLGLSTEEYDYGDITDGSDDRYNSLLPRNVPDVQDLRRYIQALKLQHAMRKKEADIY

>Tpi-GGNG-2

MELSLGGSLALVLCLYLSVITEPAAAKCRGKWAIHACFGGNGKRSDPSVESGMLHKVLFPSARNLNRIFQSENAARPEEEIPVYEEDGMKDAATDDSYPRQDVIEAPEVRDFRRYLLALKLQQALRSTDQSLV

>Tpi-nsulin1a

MVRNVEYYTTLTVALIAINLAIHQVQGQRRTCSLVARPHPNGYCGERLAQAHSNICFLLRRTYPHLFPMSKRSVPNESHISQSLDSLPDWELDGDESTRYPDSKSDFPPTLEDNSYLLSLLDTSDELADTSDEQADTSDEQADTSDEQADTSDEQADTSERPTASAFINFLEKRNSRRKRSLVCECCYAPCSFRIIARYC

>Tpi-insulin1b

MVRNVEYYTTLTVALIAINLAIHQVQGQRRTCSLVARPHPNGYCGERLAQAHSNICFLLRRTYPHLFPMSKRSVPNESHISQSLDSLPDWELDGDESTRYPDSKSDFPPTLEDNSYLLSLLDTSDELADTSDEQADTSDEQADTSDEQADTSERPTASAFINFLEKRNSRRKRSLVCECCYAPCSFRIIARYC

>Tpi-insulin1c

MVRNVEYYTTLTVALIAINLAIHQVQGQRRTCSLVARPHPNGYCGERLAQAHSNICFLLRRTYPHLFPMSKRSVPNESHISQSLDSLPDWELDGDESTRYPDSKSDFPPTLEDNSYLLSLLDTSDELADTSDEQADTSDEQADTSERPTASAFINFLEKRNSRRKRSLVCECCYAPCSFRIIARYC

>Tpi-insulin1d

MVRNVEYYTTLTVALIAINLAIHQVQGQRRTCSLVARPHPNGYCGERLAQAHSNICFLLRRTYPHLFPMSKRSVPNESHISQSLDSLPDWELDGDESTRYPDSKSDFPPTLEDNSYLLSLLDTSDELADTSDEQADTSERPTASAFINFLEKRNSRRKRSLVCECCYAPCSFRIIARYC

>Tpi-insulin2a

MSNLISYMLSRACLFTLILTIVPHTGVTPVSAQKYCDHNSVPHPRGYCGVNLSNMVELVCESFKRPTSSRYIVKRQTDKILNDNLGSIALNKKDALSYLAKRATTGTITCECCYHACTFFELAQYCPLPGR

>Tpi-insulin2b

MSNLISYILHRACLLTLILTIVPHAGVTPVSAQKYCTSESLPHPRGYCGVNLSNMVELVCESFKRSTSSRYIVKRQTDKILNDNLGSIALNKKDALSYLAKRATTGTITCECCYHACTFFELAQYCPLPGR

>Tpi-insulin3

MSGNADHCAALTCILLTFTLAVNQGQGQKRSCDLLSRPHPNGICGSMLAQVHENVCFLMRQAYPHFFPLRKRSPVLGDEDNHGGFLLPPLKNVLVSGDGYRVGPAVVGDTPFSLRRNSNAISPRKTFGLPGDIPQDQSENPIQRVLNKRNARSRSLVCECCYGPCTRRILASYC

>Tpi-insulin4

MASLVKVCLGVAAICVLVDVIVSQGMTDTENRFIADTTNRFSRLSATELLNAWHTECHRRCNYQLTWHVEIACRFDPYRIQGRRRRSIEKPRNLTNILRTTSDVKVTQNDVNASQPAYPKDPPQFMPRNSAMSFLKDHSYKRKKRDVSISRECCRNKACSWEEFAEYCQGHSRRASDRDSVCTYD

>Tpi-LFRFamide-1

MVNGSCCTGVALLLAVICSVQSEETIHETGLHSEDDVHSHTAHQAQKRNAFTTPQEEDDGLILTNDGLADPLASNEFLDKRGSLFRFGKRQGSLFRFGKRGSLFRFGKRQGGTLFRFGKRGGSLFRFGRSDSAQNPMDNEEEKRTLFRFGKRSDL

>Tpi-LFRFamide-2

MNTMDYTLLTVSLGLLLSVSTCVNSEDLFSDSGSQPADSLNSPPLHTVEKRSPLIHYPDNDDNQDSVIEEPVDEFDSTYYADDADKRGGLFRFGKRQGSLFRFGKRQGSLFRFGKRGSLFRFGKRQGSLFRFGKRGGALFRFGRSGNAQDSENDAELDKRTLFRFGKRSDMLPHLLAQVDLLKNQPYQEHLDN

>Tpi-LRNFVamide-1

MTSFSSSLFGLLIAGQALSICVSQAGSNRTESPWTSPSVGSIEQHELYQQGPGTHETQPASAHYSSYRSKRDSGEADDDIYTYKRLRDFVGKRSHSELYQDFLEAPEDVPPSDNILNALESNNVADQLLIPRADKRLRDFVGKRSMSMNAHLWTPDVKRLRDFVGKRAFHAVKRLRDFVGKRALGFLGKRSHGVYEGDNKNEKRLRDFVGKRTQFEDIISPNEIWDEGNNRFVSHINKRPREFVGKRSGIFTYDNRNKRLRDFVGKRTSHFVMVPSAHFGKRLRDFVGKRVVSDIVNEDKPLLQFVGNSNNDEMHADNKRIREFIGKREVKRPREFVGKRSVSLNGENRIELQKKLRDFVGKRSSDGEFEQSKRLRDFVGKRLFNGEFEEDKRLRDFVGKRFSDGEFAEDKRLRDFVGKRTADDEELQEDMRLRDILGKGDIDEEFEENKRLRDFVGKRTPDGVFAEDKRLRDFVGKRAGGGEFEEDKRLRDFVGKRSIDGKVVEVKRLRDFVGKRTPDGEFEEVKRLRDFVGKRSVDGEFKADKRLRDFVGKRTPDGEFEEVKRLRDFVGKRSVDGEFKADK

>Tpi-LRNFVamide-2

MTSFSSSLFGLLIAGQALSICVSQAGSNRTESPWTSPSVGSIEQHELYQQGPGTHETQPASAHYSSYRSKRDSGEADDDIYTYKRLRDFVGKRSHSELYQDFLEAPEDVPPSDNILNALESNNVADQLLIPRADKRLRDFVGKRSMSMNAHLWTPDVKRLRDFVGKRAFHAVKRLRDFVGKRALGFLGKRSHGVYEGDNKNEKRLRDFVGKRTQFEDIISPNEIWDEGNNRFVSHINKRPREFVGKRSGIFTYDNRNKRLRDFVGKRTSHFVMVPSAHFGKRLRDFVGKRVVSDIVNEDKPLLQFVGNSNNDEMHADNKRIREFIGKREVKRPREFVGKRSVSLNGENRIELQKKLRDFVGKRSSDGEFEQSKRLRDFVGKRLFNGEFEEDKRLRDFVGKRFSDGEFAEDKRLRDFVGKRTADDEELQEDMRLRDILGKGDIDEEFEENKRLRDFVGKRTPDGVFAEDKRLRDFVGKRAGGGEFEEDKRLRDFVGKRSEFGNESSETLSVMKRLRDFVGKRAFSGDTTLPVYVKRIREFIGRR

>Tpi-LRNFVamide-3

MSPVYRSIFCLFVIIQVISVGLCATNQASSDRSIHDQHLHSASKTDAEQHSIDKRETVGVTADKRLRDFVGKRDPSEPSERQPVTSLHSFPELPLEAAVDYEERQLGNGDDETGTQEEGLEQLSQFPPQQISFGGPLILPPNYKRLRDFVGKRDEKRLRDFVGKRMREFVGKRSNVPYGFINEKRLRDFVGKRSDFIEDPIADESDVSKRLRDFVGKRAREFVGKRDDISKRLRDFVGKRDVYEPSDLLPSYDKRLRDFVGKREAFEIFDEDKRLRDFVGKRDGDDVFDEDKRLRDFVGKRDDKRNREFVGKRAREFVGKRAREFV

>Tpi-luqin

MKTTHNLMASCCILVVIFSVFSLGQTWRPQGRFGKRLSPDGAVVPDFGLSLSNEIPIEAFFSIKDVSRFKSKPRLCSLSGLQGYPLCDLSLLPTTAETDDWSNVFDA

>Tpi_Myomodulin-1-like1

MQISCVISLAFVVSLQFTVGFSDDQADSKASTDTALSRAKRESYNMLRLGRGLNMLRLGKRNDESTAQDEDDLEDMLAWRAGYYEPYTLDSYEHAYPEDDIEVPAHGRFRRSTPSSKAGLSSEVAQQLESSPKDSIPADLKLNAEEDQFENFPDDNVNFYDDFADGIIQPTEEGDEQDKRSLGMLRLGKRQLSMLRLGKRSLGMLRLGKREPEDDEYKRSLGMLRLGKRQLSMLRLGKRALGMLRLGKREDENFDDIDSEDGKRSMSMLRLGKRPMSMLRLGKRPMSMLRLGKRPMSMLRLGKRPMSMLRLGKRPMSMLRLGKREDDEKRSLGMLRLGKRSTQ

>Tpi_Myomodulin-1-like2

MQISCVISLAFVVSLQFTVGFSDDQADSKASTDTALSRAKRESYNMLRLGRGLNMLRLGKRNDESTAQDEDDLEDMLAWRAGYYEPYTLDSYEHAYPEDDIEVPAHGRFRRSTPSSKAGLSSEVAQQLESSPKDSIPADLKLNAEEDQFENFPDDNVNFYDDFADGIIQPTEEGDEQDKRSLGMLRLGKRQLSMLRLGKRSLGMLRLGKREPEDDEYKRSLGMLRLGKRQLSMLRLGKRALGMLRLGKREDENFDDIDSEDGKRSMSMLRLGKRPMSMLRLGKRPMSMLRLGKRPMSMLRLGKREDDEKRSLGMLRLGKRSTQ

>Tpi-NdWFamide_1

MSKVAAMVIVLVVVFNCIFSPADANWFGKRGSKDSLYSLLLQQPHLGTSRLDNSLLETPRLETSQLQRSTEAQDEDIDPVQFAIDDLETAAADRWADEDQ

>Tpi-NKY-1

MVWTINTLLKLALLAFVPLCYSRPPLLDGEDDMGYFLDQESPRRLYVPVEVPLDALLAALRAHTQSSHQPNALLDKRASSASEILGSALPKLDDDTDVRGMKRKMFWQPLGYLPASVRAHNSPTGSTGSENQGSSSNVFRYG

>Tpi-NKY-2

MQPTSCAIVMVTLLSVIAVCFSRPSSLLGEDSVNYRQDQDLYLPASMSCSAILRVLKAHNLHSLDYKTAEKKSAANTKSLDELLPNTDADIRMAGIKRRIFWQPLGYMPAAARANNGPVAVTGSGNQGISSNVFRYG

>Tpi-NPY

MLKFVLILLVVLSVAIMEATCNTQMLSPPERPREFKHPNELRQYLKELNEYYAIMGRPRFGKRNGAVGNFDYLSPNDWEDILSSQ

>Tpi-PKYMDT

MEAKTHQIFAVYILLTTLSVIDGIPTRNILQQETRISLSKRPKYMDTRRDLDILKDLILLSFQELADEESLQSASVSDDDSFATVEKRERYMGICMRKKYNNFIPFPCLRGG

>Tpi-PXFVamide

MQSITVLFLMTSCLSLIVAADKQSAYRVSSNIDASPAQLPQQLQPSQQQQRQQQQQLSSRLQASDQQQASSRTLSNASHNQLLEANTPSHGWDGSNALSDSSVSDGSVVSDGSHFNQVQSSNEDYDRYNLRNVIEDLLHIPMKSNWLLPQSDNSKHLFKNNYYSEYMPRWLQNHNNDTHQQHDDHHLDKREVTDQLRSGEEQPDPEILDDYYPILLDRRFQVYRSNDPFSSSPYLSAQEFKETFRRSDPYVVGKRTTDLLDDLMIPVSEDKRVAPKFVGRRDPPYFVGKREVFNIPDSYLAHLDLEEYQTPLLENRRAAPKFVGRRGAPYFVGKRRAPMFVGKRVPIMTLENDDSWTLDNKRAAPRFVGKRRAPFFVGKRENPYNLQVSWSDLSSGNNLDWLLEKKGAPKFVGRRGAPYFVGKRGPPMFIGKRSTDSETDSQSAFGYGQSEEASLLGRRGSPYFVGKRGAPRFVGKRWAEHGLQGLASSSTHTKRRAPKFVGRRGSPYFVGKRGLKQFGEDWLKEDGAEEESQTLSQFKRAAPAFVGKRRAPYFVGKRGPPRFVGRRLADSEWSDPSLLALEDKQRPVKFIDRRAAPFFVGKKAAPRFVGKRFLSSLYDNKGEQRLKELLVENNRNQVSDVVGKHSSEDPSSNVNSVSHEQTLSNADDQLMFGDLKAHIQANRIHELNRLRQNED

>Tpi-Pleurin

MQHQIRALVLFLLSGSACAMFYPRPRDYPRLGKRSFFTTVNGNHYPRIGRRDATGSTLVLPEADYADLGDLTKRGVFTQGAHGSYPRVGRGGAVLSRDYLNEGSKDLEKMSGDKDDDLDVDSGDGNLRLQTGHSRIPLEFLFIAYDSDNDGKLSKEEFVTGLNQYQLQCPIY

>Uniprot_AVP_Hsap

MPDTMLPACFLGLLAFSSACYFQNCPRGGKRAMSDLELRQCLPCGPGGKGRCFGPSICCADELGCFVGTAEALRCQEENYLPSPCQSGQKACGSGGRCAAFGVCCNDESCVTEPECREGFHRRARASDRSNATQLDGPAGALLLRLVQLAGAPEPFEPAQPDAY

>Uniprot_Oxt_Hsap

MAGPSLACCLLGLLALTSACYIQNCPLGGKRAAPDLDVRKCLPCGPGGKGRCFGPNICCAEELGCFVGTAEALRCQEENYLPSPCQSGQKACGSGGRCAVLGLCCSPDGCHADPACDAEATFSQR

>Uniprot_Vasotocin_Trub

MPQCALLLSLLGLLALSSACYIQNCPRGGKRALPETGIRQCMSCGPRDRGRCFGPNICCGEGLGCLMGSPETARCAGENYLLTPCQAGGRPCGSEGGRCAVSGLCCNSESCAVDSDCLGETEALEPGDSSAGSSPTELLLRLLHMSSRVEPHKCTLI

>Uniprot_Isotocin_Trub

MTGTAISVCLLFLLSVCSACYISNCPIGGKRSIMDAPQRKCMSCGPGDRGRCFGPGICCGESFGCLMGSPESARCAEENYLLTPCQAGGRPCGSEGGRCASSGLCCDAESCTMDQSCLSEEEGDERGSLFDGSDSGDVILKLLRLAGLTSPHQTH

>Ensembl_ENSPMAT00000000263_Pmar

MARCAPLTLAVSVLSLVLISSACYIQNCPRGGKRDLTDSVRQRQCLPCGPGGQGRCFGPRICCGEAMGCRLGGPDVAICRAERLMPSPCESRGEPCGHGGKCGAPGLCCSSGEVLQRP

>Ghost_KH.C6.11.v1.A.SL1-1_Cint

MYRQFSIVLILLANSLILDACFFRDCSNMDWYRKRGQEITDPRKQLFQELPEFLDNRPFQCNKDESNTGNECKFSGQKICCQETDDGQVICAVSATELQHRFLSSGVLQQVQRILHEFVEISSRGAPQKRRYCIKVGVCCSWDRCRPQVSCSYKDQDIPSHKEVFQDQTALVNRISHLLQDMY

>Bflo_jgi_84802

MWRFGMVLSVVLILVVVAATVGHSAGCYIINCPRGGKRALENRSGPAARECPRCGPSLSGQCIGPTTCCSPQAGCTRSLRVALECSMENLVPVPCRLSGPSCTLPGQQTGTCVGEGMCCMDGEKCSLSPECSMRTAEDEEARRQERRSVLPNWVLPGNTPPDIQKWW

>Skow_gi_187155721

MFGTPERLSWKVLTIAVIFVCATTTCEACFISDCARGGKRSPGSALTGAGHPLRQCISCGSNNSGHCIGPSICCGDFGCYFGTQETAVCQEEKSIPVPCESKGTPCGEYGHGNCVADKICCEEYNCSYDANCTTKNDGFQKADSSSSTAFKRAFKDLLEDK

>GLEAN_06899_Spur

MMSVKSIVTCLFLSLVLALWIGGSFACFISNCPKGGKRSNSRPLRQCLECGPGGVGRCMGPGICCGPTIGCHINTQHTLSCMRENEISTPCELPGNPCQTVPSGTCGAMGVCCNSNSCSEDASCLMIEEDDSLKRFEQMSREENGSTRKDLRVKLLDLLLNMQDQ

>jgi_173251_Ctel

MRHSSDAVNSVVLCGRLLLLFACISCCIETTSGCFIRNCPIGGKRSSVPSRISAQKECMACGPNGLGQCVGPNTCCGQDIGCFMGTQEAKMCGEENDSPIPCRVDGAACGRNDGGRCVAEQICCNEDKCSHDSSCQSKAKRQHDNLSQDLLRYMHQLMTLKSLGGRR

>jgi_53893_Lgig

MCFSSFQYILSLFVFMCVIFGANSCFIRNCPTGGKRAIEASEIGHKCMSCGPGNVGQCVGPNICCGRFGCYIGTKETEICEHENDSTVACRVEGKLCGSRQQGQCVANGICCDS

>jgi_59567_Dpul

MAGLWTFCLIALSMTEMIIPLTAKPCFITNCPPGGKRSSQLVEPSSYLECAPCGPAGKGTCLGANLCCGSHFGCFFKTEETNVCLLTNLKSTQICNQHFWKTDLKSASCSLNGDKIDGICVADLLCCSLGNLPQDDL

>gi_145651810_Tcas

MSTIITSIILLVLSESLVSGCLITNCPRGGKRSKFAISENAVKPCVSCGPGQSGQCFGPSICCGPFGCLVGTPETLRCQREGFFHEREPCIAGSAPCRKNTGRCAFDGICCSQDSCHADKSCASDDKSPIDLYTLINYQAELAGDK

>Ensembl_F39C12.4_Cele

MGSSPILLVLAISIGLASACFLNSCPYRRYGRTIRCSSCGIENEGVCISEGRCCTNEECFMSTECSYSAVCPELFCKIGHHPGYCMKKGYCCTQGGCQTSAMC

>TK1_Hsap

MKILVALAVFFLVSTQLFAEEIGANDDLNYWSDWYDSDQIKEELPEPFEHLLQRIARRPKPQQFFGLMGKRDADSSIEKQVALLKALYGHGQISHKRHKTDSFVGLMGKRALNSVAYERSAMQNYERRR

>TK3_Hsap

MRIMLLFTAILAFSLAQSFGAVCKEPQEEVVPGGGRSKRDPDLYQLLQRLFKSHSSLEGLLKALSQASTDPKESTSPEKRDMHDFFVGLMGKRSVQPDSPTDVNQENVPSFGILKYPPRAE

>TK4_Hsap

MLPCLALLLLMELSVCTVAGDGGEEQTLSTEAETWEGAGPSIQLQLQEVKTGKASQFFGLMGKRVGGRPLIQPRRKKGREDEAQGSE

>Ensembl_ENSTRUP00000014475_Trub

MKFLLLSALVALCAVTRVWCQEIDPKEEADYWSSNRIQDGWFPNSPLREILLRMTRKPRPHQFIGLMGKRSMANAQITHKRHKINSFVGLMGKRSQEEPDSYEWSTIQTYDKRR

>gi_385655153_Drer

MKFILPTVVIFVVLCQVFGEELGPKEDLDYWTGNNQIQDEWIQSDPFREILRRMTRKPRPHQFIGLMGKRSSANAQITRKIYLHKINSFVGLMGKRSQEEPESYEWGTVQIYDKRR

>gi_48638786_Cint

MQGLCYRNAMSVLSLILVATISISTAKFPDTASNDFPLENFVLDQLCGQMKADQTLSNRLRNVDYQRDDDLRYLDQLQEKRQRDLYEKNKRHVRHFYGLMGKRSIGDQPSIFNERASFTGLMGKRGPIPYGRDSNILNPEPRLPLQDKTYNGDYLFGVPQNDRDAIGPDNVQNDNPVANRMMQAILSTILNKYCDEN

>gi_27530027_Ovul

MIRVGLILCCIFIAGVFEASSADDMLTAHNLIKRSEVKPPSSSEFIGLMGRSEELTRRLIQHPGSMSETSKRGPPKKVSRRPYILKK

>jgi_173630_Lgig

MWIYLATTSLFLLCKVCAINGAAIYDAGSINQNFQEGLNEPLETPLPRVPSPHIIDDAFSSLNKWRNSDDLSKQKILSSFLKEIPVLTNGYAINQYPLSKRHPNFGFMGSRGKRTQDDIGASDFLNTIRPGFRRALETLRSQQMQGLSPELSNSKRQPAFGFHAVRG

>gi_163525452_Lgig

MNQDCGFYILLVSVFVLFSLQIVLADDTELYKNNEEKRFAAFNDFSNPETPYSKQYWFYPHTSQGFLKTLLRYLALNEQSYRNTVKRQRTFGFVGTRGKRGDTFDGLKDFVRKLQDEINRRKYGSMLLDNSMDNSQNEGYLKLKRQPHLGFHGMRG

>gi_121357157_Acal

MKQAHLSLALVIGLVSLTLCNSAPMDSSAEAASYMKWPEIESEGEQYWDLPKEPSALKPQSTGSDLEGDVSLGSDFTQPKNRLPFVLDENLLLRLRNELAALRDHQLAASDQQGSGMEKKFKPSGFMGSRGKRFMPGLESLLLAKYYQDAKWKRQPHLGFHGSRG

>gi_161289578_Ctel (partial)

MGTQSRQPGAAIQMAQYYHDVMLFTKVPTSTCQNIHFRLSMPPCITYGVCAVLFVICVHANELIGSSEDDINELLSLNPESSVLTYLPDGITKDDLIDLSKRLSRGFYAARGKKFSPKSFHFSRGKKLASEADEDSLYGFSADDMKRAYPSGFTMPRGRRVPLGFQMVRGKKSSGEKRGLNKSSFFLARGKKSHEDDDHLLNTSSDPKDDLQLLMKYFNNIRTGSAVH

>gi_223742217_Apom

SDVDGSDVISGDKRFSPNSFGFSRGRRLNPASFSFNRGKKADHVIYDDIMAAERYEKRAPPSGFNGVRGKRRPPAGYVVVRGKRVPPSGFTGLRGKKAAPSGDVQGYDWREKKSPPEGFLGLRGKKAAAPDVVNADTGSDERRLRHYIASMLHAERAPSGESDNKPDFRFALN

>jgi_160489867_Dple

MGASKRCIILLTVLLAYVSAQEVVRRVPQGFLGMRGKKYFEVGGPDQLYKRKPQFFVGVKGKKGLYDDAEYKRVPMGFVGMRGKKTLMSDFMNYPESFEYVPKRSGSLIGQIDYSTDDNIPEYPVLNEVIEEYLQKLRDENTEVPAETSDVDDSGLFTNEVGKRANIHKFFGVRGKKSGQVKRPYDVSFRGKFIGVRGKKDLKNSGQEPF

>gi_282701557_Pmai

MDGQTWLGLVLMCVALSWAENTVDRRASNMGFVGMRGKKSSKDDDLQKDKRAMGFVGMRGKKDMDIEEDKRAVGFVGMRGKKDDYLDEKRTMGFVGMRGKKDPQDYLFEEDKRAKGFMGMRGKKDLIGEEVDKRAVEFMGMRGKKYYDFDYPDQESYEKRAMGFQGMRGKKDACLHQKVTSWICVYAGKRDEDFTLMI

>gi_254014433_Onub

MVTPRTCFLFITIQLVYVISAQEMEKRVPQGFVGMRGKKYDEPTEQFYKRKPQFFVGVKGKKSIFDLLEEPEDKRAPMGFVGMRGKKELYLPETYYGNYDYVPKRGAGSLIGQIDFASNEDVKSFSGGDEFPILNEILNEYLQKVEHSNSLESATDVPDINAEFSNERISNEVDKRAANIHQFFGVRGKKSIQNKRPYDL

>jgi_236286_Dpul

MAVLMTVLAYCQPASAAATAVTDDDELMARQTRGLVLRSWRNAQQQTDHSADKTPSAKVAEPILPSQKEAMVFNGLPISMRLVLLQHLAGYDKRTPNSRAFLGMRGKKSSPPGADALTMEDNQLDDASGWPQGDILPDTYYFGPAPQKKKMHGEKFLGMRGKKMMNGLADGTAFIPNWRERYIYQEPFEKKRAPSSNSFMGMRGKRSESTTPTPNDYQFFNDDIIVDEELPDVDSKVSPRRSQERTPI

>GLEAN_05685_Tcas

MHSTTITTAVVLATIYVVCAAEDHHKRAPSGFTGVRGKKSIPDSAYSTGNSDSDSIPELKAVDIVSDLGAVDKRAPSGFMGMRGKKPFSLWEGTYPDGVFKRAPSGFMGMRGKKDMEFANYDEYIKRAPSGFMGMRGKKDYDSSSSQLDKRAPMGFMGMRGKKDYDEIADEKRAPSGFFGMRGKKMPRQAGFFGMRGKKYPYQFRGKFVGVRGKKASPDDYYNVDLDTVGQELDLNQLMLLLTENEGESDIWNGNNEVGQYSQK

>FBpp0081962_Dmel

MRPLSGLIALALLLLLLLTAPSSAADTETESSGSPLTPGAEEPRRVVKRAPTSSFIGMRGKKDEEHDTSEGNWLGSGPDPLDYADEEADSSYAENGRRLKKAPLAFVGLRGKKFIPINNRLSDVLQSLEEERLRDSLLQDFFDRVAGRDGSAVGKRAPTGFTGMRGKRPALLAGDDDAEADEATELQQKRAPVNSFVGMRGKKDVSHQHYKRAALSDSYDLRGKQQRFADFNSKFVAVRGKKSDLEGNGVGIGDDHEQALVHPWLYLWGEKRAPNGFLGMRGKRPALFE

>Ensembl_T07C12.15_Cele

MISKCSVMGLLLLLFVHITTAQFETHFDDAQVYWPSQYKRSGPSSASEGEAYAFPGLRGLRGKRDPTYHKRVPMMSLKGLRGKRARIFDGQEEQ

>Contig211_blast_Ppac

MRSGGREGIDTNLYDVAPGFMTNKRSEFEAPRGFRGMRGKRAPFKGLRGKRGGENPFEMPLEY

>Uniprot_GnRH_Hsap

MKPIQKLLAGLILLTWCVEGCSSQHWSYGLRPGGKRDAENLIDSFQEIVKEVGQLAETQRFECTTHQPRSPLRDLKGALESLIEEETGQKKI

>Uniprot_GnRH2_Hsap

MASSRRGLLLLLLLTAHLGPSEAQHWSHGWYPGGKRALSSAQDPQNALRPPGRALDTAAGSPVQTAHGLPSDALAPLDDSMPWEGRTTAQWSLHRKRHLARTLLTAAREPRPAPPSSNKV

>Ensembl_ENSPMAT00000011357_Pmar

MGRASLSLVLILWLLTAPPASLGQHWSHGWFPGGKRGVQEPPRASYENVSPSDGSPFTPVSSGLQVADWHVVCSRSPNGFSGCAMCCSPGCPTFLSQVLEASLGT

>Ghost_KH.S1051.1.v1.C.SL1-1_Cint

MLDIEKDELAALLQRENSAFRDILYHKNAGNFEKSDSGKFDSLKLQNNFPHLDLGLGVDLDAVDQWNRYKQANAQRMQNLGVPVNARQHWSYEFMPGGRRAVWENANVGVPVSRQHWSYEYMPGGRRSAGQHAMTKRQHWSKGYSPGGKRSVDLSEFDDQGRRITKHEGMPEEPFKVEQPRPRNGIHGPAGLDQNEPDWKNWMNEQPAASSDDKGSDVE

>Ghost_KH.C9.484.v1.A.SL1-1_Cint

MTSLVICILSLFVFLHVAQCHVLRNQEALGSFDWDEENPEVRPDFEDELPAESFQNLPSNNEERRQHWSYALSPGGKRQHWSLALSPGGKRQHWSNQLTPGGKRVIPRMREQKKADFDEINYTKIYNLLRQYLEAAAEYEKGDFGRYKGNQRNQLETIKDDIMTE

>Ghost_KH.S1104.4.v1.A.SL1-1_Cint

MHKISVCVLLVVLASIQSIEAQHWSNWWIPGAPGYNGGKRPLWKGKMEIRQNYRTTPRDHDVAEDSADEAFEHGDNSRDETAITMDDLLKKLAMIRQHEKDEQLLNAKIKNGLPRWENSKNRKLEFTVGLLMLRSAIFGPKLAGEWKQHFAVE

>ENSCSAVP00000006019_Csav

MKSVISLLSLLVIINVVYCHVLPNRGGQANFDWTRQKSDISPDFGADYPSDSSEEIPPLGANRRQHWSYALSPGGKRQHWSLALSPGGKRQHWSNKLAPGGKRVNPYSSAKANPVTDEENYNKLYNLLRQYIQAAIEFDDDLTGYKDDDVTLDNIEV

>GLEAN_19680_Spur

MKQIITSLVSISAALLLFVLISEYTPRCNGQVHHRFSGWRPGGKKRSDAAEVNSNKITIERPQLPICQTTEERQLLEGDSDILGDLRRAANRMRLLQLFNLSKTRLNDLNDATSNEVDERPVYGDYLGTGL

>Crz_Dmel

MLRLLLLPLFLFTLSMCMGQTFQYSRGWTNGKRSFNAASPLLANGHLHRASELGLTDLYDLQDWSSDRRLERCLSQLQRSLIARNCVPGSDFNANRVDPDPENSAHPRLSNSNGENVLYSSANIPNRHRQSNELLEELSAAGGASAEPNVFGKH

>AKH_Dmel

MNPKSEVLIAAVLFMLLACVQCQLTFSPDWGKRSVGGAGPGTFFETQQGNCKTSNEMLLEIFRFVQSQAQLFLDCKHRE

>F36H12.1_nlp-47_Cele

MQLYVVLCFLVLLGLSAGQMTFTDQWTKKRATLHKQLPVVTPEEPICPSDRVQAVFEQLDQLQKAQQRLTEYLASCAYPVEVPQKAEKM

>Uniprot_CCK_Hsap

MNSGVCLCVLMAVLAAGALTQPVPPADPAGSGLQRAEEAPRRQLRVSQRTDGESRAHLGALLARYIQQARKAPSGRMSIVKNLQNLDPSHRISDRDYMGWMDFGRRSAEEYEYPS

>Uniprot_CCK_Drer

MNSGVCVCVILPALSVSVSCASRPVSDERFLSARRLARSASLTLQQPLPPAGDIQPDTRANFSQLLAKLISSKKGSVRRNSSMNSRANSVNHRIKDRDYVGWMDFGRRSAEEYEYSS

>gi_204273783_Ipun

MNCGVCVCVLLAALSVTFAARTRSSPAQQENDALPLQMDSSVSAGHDANPRANLNELLARLISRKGSVRRNSMANSKASALSANHRIKDRDYLGWMDFGRRSAEEYDYSS

>AL667803_Cint

MRSKIVIYFCILVIVTLNVNGVPTSDLFKSVSQYHLPRGKVINKETATKPLQFQRAICRLLQKLGEETFSRLSQSELEAKQLDLIKTCYQANSFGGNENDGHMQRMDRNYYGWMDFGKRGIKDVDYEY

>gi_187061456_Skow

MKKLIVFLGIAVLFLIVKGDEVTDELNSAEEVDGASSDESKRDHYGWMNFKKSDPEKKDHYGWMNFKKSDPEKKDHYGWMNFKKSDPEKKDHYGWMNFKKSDPEKKDHYGWMNFKKSDPEKKDHYGWMNFKKSDPEKKDHYGWMNFKKSDPEKKDHYGWMNFKKSDPEKKDYYGWMNFKKSDPEKKDHYGWMN

>gi_115694514_Spur

MGTRWFLALLSLFFMLLAPVTSFPTRSTEQSHKNLPGVTSFLRMLKDHIVNRSRGTGSRGPLRGPKGHHQVTPNVNSHNSEVLPAWPPKSKTEDWVLDLAPKDDLSNLANDSERDFSPDVAAAVGSTELEDDKRDYGHGMFFGKRVPNDKYIDRQLEDPTMQRQRRPDDYNWGMWFGKRDKADLYGWGGFFGKRSGDVTEYEDFVA

>jgi_Ctel_228037

MKVNYAALSLSLGVVTLLVAQMCAASVSTDDSENVRNEILILAKLLRPIRQLAGRLEDLESIIERAATDREFAMEVAKRQGAAWDMDYGWGGGRFGKRDSGKRTSDSSKRYDAFGLGGRFGRSVDHVDLEKNE

>gi_260626388_Cgig

MKNSLEHLLISSVLVLCPLFTLTKGNSHAQNSIIQLSKLFSNLQDIHDAKQTQEENTKDQVRSDTRKQRGEIELILALGAPVHVESLQHNDKDTQTDTDKSAWDVDFEDDQQDVEKRQGAWDYDYGLGGGRFGKRFDYNFGGGRWGRDVDHVKQGQAK

>CCK_Haliotis_diversicolor

MDHYMTTVTAVILAFVCVTSLPVNEGDDVSNLANLLGKLRPLHRKRLDSHSRGHFKSTSGNDDKPQSSDLADSKSGVAEDPEDLLTEDVSDMRKRQGAWSYDYGLGGGRFGKRNYGEYGFGGGRFGRDVDHVDLSD

>gi_163526260_Lgig

MDQNISLLIICGLFVLVTSLPLKTDDDNVEHLVSLIKKLKSLEKRPETNSRWLDHQLHHLRIDNSPAEKSDSLTSGERDSGILYRSLNSDMDTPSKRQGAWSYDYGLGGGRFGKRSYDDYGIGGGRFGRDVDHVDLADGVLNGA

>gi_121341705_Acal

MEPQLLSVVILVLCACAASALPTNSRREDLSHLVTLLGKLKNIERARQNSEGSAQAHAQEAWEGPRVSSFPETEVTADVIPDEVSAAKGYLGQRPATGVVNKRQGAWSYDYGLGGGRFGKRSYGDYGIGGGRFGRDVDHVDLSDATENEITS

>gi_223770502_Apom

MKGFAQLLFSFAVALLMIMHVTGSEGSSIGDNSVNEVQSKELLFLAKIVGPLKEIARESEQLASIVERVATDGDLTQELSKRQGAWDMDYGWGGGRFGKRASGTSISQSGKRYDSFGIAGRFGRSVENVEGH

>Ensembl_FBtr0078989_Dmel

MGPRSCTHFATLFMPLWALAFCFLVVLPIPAQTTSLQNAKDDRRLQELESKIGGEIDQPIANLVGPSFSLFGDRRNQKTMSFGRRVPLISRPIIPIELDLLMDNDDERTKAKRFDDYGHMRFGKRGGDDQFDDYGHMRFGR

>jgi_242979_Daphnia

MKLTMLATVLAAVLVLGVGRATAAPADSSSTATGRRLLHSPNPTSHSKSIDSWLRWLLLRSRIGDKEKTKNGVPSNSFQLARSPVELGSSNPKLQAKLPPAIVQSNDDDETTGFGDEDFADEDVPLVLPEGRQAASKRQPDDYGHMRYGDFDDYGHMRFGRR

>Uniprot_NMU_Hsap

MLRTESCRPRSPAGQVAAASPLLLLLLLLAWCAGACRGAPILPQGLQPEQQLQLWNEIDDTCSSFLSIDSQPQASNALEELCFMIMGMLPKPQEQDEKDNTKRFLFHYSKTQKLGKSNVVSSVVHPLLQLVPHLHERRMKRFRVDEEFQSPFASQSRGYFLFRPRNGRRSAGFI

>Ensembl_ENSDART00000063564_Drer

MSPGNTSALMLAVLLLSFIPITTSAPMLLNPSSLEHEQLLTQITDLCSFYLSADPSFRTSDVLEDLCFLMLGSLQKSKEITARETSKRENLQGPGRIQSRGYFLYRPRNGRRSDEYV

>jgi_149279_Lgig

MEPSPVKIVACFVIFAVIGTEAMNYMAFPRLGRSGYLAFPRLGRSDGQTIQSSEFEQAECCPEGIKSHWIIIGASKPEIRSRCSPKSVCCEGLEELVGRSSDHIFYTKCTKITDEKTENTVLDRLLRKS

>jgi_231562_Lgig

MTSLTCLLCNGAPTEDSLSRVRRGGWSMLRLGRGLQMLRLGKRTHPSGLDIYLDRDLHERQVPLPRYGKDLDWQNFLERHMLGNTEMDEEKRESMLPLTDPFLSEAIDGYPHMRPAPRGGRFKRSAGRFRYYPEVRSEERAVALPRFGRLIEKELKHKDGNDESKERAVPAPRFGRNPH

>jgi_312704_Dpul

MRIAIIHSLVLVVIYLAYSDAAPPQILKSQSLIPFPRVGRSRSSFIANAVGSARSGGAGNPMMMGSGGNANGKSPNNWMMNNADIKRHLIPFPRVGKRQNLIPFPRVGRAGYYQPGFFPSTDDEEGQASIQQQFALSSEESQASAPSSFLMSGSDILGALNNGRSNSDERTAVFIPRRWMTNSQESEE

>GLEAN_08429_Tcas

MKTFLIYSACVVLFCIANCQGEPKEPKRNKLASVYALTPSLRVGRRSEGTDVKRRIGKMVSFPRIGRSESNWVPDDNSYGAQRPGANSGGMWFGPRLGRVQKRSENFTPWAYIILNGEAPIIREVHYSPRLGRESEEAYEEILDSNLDVL

>Ensembl_FBtr0085514_capability_Dmel

MKSMLVHIVLVIFIIAEFSTAETDHDKNRRGANMGLYAFPRVGRSDPSLANSLRDGLEAGVLDGIYGDASQEDYNEADFQKKASGLVAFPRVGRGDAELRKWAHLLALQQVLDKRTGPSASSGLWFGPRLGKRSVDAKSFADISKGQKELN

>Ensembl_FBtr0082639_hugin_Dmel

MCGPSYCTLLLIAASCYILVCSHAKSLQGTSKLDLGNHISAGSARGSLSPASPALSEARQKRAMGDYKELTDIIDELEENSLAQKASATMQVAAMPPQGQEFDLDTMPPLTYYLLLQKLRQLQSNGEPAYRVRTPRLGRSIDSWRLLDAEGATGMAGGEEAIGGQFMQRMVKKSVPFKPRLGKRAQVCGGD

>Ensembl_Y23B4A.2_Cele

MLLWIVATLLIFSLPVSTALDYNDFSLQRIARAPHPSSALLVPYPRVGKRSNILNNNSESQNSVQKRLYMARVGKRAFFYTPRIGK

>Uniprot_NPY_Hsap

MLGNKRLGLSGLTLALSLLVCLGALAEAYPSKPDNPGEDAPAEDMARYYSALRHYINLITRQRYGKRSSPETLISDLLMRESTENVPRTRLEDPAMW

>Uniprot_PYY_Hsap

MVFVRRPWPALTTVLLALLVCLGALVDAYPIKPEAPREDASPEELNRYYASLRHYLNLVTRQRYGKRDGPDTLLSKTFFPDGEDRPVRSRSEGPDLW

>Uniprot_PAHO_Hsap

MAAARLCLSLLLLSTCVALLLQPLLGAQGAPLEPVYPGDNATPEQMAQYAADLRRYINMLTRPRYGKRHKEDTLAFSEWGSPHAAVPRELSPLDL

>jgi_108298_Bflo

MTSVRIFSYVCLLVLIYACVEVTRAQEEEDVEAPEEGKYYKNLANYLRLLTRQRYGRRSAPDYGSRTLYRHPPPYPQDIVHTLDMDGISLSLCISSLNL

>jgi_187115101_Skow

MDLKTILLLASLVCMVVCWDERLSARVQRDASDYQAPTAPSRGASLAEWDRYLRELSLYRQYADIQRFGRSDGGANNIHQQTQKNHRSNIWDYLSKKLALE

>jgi_204449_Ctel

MKLSQILILLLLGLSVIICSVHANTDDDDARLVMVMSQMHPPKRPEHFRNMDELNVYLDKLRQYYTILGRPRFGRRNVFSEVRVHTIVLYCFCQSRLAPIRCA

>jgi_204022_Ctel

MDAKLLLKAMFVLASCLLLLLPTSCSQDMHGMPVRPKVFRNADELRMYLKALNEYFAIVGRPRFGKRSSPSENPAFGEVDVDGNGRVNDDEFDQFAYGYRK

>jgi_229397_Ctel

MQKLVLSLLILAAVVMLEVTSQDSMLAPPDRPSEFRSPDELRRYLKALNEYYAIVGRPRFGRSVNKRSAADYLFKPSHKNDYEYLYDERDERFPSPYY

>jgi_103536_Dpul

MSSSNNSIQQFLPRSCSLAALVFLVVMAVLAVCVTTTKADGGDVMSGGEGGEMTAMADAIKYLQGLRRYDNSLVRPRFGKRGRLPYFDMEDLPLHPQVCSSSSYF

>Ensembl_FBtr0083328_NPY_Dmel

MCQTMRCILVACVALALLAAGCRVEASNSRPPRKNDVNTMADAYKFLQDLDTYYGDRARVRFGKRGSLMDILRNHEMDNINLGKNANNGGEFARGFNEEEIF

>Uniprot_CRH_Hsap

MRLPLLVSAGVLLVALLPCPPCRALLSRGPVPGARQAPQHPQPLDFFQPPPQSEQPQQPQARPVLLRMGEEYFLRLGNLNKSPAAPLSPASSLLAGGSGSRPSPEQATANFFRVLLQQLLLPRRSLDSPAALAERGARNALGGHQEAPERERRSEEPPISLDLTFHLLREVLEMARAEQLAQQAHSNRKLMEIIGK

>gi_62911756_Sauvagine_Psau

MNLPSSVALLACFLVLLDRSELAPLERSFFHESGPEESARSLLDSDPMKRQGPPISIDLSLELLRKMIEIEKQEKEKQQAANNRLLLDTIGK

>Uniprot_UCN1_Hsap

MRQAGRAALLAALLLLVQLCPGSSQRSPEAAGVQDPSLRWSPGARNQGGGARALLLLLAERFPRRAGPGRLGLGTAGERPRRDNPSLSIDLTFHLLRTLLELARTQSQRERAEQNRIIFDSVGK

>Uniprot_UCN2_Hsap

MTRCALLLLMVLMLGRVLVVPVTPIPTFQLRPQNSPQTTPRPAASESPSAAPTWPWAAQSHCSPTRHPGSRIVLSLDVPIGLLQILLEQARARAAREQATTNARILARVGHC

>Uniprot_UCN3_Hsap

MLMPVHFLLLLLLLLGGPRTGLPHKFYKAKPIFSCLNTALSEAEKGQWEDASLLSKRSFHYLRSRDASSGEEEEGKEKKTFPISGARGGARGTRYRYVSQAQPRGKPRQDTAKSPHRTKFTLSLDVPTNIMNLLFNIAKAKNLRAQAAANAHLMAQIGRKK

>Ensembl_ENSTRUP00000030463_CRH2_Trub

MRPTPLLLLLSALLLSSLLRPAAGRPSSLPSWPDELGRLRTQQLEEMLLQAAAAGDSAASELLDDLLLKLFQRRSPELLHLLPVGQEEEAARPVLKRSEEPPLSIDLTFHLLRKMIKEAKMENQKEQELINRKLLDEFGK

>Ensembl_ENSTRUP00000040554_UCN_Trub

MLSSLKTLLLLSVLCTPTSSLCLHLSDYRLPLLCDDHMAVGLPGDDDVPGYSPADDWASLLQSAYLSSSSAESSREKRTAAPANYRFLRRTKLRGQMLRNSSKGDRRSRLTLSLDVPTNIMNVLFDVAKAKNLRAKAAENARLLAHIGRRK

>Ghost_KH.C4.329.v1.A.ND1-1_Cint

MVLVTVVVFVVLAMSLGVQLVDGKFHVGTSSPLLLDGVALGSLTITDYKEEYAAPAVNRKTRRSPSCCGLHAIPRVKNSIFIQPNRSTTVHLTTRKPALGRNPFLSLDVPLSFLSKLMEVKRREEQRKIAKIKSVHNSSLMKRIGK

>Ensembl_reftig_15_538821_539886_Csav

MENSFVCFAVFALVLSRLAGAKSDKLGLSQKIVAIKDDWVVRCAKMQSEIANPLNSWTKRGISLEKTTKVDPISGTDRSPRFRRAAKPKPQTNVLSLDVPLSFLSKLIEMSRREDKRQREKEISLRNKKLMEKYGK

>gi_187098484_Skow

MHLCIVIIRFMAVIFVALTVVEANPIHRPSMLSEWLRTLKDENDRDTNIGYREITAFKRNPTSGHSIGLSLDIIRDRLEKAESERMQQEKQSDINRYRQNTELMTGLGRRRRSMEEVYRQ

>gi_155729_Egg-laying_hormone_Acal

MKRPNNRPTNTMSLILCLTLSSLCVMSQSASVHGKNFATNRAVKSSSPFVVLSPDDNVVSMSGENGYRSALREAFDKSSRDYDDNGEDVFSNEKRRLRFHKRRLRFDRRDQDEGNFRRFPTNAVSMSADENSPFDLSNEDGAVYQRDLRAPRLRFYSLRKRAAGEMEQSEGQNPETESHSRRKRSVLTPSLSSLGESLESGISKRISINQDLKAITDMLLTEQIRERQRYLADLRPRLLEKGKRSSGVSLLTSNKDEEQRELLKAISNLLD

>jgi_169597_Lgig

MTSYAMPRSYSQVPEELSSQDQSRDYYEPLNAILHRLRELILMQPHSLRETIRYTQITPTTPIPIAMKKRSRLSINQELKSLANLLVLRENKRREAQKTKLRSKLLSIGKRSMPQNQDRDHVIEETPVSDRDMFLTELFAEISEQEMTYVLGQVLGSEIAAKGGNEYQGQERLPYEI

>jgi_sca_87:656961-669122_Lgig

MEWNMNKSLSVLVLCLFIFVVDDAHSYSMNRKSESSRHIRSASDIPDDRIQDSKASFHENYFGTDPDHQNLYRERLESMFSDQKKRMDIHQQQVSLKNLINLIHETSNRIRKLSKTERPDMDDDENVRIKTVVKTWSKNDPENGRDNVISGYKTEVGNPKRAGRLSINGALSSLADLLVSENQRRDRLESMELRQRLQYLG

>gi_260927617_Cgig

MKMKLNVMAVIVTALFVSVDAFILQDQDNQPDLSEADSIPVEKRGRLSLTADLRSLARMLEAHRKRFIASRFPYDSIRKKLFRYGKRSPVTETLEYNEGEDRSNALDSEFPSVKVEEESPIYNIKRQRLSVNGALSSLADMLAANGRQRMMSEMAMNRQRLFGLG

>gi_144925239_Lymnaea_stagnalis

MKMSGLLSKPDYGVVGIVFTVVFCCWCSSSTTHALSIAEPGRDRYDKRSPTGHGVEVVEVAQSGEDYGSNRPQPVYGDEDEEDSADVYVGSDESSSGEKTRLTAAKRRLRFNKRRLRASKRRLRFHKRRVDSADESNDDGFDRKAREPRLRFHDVRKRSVTAEEGSENAEIEESHLGNSRSRRSADSAPSSANEVQRFKRLSITNDLRAIADSYLYDQHKLRERQEENLRRRFLELGKRGSAFFDHIPIIFGEPQYDYQPFK

>gi_213134317_Hmel

MMWWAIWCAVVVAGSAGTHAAPSSNMIPSAIDWTQFDALAPEDDSLGYAVSPMSRRSAAGSPRLYLLAEVPRDSQNLAELREGRPRPKRKMPPLSINNPMEVLRQRLLLEVARKQMREANQRQAVANRVFLQNVGKRGFWAGSAPRYDN

>Ensembl_FBtr0082080_DH44_Dmel

MMKATAWFCPVLLTLLCATRLVCTAQRGAVGAGGAAGGSGAAAGGAEVGGSGRTNGYPLDYPDGTRNSQDDFLLAKRNKPSLSIVNPLDVLRQRLLLEIARRQMKENSRQVELNRAILKNVGKRVVLRGGGGGGGSGAGGLAPKVSRRYRQQWPVERELERERQRERERERDAVREEQLDRQQLLPWKHFPSQLWSYGWALSPYKESSQLQFADSQQSASTGPQSQALPKQLQLLSYAKKPLDVAGMSLARHRVSGNEANETNHENDDGNGASKNPARYVDDGDNEGEDSYNDVGTEGVGLGLGMGVGLGLERFEVLEDKPNWANEEPNELVVVNANDRVPWSFPYRFHKSQHNVN

>jgi_95468_Dpul

MRWHVATLAAVWLIIMGWCGSAVMGQPIAGSMDQPHPAHHPPAGQTIPEMWALMDPADGPPDTIRDGDDLNGDLYYFPAVPIMKRQQSLDDDSGWISQDRSKRQSQSHSGSSGNHHSQLSIISPIEALRSRLRLEMLRRQYGNQIKQNQDKLERVGKRRKRSDQSSPIAVDGSDEKRMSQPSVAPAAIKAP

>gi_125487989_Isca

MRCAQSSHPLLRPWLLLALLVTDAALGARLGGSKASLDLSDLWKRGPLSLRSRGPVPSLSMHSRDHMPSLSIVSPLDVLRDKMMQDIIERSIKNKIQANDKILKDLGKRSAPPAAIGEDVAVSLLRRDDDRSSFSPQESFSDELL

>gi_300043228_Apis

MRILTIAWILVAVTWCCDSAVIYDPVVQNTRYYEPRIQDLELLDKNFFDMASIEKRNGAMQGESPRSRPSLSIVNSLDVLRQKLMYEVARRHVDENQKVLSQNHQILKNLGKRSLFPFIEVPRRYNVFRI

>gi_282199930_(partial)_Turt

TDGYLQNLLNEYRQKQGSINLRHWVRGVSPPRLSVTSPIAVLRDALMEEIKRKKIQETQAKIALNEKILKEVG

>gi_164433160_Oarc

MIECPQKTFNGINNLVNLRRSSKFSWTLISLILMITLACVLESSTVVSSPIKSKSRRDHSHHHHHHHHHHRQSRNHTKHHRIPEILEQPVPDSSGSLSIVGPLDVLRQNMLMEMHKTRVRHAKNKIVESNREYLERIGR

>Uniprot_CGRP1_Hsap

MGFQKFSPFLALSILVLLQAGSLHAAPFRSALESSPADPATLSEDEARLLLAALVQNYVQMKASELEQEQEREGSRIIAQKRACDTATCVTHRLAGLLSRSGGVVKNNFVPTNVGSKAFGRRRRDLQA

>Uniprot_Calc_Hsap

MGFQKFSPFLALSILVLLQAGSLHAAPFRSALESSPADPATLSEDEARLLLAALVQNYVQMKASELEQEQEREGSSLDSPRSKRCGNLSTCMLGTYTQDFNKFHTFPQTAIGVGAPGKKRDMSSDLERDHRPHVSMPQNAN

>Uniprot_IAPP_Hsap

MGILKLQVFLIVLSVALNHLKATPIESHQVEKRKCNTATCATQRLANFLVHSSNNFGAILSSTNVGSNTYGKRNAVEVLKREPLNYLPL

>Uniprot_ADML_Hsap

MKLVSVALMYLGSLAFLGADTARLDVASEFRKKWNKWALSRGKRELRMSSSYPTGLADVKAGPAQTLIRPQDMKGASRSPEDSSPDAARIRVKRYRQSMNNFQGLRSFGCRFGTCTVQKLAHQIYQFTDKDKDNVAPRSKISPQGYGRRRRRSLPEAGPGRTLVSSKPQAHGAPAPPSGSAPHFL

>Uniprot_ADM2_Hsap

MARIPTAALGCISLLCLQLPGSLSRSLGGDPRPVKPREPPARSPSSSLQPRHPAPRPVVWKLHRALQAQRGAGLAPVMGQPLRDGGRQHSGPRRHSGPRRTQAQLLRVGCVLGTCQVQNLSHRLWQLMGPAGRQDSAPVDPSSPHSYG

>Ghost_KH.C7.399.v1.A.SL1-1_Cint

MVMNTFCSLLFILLVFCVYNASTTGGIYNRLLHPRGTGRQQLLYHNFNNRYGEVLKRNQPSRSDYDTHDELIKNVQPLQQRQEEKLNKLEKLFADIMLEDNSYKNVLFSKKQESNEIDIEPSMMRDTRNNFLEMLQLSNWIHRVLSRLAATPHEQQAAPVTGKRCDGVSTCWLHELGNSVHATAAGKQNVGFGPGRK

>jgi_87904_Bflo

MMTTEVGRFLLCACVTFSILLTTCTAAPTARGELPEASLRLSEEDVGRVVSAMAREVLQIVNENVDKLAQPNEFGYGLDGLPQGPVYKRGKIACKTAWCMNNRLSHNLSSLDNPTDTGVGAPGRKRRDTS

>jgi_87907_Bflo

MLLGNMLVNTVAALTVCVVVFWGTDHVGALPRPADDGDNPLAMTNSDMRRIVSALIREVDQLAAEKLAAQRVFGPGIRKRDCSTLTCFNQKLAHELAMDNQRTDTANPYSPGRKRRSAEEDDH

>jgi_87911_Bflo

MRPCFALMVLAFFVLVHTNQLTASVIRRYDDDMYDYDGDMEPLLALLGQQAGRVYHGLARELLDNIEDNTNTQDNRFSRTYEDMMDPMAMRVRRKCESGTCVQMHLADRLRLGLGHNMFTNTGPESPGRKKRSLGASRRAARRL

>gi_115767208_Spur

MKSTVIVTLTICCLLYQTTRAASLTNRDGLSRQDILDLLQLYEEPIRQEGGDKRSKGCGSFSGCMQMEVAKNRVAALLRNSNAHLFGLNGPGKRRRSVDDLPQVNDAETE

>gi_187217193_Skow

MDRLIVASSAFLVILYITCFVDVTIAYDDSESEEVVPPNYWSLQLAELTELVKLGEEVLAQKASAEMKAAKRGRGTSACGGFATCKQLEYGRKYATSKADLSHFGATSPGRKRRTTNSELDKQA

>gi_161220966_Ctel

MYVQVLSLLLGCVLLGGRISEAADERESEDLQEALRVYQNLGETRKGLTALKRVVSDLDGDILQKHDFHDLARKRKGLDILRRILSEMEADLIYEQKRTCQFNLGGHCATESAASVADHWHYLNSPLSPGRKRRDTGLYKAVVSGRIFKDSKH

>jgi_215078_Ctel

MVSQFLIESELSPRVLSIMNSAVMRFSLIAMASCIVIASLTSRAQATSLHRQRRDVTEPSLAIAVLEDLIMELRDEIHSKKKRRFDAGYGSRYGVAQSVGSKLMALKQAADWNGPGRKRREAEEEA

>gi_163526287_Lgig

MISTTPLYVTVSICLFYLTTTVYCRTDFRFLMKRDTRNNINTDSLQETLKNLDDEYQRLQKRTCAFGINSHQCTLTSLNNKMMSQAWLSDGMSPGKRSDLPNLTPDGRTQRLLDEMSNTKALLTVRDILTQADSPTARKRSCSLRLGGMCLTENLNAAANQYEYLSSGLSPGRKRRSLRHILLNRXH

>jgi_166645_Lgig

MGASILLGLTILLSYVNQVHLQMFHPRDISRGGQQQEEQLRRGYLIQILKSFLNRRQEDATEKRKRSCNLNLGFHCQTDEYSSIADMYDFLQSALSPGKRKRNVKIVSIEGS

>jgi_299999_Lgig

MSRFVMTIFFLLVACLALIVPGSAAPPRRPMLVDLDDPDSVMEVITRLERSLLRNSDYEHQKRGVDFGLGRGYSGSQAAKHLMGLAAANYAIGPGRKRRDTTESTPEDVKTGAIN

>Ensembl_FBtr0079699_DH31_Dmel

MTNRCACFALAFLLFCLLAISSIEAAPMPRYQSNGGYGGAGYNELEEVPDDLLMELMTRFGRTIIRARNDLENSKRTVDFGLARGYSGTQEAKHRMGLAAANFAGGPGRRRRSETDV

>Ensembl_ZK287.3_Cele

MSCSSSSMLFLVLIATTVLIAESRVFYNRFDGGLSSDRFMEQKRDGAEASYDYDANQVIRNTMKRNRQCLLNAGLSQGCDFSDLLHAQTQARKFMSFAGPGK

>Uniprot_HCRT_Hsap

MNLPSTKVSWAAVTLLLLLLLLPPALLSSGAAAQPLPDCCRQKTCSCRLYELLHGAGNHAAGILTLGKRRSGPPGLQGRLQRLLQASGNHAAGILTMGRRAGAEPAPRPCLGRRCSAPAAASVAPGGQSGI

>Ensembl_ENSDART00000104549_Drer

MDCTAKKLQVLVFMALLAHLARDVEGVASCCARAPGSCKLYEMLCRAGRRNDSSVARHLVHLNNDAAVGILTLGKRKVGESRVHDRLQQLLHNSRNQAAGILTMGKRLEEPAKFLIPTVPQDVDSYEKR

>jgi_66903_Bflo

MRCMHVLLAVLFVGLLCKGSWAFPRCCWRRTCNCPHFRRLFGPRNHGHGILTVGKRQRGEGEYKSLPRGEHDPSHGGLPTSLVDLHDRAQVDLDRLEGLPELTLDHDYRQMRCMHVLLAVLFVGLLCKGSWAFPRCCWRRTCNCPHFRRLFGPRNHGHGILTVGKRQRGEGEYKSLPRGEHDPSHGGLPTSLVDLHDRAQVDLDRLEGLPELTLDHDYSERKNQLEHSKVWELLHHLRRLLNLTAEADARSRRRQTELQPYETTPRHWQISMRR

>jgi_scaffold_1000_Bflo

MAGRQVAVAVLVVAMLFRCRAQKPACCESGSCICPYVSRLHSPGNHGYGILTTGKRQWRHGPATLSPDQLVTASHGQGVPVLEGSDR

>jgi_Contig31345_Skow

MRTTIMLIFSAVLVAVLFTHTSAQPQCCRGVGCKIPPNCKCPFQSIICDNPTKNVLTAGKRNYLPISKSNELSTYQRPVSDAKTNVVTSDDIVNLIRSSPSLIRKIVKAIDLNGDDMISKAELQSLVYD

>BCM_Contig9884_Spur

MRLERLCSIVVLVIAACCLLVRGQSPCCRRAKGCSFPPGCHCPLKMSFCGDPSRGLQIVGKKSDTSYTGGYSTTNLQKYDYHPADREYVKHDR

>BCM_Contig134164_Spur

MNRGVPSQIPRVLLAISLVLLVCLPEISHADRACCKRTVGCNLRSDCTCRIREITCTDPSLGLQNYGKRSPARSPTYYDSYTPILERIAELASLQQDDRWKRKRSSSSSSRYPFYDYNMRPF

>gi_223023872_Mgal

MRLCGLLALISVSIVIVDSSPALSRHKRGFLAGVHDRMGHGFGKRTDPILSSYVDDVDKDDLMTVEDLVRHIMQSEVLADAIVRKFIDINDDGSVSYQELLRKLMR

>gi_148318561_Lsta

MSRTSLTLQVGVVLLAICLFDITYADERIHRQKRGFRANSASRVAHGYGKRGYLSSNENLPTLSLDQLESSTGLMEEISDGSLMTVNEFSQLLTSHPNLARALVKKFVDINGDDVISTDELFRPILKK

>gi_121313125_Acal

MLSAPSIAHTGVALLVLMCLCPFSQSTEASLSRAKRGFRLNSASRVAHGYGKRGYASSSGAVPYPELARDVLDNLRAEEEEKELEWSIMSVDELASLLQSHPKLARALVKKFVDINGDNLVTAEELFRPPTRK

>gi_163522278_Lgig

MKLTLVLVTVTLTITILANAYPQSPSLSHHRSKRGFKANSASRVAHGYGKRGFPSWKNYFQDGGSDVLSVEDMAELVAENPSLAKALLRKFVDTDNDGIISTTELLGTKQMLK

>jgi_226681_Ctel

MKVSICFIVVALVVCIEVMTSHAANLSRSKRGFRMGAADRFSHGFGKRGGDFNSLIDGESDMVMSDEDLTEIIRADARLAQTFVKRFIDTDGDGFVSRQELFEA

>gi_223784927_Apom

MKSILCLSVAALMLVAVSGVSSKLTVTGNSRRVARGFRLGAADRFSHGFGKRTDDHLPEVIPGAESDVLLSNAEFAELLRSDEKLANLIVQDFVDKNGDGFITRNELMGYRK

>gi_160609080_Hrob

MKTMKLLAASFLLVICTNWAFDVQRYPTKYKELNEPQQQQPASNDNDVGNNDDDNDVPSFDQQQQQQQQQQHHQQQQEYQIKRRSFKLYDNSRYAHGYGKRTNPLLAPSARMTSHEDWHADGGWGF

>gi_112983783_AT_Bmor

MNLTMQLEVIVAVCLVLAEGAPDVRLVRTKQQRPTRGFKNVEMMTARGFGRRDRPHPRAELYGLDNFWVMSEPSPEREVQEVDEKTFESIPLDWFVNEMLNNPDFARFVVEKFIDLNQDGMLSSEELRNV

>gi_240248407_Rpro

MMRWSSLLVLVALASIINCIKAGSPSSALYSSAARASGRTRTIRGFKNVQLSTARGFGKRTYPDSQLQPDLIPADWMAEELSSNPELARFIIRRFIDVDQDGLVSPVELLRNTVCQEPN

>BCM_Contig45963_Apis

MAVNNNIMVRLLVIEITFLILAVVNSYPAFEDSEFKHKHRDKGRTIRGFKNMDLSTARGFGKRTDHYMNLMPLDLFVDNKEDSFNQNIPMEVSLEKILKNKYKHFIEKLIDVNHDGYISGEELLLSIDGES

>jgi_255977_Dpul

MKGKGAFLMVLAGWGLIGLMILTTAVEAAPHPADYTSSSVNNQRDFRSRRGFKTVGLATARGFGKRAPSLSNFNSFQDAAEQMMQQQEENPNSDPDVNGKV

>jgi_50560525_Homalodisca_vitripennis

MRVYTLLLWLVVLLMSVTANSVPVYLRDKPRSIRGFKNHALSTARGFGKRSDSELSSSETSQFTDRDSFPADWFASEVQNNGELARLVVHKFIDTNQDGELSADELLRPLYGPPTTTYK

>Uniprot_NPS_Hsap

MISSVKLNLILVLSLSTMHVFWCYPVPSSKVSGKSDYFLILLNSCPTRLDRSKELAFLKPILEKMFVKRSFRNGVGTGMKKTSFQRAKS

>Ensembl_ENSMODT00000042215_Mdom

MTSSVRLNLFLVLWISSMRMLCCYPTPSAMLSEKSDYFLILLNSCLAAMDRSEELAFLKPFLVKNAMKRSFRNGVGSGMKKTSFRRAKS

>Ensembl_ENSGALT00000041356_Ggal

MISLCRLNLLFILWMSAMFVCSGYPVGPSMNCQLYGKSDYCLVLLNSCLAKVGRSEEVALLEPHLEMPFNKRSFRNGVGSGIKKTSFRRAKS

>jgi_84803_Bflo

MMQTPIFLCSVVLVGAVCGQLSETNNFPQNGNRRLSPERSATVLRQFLHLEGAVGSPVSPSDGRALETGDKRSFRNGVGKRRDSEEERLPQNRGATELKAEATIFSQNGDPHDEGAKAASEKRSFRNGVGKRTHFRIVADASLGGLDEPEALRQTGGDANSPSLSRDLWAEVQGKDDEQCPACGSDGSGVCVLKGVCCRLDSGCVLRKDVCSSLPDRALCASLQYSATCRTDGKCVAPGVCCRAADHSCFLDPECD

>gi_390337096_Spur

MGYERRILRTLLSILIVLASFVTVYGERDSNFMQHKLFRNIVPSPLIQKWRENRMGPAEEKTSNEQWRDELLSNLRNVLRKHNASPSSRSIDRSDITGYGLQEPMQQLPADVTADQLFILEGAVNSPTENYEEETPIDEDKRNGFFFGKRNGFFFGKRSDSDASSTKMDDDRLPKYKSSGSFDKCRPCGPGRQGRCVMVGTCCSPLFGCYLFTPEAAACMTEDVSPCQLNAPSCGLAGKCVADGICCSAAEGACHLDPTCTSMSLN

>jgi_Contig31671_Elphick2012_paper_Skow

MLRKINAVVFFLVAICTLSRATFGEDGMTEKQVLKLHKYWPKEDISELGSSSTSGDSGENEAVKMGFWAVDGKRNGFWNGKRNGFWNGKRNFDDLRIFGTNKKHSSINNQKRNGFWNGKRNFDDFEIPEKKEQPHWRDEKKNGFWNDGKRNFDNFKTDDLQYPSIEDKRNGFWNGKRNFEMSMNKKASASTESEKRNGFYMGKRSVDNTMNISSYHYKESAKKCTTCGPGGKGQCVMYGVCCSLEIGCSMLTKETEECTTSPLVGECGRSDVQCGNFFRCVANGVCCTKETQSCKIDQECNVRW

>jgi_195692_Ctel

MNSQWVTSSLLALLLITLLCSMASTCAQEIPQDSMKKRIFCNFDGCYNKRAEAPQKSNTDLDVLMRVYKLLQSRAGGKPSSETNTIVDAEYQPNIDGPRNLLRQLHTIMRDIENN

>jgi_163724_Lgig

MICSHFHCLLLASFIVLTVSEDFSPLYLNENEDTNDLLKDTDSLPSSNNIYDSIPPARPPFPLLSLLFSKLKMEGSEKQAVIKRVFCNGFTGCGGRHRGRRRQYTARILKRPFCNNWGCGNGKRALEKVPVQPFGKNLARKRLFCNNYGGCRGGKKRTLYSNWLGKLNGVADNL

>gi_260739332_Cgig

MVSFQQSFATCVGILTILSFCQCSQYSYADELLQKNENSKLGVKKLAALLANELLSEDKDITQKDKRVFCNGFFGCSNGKKRSNSMNLNPLDYPVDAPEPETKTDFRKRLFCNTGGCFGRRKRSTSQTLERRLNNRGSKDGNQ

>gi_121425289_Acal

MTSSVLSPTSSFLLVLVLNVMLASIPLSAVTADEFFKQAAESKTTVKRSTSGESKPPRLLELIEMRDAVSSMLPVDDTPSLTRNFDGTSGVGDGLQARGSQVPRLFKRVFCNGFTGCGGRFRGRRRQLNEALGKRRLSQQRVGKRPFCNTLGCYNGGRKRHEEE

>gi_117948139_Ipar

MTMMHSTPSCVPVGFFLQPVYVLLICTLVQAASSQAQESTSQIKSSESNDLSMKIRHLILRALEQQQQQQQQQAAAADGYTDQEDIALPPYKRVFCNSFGGCRSLKRLFNDEPGSLNPASEDSVQPTGQEADIEKRVFCNSYGGCKSFKRVSANAMKNPALSKNRQR

>jgi_300598_Dpul

MTRPLFYSLLMLAWMIISLYISASSSQPLKNNQNDSDSAEEIEQWSFKEKRPFCNAFAGCGRKRSMIKDTKHPPYEKAASNHPRLPNADTKLLDKLFAKIQHQRANFVQLDDPEYY

>FBtr0084333_CCAP_Dmel

MRTSMRISLRLLALLACAICSQASLERENNEGTNMANHKLSGVIQWKYEKRPFCNAFTGCGRKRTYPSYPPFSLFKRNEVEEKPYNNEYLSEGLSDLIDINAEPAVENVQKQIMSQAKIFEAIKEASKEIFRQKNKQKMLQNEKEMQQLEERESK

>Uniprot_RFRP_Hsap

MEIISSKLFILLTLATSSLLTSNIFCADELVISNLHSKENYDKYSEPRGYPKGERSLNFEELKDWGPKNVIKMSTPAVNKMPHSFANLPLRFGRNVQEERSAGATANLPLRSGRNMEVSLVRRVPNLPQRFGRTTTAKSVCRMLSDLCQGSMHSPCANDLFYSMTCQHQEIQNPDQKQSRRLLFKKIDDAELKQEK

>Uniprot_NPFF_Hsap

MDSRQAAALLVLLLLIDGGCAEGPGGQQEDQLSAEEDSEPLPPQDAQTSGSLLHYLLQAMERPGRSQAFLFQPQRFGRNTQGSWRNEWLSPRAGEGLNSQFWSLAAPQRFGKK

>Ensembl_ENSMUSP00000023814_Mmus

MDSKWAALLLLLLLLLNWGHTEEAGSWGEDQVFAGEDKGPHPPQYAHIPDRIQTPGSLFRVLLQAMDTPRRSPAFLFQPQRFGRSAWGSWSKEQLNPQARQFWSLAAPQRFGKK

>Ensembl_ENSORLP00000006177_Olat

MDTAAAMTLLALIVALAGISQALHVQDSLGKNDILPGSSEENVADRLLRLRETRESVLHQPQRFGRSSRGQVVSDDQIQARDWEAAPGQIWSMAVPQRFGKK

>Ensembl_ENSDART00000066179_Danio_rerio

MDAGVWFMLVGVLLVMADQSRSITQDEALEQNKRLLEEDEEQDNMLERPGAAQMNGLLEDRLLVEMLRSLLHGSQRYERNPSVLHQPQRFGRGARSGLSTEERIQSRDWETVPGQIWSMAVPQRFGKK

>jgi_596067_Trub

MDTSALVTLLALVVAMTSVSRALHIQDVLDKEDLPGSEEKMADSMLEMRETRNSVLHQPQRFGRNSKGQVMLENEIHPRDWEGAPGQVWSMAVPQRFGRK

>Ensembl_ENSPMAP00000004221 _Pmar

MEAKAVSAMLLLALANCVLVSAARGSFSSMEEAAMPDSDSSLTKDYLAESVHEDPYRDSFDRASPDAAGSSSSEQLLLSRLARAFMHFPQRFGRAGPSSLFQPQRFGRGSNDDEEVPPSLFYRRSWGAPAEKFWMRAMPQRFGRKK

>NPFF_AMPHIOXUS_Brafl1_112219

MGIFALMVVTLVVSQSGATPSSSEWKRSLYHFVSPEESDGSNEFPSHQRFTLRPDSSDESEAERVDRMSRAAAASLFRPPNRFGRSPAVWLDSPNRFGRSARFSVPFDIPRRFGRGFSLDSDVPRRFGRAGEEETPKRSWSMLSFQRPFRFGRAIATSNV

>jgi_176362_Lgig

MVAKGTVGILIVLFNTICANIDLNYNQVPVANPLLFGRRGINPDMSSLFFGKRSGNSDHRDLRKMKDTCKAVLSSCKILFSDYEDDTVRNKVQDGFGRFK

>gi_207118044_Hasi

MNCKIPCLLLVLTFCIMSFTHAQANPRNLNRLVGQQPLLFGRRGVNPNMNSLFFGKRAPSMNNMDVRTMCNAVLSACAAWQADMTDN

>gi_260928914_Cgig

MKIYSIISIVIALVAVIVLKTSASKENSRGLTRLVGQQPLLFGRRGMNPNMNSLFFGKRAVDRPTLDDIIVEKCSRIMAACREYAHERMGEDDI

>gi_117945496_Ipar

MSGRRNLLVPASLVLILSAILFFGHETQQVEAAYSPTRGQQNTHSFGRRGLNPNVNSLFFGKRGGSEQEALSNTEMGRKCLAAMSMCNMYFETNTINES

>jgi_175046_Lgig

MNRCTACLVLVLVVILTVNAVQSARGNGRQRPQLFGRHGVRPGMNGLVFGKRNSESQEMQQDCWSSLNLCLKVILNNGDMEEV

>gi_161198869_Ctel

MDCRLVTFVALFCSMLLVQQVLSDPLEDHLPETSGLFFGKRSSHPNMNNLLFGRRSYAQMAANYQVEEARQGICKSMKETCGKWGFDVEN

>gi_301553931_Apom

MQTKIVISACILAAMLVFQCACADPLRDQLPDTTGLFFGKRGSHPNMNNLLFGRRSYPIEDLEDARKVCRAVQATCARWGLDEN

>gi_326351257_Ecom

MDIKIVILACSLTVLLLSRQASTEPLEDQLPEDTGLFFGKRTSHNPNMNNLVFGRRSYQPKVQRRMNAEDAKRICLNVRLTCAKWFAEEDQMRNLKH

>BCM_GLEAN_08645_Tcas

MQLALAKVFSVCIVVIILTSWIEMTEATYRKPPFNGSIFGKRGATIGKLLTFVIFLSKIISSEYDSASKALSAMCEIASEACQTWFPSQEK

>gi_118778560_Agam

MAAFKVLGSLIVVLLVVLALSGHAEAGYRKPPFNGSIFGKRNGNSVDYEGNAKVLSTMCEIAAEACQSWFTQEQK

>Ensembl_FBtr0091490_IFa_Dmel

MALRFTLTLLLVTILVAAILLGSSEAAYRKPPFNGSIFGKRNSLGKSKIRIPLKPPPISPSRLRQRQNERRLRGGHGGVSHVVSPERQQIGPRPATPPPRTDLEPTTNTPATGGQMLCLLVRLNVEMPDVKKVMYKIYNVSRAYRYIELMPYIYIKYSINLQH

>jgi_260818_Dpul

MRSSFIVVMVCVVVVLTFWGQVAEATRKLPFNGSIFGKRSNQGTDKLESPSNLQLLCDAAMNACSDWLPIGSK

>Ensembl_F37A8.4_Cele

MWYIALLLAVIATSVTAQKADDEPIVFLVRVPIDEMDDDSSLLESYYHPRDILSKRAIPFNGGMYGKRSTMPFSGGMYGKRSGQIFAQRRAAIPFSGGMYGKRSLVPQSYSNNENQIKRGAMPFSGGMYGR

>MPI_GENEPREDICTION_JIGTRA00000189416_Ppac

MTFSGGMYGKRAAPSQFSGGMYGKRSETNYKRGPVMFSGGMYGKRAAPSQFSGGMYGKRAVPMTFSGGMYGKRASSPMMFGGGMYGKRAPMPYSGGMYGR

>Uniprot_NMB_Hsap

MARRAGGARMFGSLLLFALLAAGVAPLSWDLPEPRSRASKIRVHSRGNLWATGHFMGKKSLEPSSPSPLGTAPHTSLRDQRLQLSHDLLGILLLKKALGVSLSRPAPQIQYRRLLVQILQK

>Ensembl_ENSDART00000098361_Drer

MADDGHCKYTLLFALIMLFNVSLSTSVSLDLTELRNKVSKIKVHPRGNLWATGHFMGKKSISNSQLQDSPFPVKPDRNIMGESGSSEDLKELITQEMLKIALQAQLEDPKRTRDVYNQVIREIFKELANRM

>Ensembl_ENSDART00000112743_ZEBRAFISH

MSSPSVSRFCCCGFLTYLVLFSFYISKTSSVSLDLTELRNKVAKIKVNPRGNLWATGHFMGKKSVVDSKHLPTEDESTMTAVEAALNARQVEPEDVFQEMLRVALQTHLDTRHIRPNVPETAILMKILESYIQDNK

>GRP_HUMAN

MRGRELPLVLLALVLCLAPRGRAVPLPAGGGTVLTKMYPRGNHWAVGHLMGKKSTGESSSVSERGSLKQQLREYIRWEEAARNLLGLIEAKENRNHQPPQPKALGNQQPSWDSEDSSNFKDVGSKGKGSQREGRNPQLNQQ

>gi_432152184_Bvar

MSAIPLNRILPLGFLLIFSFISLSSCMEFVEDPNNQGGLNLQQRLGNQWAVGHLMGKKSLQDTDFEEMESFAKRNVENMKAESERELRHAQLVVRNILEQYLKNMQN

>jgi_93381_Bflo

MKSGWYVAFVLFVAGLLAPSRADKGQEHWQYGHWYGKRDPSDVNNAQIDDVLRSHPELQQLLNKLMELPRRPKASSQMYTKYDDDYPDGDVSDSTKRVLEEPGSPYWKRVNEHVDRDYTSIIQRRSAKNPRVVQGHKVLKPKTNSFKRTIWDRFMARIFPAGED

>gi_161278389_Ctel

MLSVRSVGVLVGIAVLFSLQSFVQAKCGGSWAIHACAGGNGKRSEGSAGARAPPALCRATRARRSTEATLPAIHREFCRGTSERPN

>gi_163502663_Lgig

MEVRCTTLFAVTSLLYLTISVSLVSGKCSGRWAIHACFGGNGKRSDPSLTDNTENSRQETLLRQILLPQTYEYHKSNDALLQDDDINTYEKSHEESQRMRDLRALNILLKTLMMEQKVRTENSVMA

>gi_203607807_Acal

MELTFGSSFTVALLSCLYISLITCPVVTAKCHGRWAIHACFGGNGKRSDPNMSPSTDELPPTLLRQLLVSDVQRLSRLLREPEAPVEDADLSPNPGFEPGHPNEQLMPRLAAQKPVD

>gi_260741759_Cgig

MRNPSSFVLFSATLCVYFTVQISSVSASKCKGPWANHMCFGGNGKRSWSPPVQEPEMNRKDDELGRTMLRNVLLKRLNTYPSMSSYYSDSQSFYPMGSDFTEEGDSMSRENELRQLLKEQILRKEMAALVGDDDVYE

>jgi_304056_Dpul

MHIFFYVIHVTAMLAIVSGNCNKYGNACFGAHGKRSDFKRTSAVDLSDQIWPVAANWNPTRPDEPIQERRQMKPLPALQLESVLVYNDIPRSAEHSRYLNQEDYNN

>gi_194306572_Bmor

MAQICLAVSIAVLLMMSQGVSAKRGCSAFGHSCFGGHGKRSGEPAPMDMANQDMMVRHQLGQEETPPHPGYPHSSYNVLQPGDDIIPIRDGGVYDHDAAARDVMKYKLRNIFKHWMDNYRRSQNTNDEYFLETI

>BCM_GLEAN_00168_Tcas

MNCWSTQVVLLAFVMAFVLAAAEAKRGCATFGHSCYGGMGKRTENNNEELLQDVQSEENPAFVFTGPRSENQQKLTPEQYDNISRVIRQWILSYRRAQEMRPDYN

>gi_328778572_Apis

MAITSVNSQSVTVLIRTWIFMIIFCFAGGVAGSCLSYGHSCWGAHGKRNGGHNNGYLVPSKTINEQGVPYLTKDQFILSRLIGRPLVSNKYKGRWDRLFKIKASFPEHWNDDELDTHLINNEPIRDQNNNENMNQNKRKHIENVQNMAEYDNINDERKEIPEILLISNNENNHHDSKPQNAELFKFLSNTNDNFE

>gi_328778594_Apis

MKNNATSGLPLSVCASICALIILIFFTDNSYAKRGCSAFGHSCFGGHGKRFDPNIREKILQDDDTITNREIEDLNSRNEFDVSEKKFGGQTEILSSQSRHQDSSRFNPFALSFIVRQWLTSHRLHQPDMELNNK

>gi_27616677_Agam

MHQLSTICFTLVIVSVLVQSTNGKRGCAAFGHACYGGHGKRSGSSASTLYPDGLDPSMVGLEVLPIPYSKLALDKSRSMDSAPEGMRLTNVYGFDVPAASTHRTETRPGELKYAIYAMLRQLMDEAAINRQEQSHQQQQAQTLNQPPFPHEAASVSDIERK

>Ensembl_FBtr0113232_CCHa1_Dmel

MWYSKCSWTLVVLVALFALVTGSCLEYGHSCWGAHGKRSGGKAVIDAKQHPLPNSYGLDSVVEQLYNNNNNNQNNQDDDNNDDDSNRNTNANSANNIPLAAPAIISRRESEDRRIGGLKWAQLMRQHRYQLRQLQDQQQQGRGRGGQGQYDAAAESWRKLQQALQAQIDADNENYSGYELTK

>Ensembl_FBtr0303263_CCHa2_Dmel

MKSTISLLLVVICTVVLAAQQSQAKKGCQAYGHVCYGGHGKRSLSPGSGSGTGVGGGMGEAASGGQEPDYVRPNGLLPMMAPNEQVPLEGDFNDYPARQVLYKIMKSWFNRPRRPASRLGELDYPLANSAELNGVN

>Uniprot_Gala_Hsap

MARGSALLLASLLLAAALSASAGLWSPAKEKRGWTLNSAGYLLGPHAVGNHRSFSDKNGLTSKRELRPEDDMKPGSFDRSIPENNIMRTIIEFLSFLHLKEAGALDRLLDLPAAASSEDIERS

>GalP_Hsap

MAPPSVPLVLLLVLLLSLAETPASAPAHRGRGGWTLNSAGYLLGPVLHLPQMGDQDGKRETALEILDLWKAIDGLPYSHPPQPSKRNVMETFAKPEIGDLGMLSMKIPKEEDVLKS

>Ghost_KH.S618.6.v1.A.ND1-1_Cint

MNSFGRYTFSLAVVLYISIVLCAENSEAATAKRPFRGQGGWTLNSVGYNAGLGALRKLFEKRDGSSLDVESMPLDNEEMENLAKDFALFLEVKESGLLGPRMLRCILSRNDQVMDSMSEM

>jgi_325296814_Acal

MAHHRGHRHILLYVSLALSLGLALAEDATDPSDDTGSFDDVEAVSEEADLDPYSMSQELNKRPNVDPYSYLPSVGKRAFDHYGFTGGLGKRKIDHFGFVGGLGKRQIDPLGFSGGIGKRYDSFAYSAGLGKRGMDSLAFSGGLGKRGMDSLAFSGGLGKRGMDSLAFSGGLGKRGMDSLAFSGGLGKRGMDSLAFSGGLGKRGMDSLAFSGGLGKRGMDSFTFAPGLGKRGMDSLAFAGGLGKRMDGFAFAPGLGKRMDSFAFAPGLGKRGMDSLAFAGGLGKRMDSFAFAPGLGKRMDSFAFAPGLGKRGLDRYGFVGGLGKRGMDHFAFTGGLGKRDSGEASGDLEEGKRGLDAYSFTGALGKRGLDRYGFVGGLGKRGMDDFAFSPGLGKKRMDSFMFGSRLGKRGMDRFSFSGHLGKRKMDQFSFGPGLGKRGFDHYGFTGGIGKRGFDHYGFTGGIGKRQLDPMLFSGRLGKRSSSEQEEEDVRQVEKRSTTEEQSSKSL

>jgi_159314_Lgig

MAARKHELVLVLTSVLCFVSSIVGDPNVPSDSQDNSALTQDDFAKRGMDKFGFAGGVGKRGLDKFGFTGQLGKRDMDSFGFAGQLGKRGLDQYGFTGQLGKRGLDQYGFTGQLGKRGLDQYGFTGQLGKRGLDQYGFTGQLGKRGLDQYGFTGQLGKRGLDQYGFTGQLGKRGLDQYGFAGQLGKRGLDQYGFTGQLGKRGLDQYGFAGQLGKRGLDHYGFAGQLGKRGLDQYGFAGQLGKRGFDQFGFAGQLGKRGLDHYGFAGQLGKRGLDQLGFTGQLGKRQMDIFGYRGQLGKRQSIDKYSFLGAGIGKRSVKNTAGIKKDDA

>jgi_202196_Ctel

MSSNYHNNIQWLCPQIIPISFTGAEGELSAEDTQDKRAVDPAMKFAGLGKRDSDDSVDKRYLDPAVKFAGIGKKDMDPSMKFAGLGKREMDSFMKFAGLGKKAIDPWMKFAGLGKRYSNPSMKFAGIGKKAMDPSMKFMGLGKRYSNPSMKFAGLGKRYSNPSMKFSGIGKKAMDTSMKFAGLGKRYSNPSMKFAGLGKKAMDPSMKFAGIGKKSMDPSMKFAGLGKRYSNPSMQFAGIGKRSTDSFLQKMLEEGEKKRYQIDPGMMLAGIGKRISIDPGIKYFGLGKRSFDPTYSHIGLGKRSERTPLLDKDYHPVSDMENSWYDRSANLKRSYGRITKLRRQRLDPALRMIGIGK

>Ensembl_FBtr0084744_AstA_Dmel

MNSLHAHLLLLAVCCVGYIASSPVIGQDQRSGDSDADVLLAADEMADNGGDNIDKRVERYAFGLGRRAYMYTNGGPGMKRLPVYNFGLGKRSRPYSFGLGKRSDYDYDQDNEIDYRVPPANYLAAERAVRPGRQNKRTTRPQPFNFGLGRR

>gi_328721952_Apis

MHHSCCMWILVIATAVWTDAITGHEDKVGIKSQQAQQQQQSDIMQTMVDGGGHQSIQMTSPAESYFNDPLGPLGYLAKRAHKQYGFGLGKRLYRQYEFGLGKRSASKQYGFGLGKRAALKQYEFGLGKRASPTFYSFGLGRRASPQYSFGLGKRVSHPSFLNVDDRESDYTYNDLSEEKKRTADDMGHGQRFAFGLGKRGAGAEWDDGDGDGDDPAPILAPGPQAGSSSRRFPAGQKGQPQQ

>jgi_312277_Dpul

MLFLSTALFLLVALLQSSQCTDVNNDSSVIEAVGAGGGDSQAKAESLASAEDKLSALTADGMATRARYFKSFGGNPTGDPNLNIYSFGLGKRTSRSYSINPYSFGLGKRGGNAKSYPQQIPYSFGLGKRNPTKYNFGLGKRPDRFGFGLGKRNLKDDDLDQWLNDEEYSQFDDTEEEIEGREDDSQEIMDVNKRSQMAAGQQQQQQQQAFFPSHLQSAFYGGPFMSNLARANTHALGKSRTFNQQAATHDLPNLGKRLPVYNFGLGK

>Ensembl_T23E7.4_nlp-6_Cele

MLSFSRLAFVLLVSACVMAMAAPKQMVFGFGKRSMADISDMPEDVPMKRYKPRSFAMGFGKRAAMRSFNMGFGKRSAEPQIDVDSF

>MPI_JIGTRA00000185994_Ppac

MKTLLCLTILIAAALASLGDSWDDGDIEVARRSDPRMFSAAFGKRVDPRMFSNAFGKRSDPRLFSSAFGKRGDPRLFSAAFGKRGDPRMFSSAFGKRGDPRMFSSAFGKRSVQESEGYGPLNVVVRFSDGLKAESESADRNIRERRQTRQTRAALFETPPSLEFSSSEATTSVYSRFKERRELSESVSSSSTESTRSSSDREEEETIRPSLDLSSVAAAVTISSPNSSGEAAERSPATPSFLFDDYDQHIYTHMTMADFSIGDEVEEEIPSQLEDTEESDAVLHHKQATVREAHEMFVTVFGSCPLNELKAAVAPGRVNLIGEHVDYCDGFVLPMAIPLYTCAMGRRALDQDRGYSLIHSVHFTETVKIHKPYTEGKDKYPQWVRYVQGVFALLEADSLPYLDIVIHSHIPPVDRLSTTAAALLCQKAEHLYAGMPCGIMDQLVIAAARNQRALKIDCLTLDYETIPMSIAHDVVFLVTNSGVKHALATSEYAKRRSDVDRAVKLIGANSWRDVNEEILKQRSSSLTGEGDMMDRASHVVSEIARTVEAANALLDNNIILFGRLMTDSHESLRRKYSVSCSELDELVEIALSVEGVFGARMTGGGFGGCTVSMVRRDAVEDLKKEIKTIY

>Uniprot_TRH_Hsap

MPGPWLLLALALTLNLTGVPGGRAQPEAAQQEAVTAAEHPGLDDFLRQVERLLFLRENIQRLQGDQGEHSASQIFQSDWLSKRQHPGKREEEEEEGVEEEEEEEGGAVGPHKRQHPGRREDEASWSVDVTQHKRQHPGRRSPWLAYAVPKRQHPGRRLADPKAQRSWEEEEEEEEREEDLMPEKRQHPGKRALGGPCGPQGAYGQAGLLLGLLDDLSRSQGAEEKRQHPGRRAAWVREPLEE

>Ensembl_ENSGALT00000013835_Ggal

MPSIQLPVLLLCLTLSGVCLNGRQFPPELSENMGRSSLDDILQRSGSHMLQSVLKKVEKKEEMNKELNMPLPQWLSKRQHPGKRYISDPEKRQHPGKRDVEEKASFGDIQKRQHLGKTEVEGYLVNYLELKKRQHPGRRSLWDQSTDISSSQLTYLNELSKRQHPGRRYLMYKHQHPSKRGWNDELDLSDQNWEKHQQFGNRDRDSDSPDYTGPCDLQQSAICNKDSLLLDLAEKFSKEGVEEKHQHPGRRSAWENETEE

>Ensembl_ENSDART00000018054_Drer

MRAVCVFVLACVAVSGAPGVRGQDGPAEEELFQRAEDLLLRSILTQMEEQNSENDQPEWMEKRQHPGKRQHPGKREEDLEPEVEMERWRRQHPGKRAPLDLGMLEDPTALSELSKRQHPGKRYLMLLHKRQHPGRRELQEADGHSAELEKRQHPGKRRCEGWADAGCGLLELLDTSGAPEKRQHPGRRAELEDELPGLE

>jgi_102993_Bflo

MAAHLCILLLLTAIRLAHGAEPAHGAEPAHGAEPAHGAEPAHGAEPGPLAHGAEPAHGAEPGPLAHGAEPAHGAEPGPLAPWPDTEAPRERDSGTTREPWERALLEDMARVLGALALYDADMEVMNQEETSPPFTHGGAELGQLLTDSFLLQSTDKRQSPGKRQSPGKRQSPGKRQSPGKRQSPGKRQSPGKRGDDEITLPMGLRFEPLFSSALSESSTDKRQSAGKRQSPGKRQSPGKRQSPGKRQSPGKRTFPFPGTPQMETHDATDARANLLTLLDLVEQKRQSPGKRQHPGKRADWLRPSPEEPWLLYPPEKPDVLLPFISSWSPESGIEDGGMASDGARDDKRQSPGKRQSPGKRQSPGKRQSPGKRDDAETGLPQGLPFDPLFYSAFGESSTDKRQSPGKRQSPGKRQSPGKRQSPGKRQSPGKRQSPGKRGGELSAAAAGQGAYAPAGSRKDWTEKPAIYTTFPAGF

>gi109402869_Spur

MKGTVGLYMWACILGYVTWGGAALPTILGKELVLSENDGPEIADWVQGKEIPLRNQYWGDVAEEEEEDELGMLSPDSEKRQYPGGKRQYPGGKRQYPGGKRQYPGGKRQFPAGKRQFVGGELIPSPELRQWPGGXRQWPGGXRQWPGGKRQYPGGKRQYPGGKRQWPEIKRQYPGGKRSEDDQDLLAMEIRQYPGG

>KISS1_Hsap

MNSLVSWQLLLFLCATHFGEPLEKVASVGNSRPTGQQLESLGLLAPGEQSLPCTERKPAATARLSRRGTSLSPPPESSGSPQQPGLSAPHSRQIPAPQGAVLVQREKDLPNYNWNSFGLRFGKREAAPGNHGRSAGRGGAGAGQ

>Kiss2_ZEBRAFISH_AB245404

MMLLTVILMLSVARVHTNPSGHFQYYLEDETPEETSLRVLRGTDTRPTDGSPPSKLSALFSMGAGPQKNTWWWSPESPYTKRRQNVAYYNLNSFGLRYGKREQDMLTRLKQKSPVK

>KISS_Drer_AB439561

MNTRALILFMSAMVSQSTAMRAILTDMDTPEPMPDPKPRFLSMERRQFEEPSASDDASLCFFIQEKDETSQISCKHRLARSKFNYNPFGLRFGKRNEATTSDSDRLKHKHLLPMMLYLRKQLETS

>Ensembl_Scaffold GL477315_Pmar

MTPACSLAALLAVCVFGGGAVAARTDRYGASPDSNHARRARSSEEIVTGDLRASPLRLFGAVCRHAAETPRLLRLRALRGGHDLDAGLTDGEALPRSAEQDVTEFNYNPFGLRFGRRSGAQSSTAATRSRAEAACAPGKRGCRLVISKFKLRF

>jgi_125076_Bflo

MRTTAFLLASLLVLLHTILPSTVDSLALGGREAQHRQDCGGRRIRSRVPVGLKRAWPDRRGGLNSDDIFWEDTLPNSIERRQPSTEIDDEEVVDSADPDRVQYNPNAWSRFGRSMCP

>Gala_KISS2_Bflo_87934

MSPHIPGKTMVALLLILAAVASESRFAHKLPSFPGLQQTSGPYGPREPATTPLRSFTQESRWQPWKPAGVVQTSEGSSAGFGLLHEWFRIGSKRSGGAWVADTNMDDISPNMFSLHGKRNVS

>jgi_80713_Bflo

MAVEAQMTSRAVVVAVLLVSMATSHPAAHAHLVSWAVPAKIEAQPAPQYEDVADSNSLALEAHEPLLEALSPESQPRHPASQPDSYRPLLIRLPWPLARALSKRARKPPNMNAWGQPWGKRDLSLVN

jgi_96628_Bflo

MMKRRLLVAVLLLVACSMGCRGTQRDILLPHGAREDTAKSGDGRAVHDAVLLREVLTHGPTIEALVRSTMTTVLVATGWMYKMMHGKVPGLDGSSRRGLQQGRRQGEEASTLHGILTSNIATTEQNSTSTARQTTETEVSTSHTARSGWEYENEPPEYNPNSWSVFGRMVSPQASPAPPEQDDEPGVPGMPVLAEMPPKAAANLNMWSSFGRRSAADRIPERTRDGSPVRSSSVNPAFFLTPFGDQTDKQRSIEERQVKQTGDAAASHKRTPDPRVYPPNARPTLQPDWTKIPFFG

>Uniprot_QRFP_Hsap

MVRPYPLIYFLFLPLGACFPLLDRREPTDAMGGLGAGERWADLAMGPRPHSVWGSSRWLRASQPQALLVIARGLQTSGREHAGCRFRFGRQDEGSEATGFLPAAGEKTSGPLGNLAEELNGYSRKKGGFSFRFGRR

>Ensembl_FUGU4:scaffold_69_Trub

MTTAFHLGASHLLFFTLLCPIPRSIMPSPHFPSILLPFEDLSELQQHQQDHCLLQQVTRAGSGDQPHVTLLQPKQSRLRRAQRDALATRILTPLTGGPSVSFRERRQGENGGKKNEALTSIAGELQAVSREKGGFGFRFGRKRWTDWRRKSLRQN

>jgi_84798_Bflo

MSRILAVLLAVLATTAATANLPSPHRLVRRSAPSEDEVRMQRRANLLALLEDDDNETREPSEAQGAGLSKEEAEALVKYLQEPVGGLRRPAINVYRPDVEEVEREKKTTRLLSWAAEMLRMMSTKGGFQFRFGKREAAEGKER

>jgi_84799_Bflo

MSRMLAVLLAVLVTTAATTNPSPHRLARRSAPSEDEVRLQRRANLMALLEDDDNGASEATDTPVPGLSLTEEEADALVKCLVDPVGGLRGPLHTGGGFHRPRSGRGERGDREKKALDLSSLAQSLRTMGSRKAGIILRFGKRDEDEGENTSLAANI

>jgi_107075_Bflo

MGVRVMRSRICVIGLLVLMLTQSEAYSFREKSWRTSPYYRQYGGYFRRRDGGDQAPSFTSTGNGEDVSNGLDDDAGIYLSDQAGDDGISPADKRSAMLQQLAQQLKNRPREKGGFTFRFGKRESRRSFGSD

>Uniprot_PTHY_Hsap

MIPAKDMAKVMIVMLAICFLTKSDGKSVKKRSVSEIQLMHNLGKHLNSMERVEWLRKKLQDVHNFVALGAPLAPRDAGSQRPRKKEDNVLVESHEKSLGEADKADVNVLTKAKSQ

>Uniprot_PTHLH_Hsap

MQRRLVQQWSVAVFLLSYAVPSCGRSVEGLSRRLKRAVSEHQLLHDKGKSIQDLRRRFFLHHLIAEIHTAEIRATSEVSPNSKPSPNTKNHPVRFGSDDEGRYLTQETNKVETYKEQPLKTPGKKKKGKPGKRKEQEKKKRRTRSAWLDSGVTGSGLEGDHLSDTSTTSLELDSR

>Ensembl_ENSDART00000049154_Drer

MRMLCCRRVLQQWVFALFLLCSPVPHHGRPVDALSSRMKRSVTHAQLMHDKGRTLQDFKRRMWLQELLHEVHTAEIREAQQPRGGVSISSGAGGGVGAGVSITLPAGVGVSTGAGTAHPKPAGGTKNLPIGFGLEDEEGTNLPQETHKSQNYKDNMMKGIRRKKKSRTGKRREGEKRKRRARSLQEFSSGVHLQPYSC

>Ensembl_ENSDART00000125151_Drer

MLRHWGFAVFLLTIPIPIQSRATNAPSSRQRRSVGHAQMMHDRSRSLHDRKRRMWLQDLLEQVHTAQVWDSPNQSEGSVQTLSWPRPKHTGSTKNIPLGYQMESIGTRDDLPQETSKSLTYEEQPLKAAMKRKSKMCLGRWRERDRRRDRACAFQMQMHMRE

>gi_190336776_Drer

MLLIRCLEKAMLIIVLWSLCSFLFLEGLPISKRSISEVQLMHNVREHKEMLDRQDWLQLKLNNIIIPSVNDSQKERKGKTNGPSVKRLRKGEGATWSFY

>gi_47086134_Drer

MVSINGNLLKRVIFVTVFILLFSTNAESRPLRSKRAVNEVQLMHNLGVHKHVELRQDWLQMKLRGIHTASVKNTDIIPELKELYPGEAEEIMAVLEKLMNPS

>Uniprot_PTH2_TIP39_HUMAN

METRQVSRSPRVRLLLLLLLLLVVPWGVRTASGVALPPVGVLSLRPPGRAWADPATPRPRRSLALADDAAFRERARLLAALERRHWLNSYMHKLLVLDAP

>Ensembl_ENSDART00000033691_Drer

MALSLPPRPALLFLVLMSVTLMASAFPQPQLRPLQSNLPAIGQEDSKGEQWEVVYPSISLRDWSIQMLTAPDFGAAKTGREQLVADDWLPLSQSQMEEELVKGWTGDWPSRVGHQQKRNIVVADDAAFREKSKLLTAMERQKWLNSYMQKLLVVNSK

>Ghost_ORF_0_KH.C4.592.v1.A.ND1-1_Cint

MKITETMIAFVVIFCVTSSCFGKSISKVSSNDLNEKANGHSIVKRQSNADLNLQNKKTRHMNELIRQYYISSILNEIKGKRDQRQPLTADEKATACRHAVQYCRVGQVEEDNGATYLVDQKRDIENGYPDMDNTEQNNSVPNEPCLSVSAIAKLLTNSRGNSANQRTIDTSQNGHTDGETFDMYTNGQKDLASHLFQFFQDQYDLTQ

>jgi_130114_Bflo

MPRLHHLYLAANVLLVAMVILTKVTSSAGLPTANQKPLTRRSITDVTMTEERTRDMISRARQAWLTALVDEILLPEGANSKRSAIDTMADHRLAHDTQTAWRRHLLRYLVTADPSISFPQRPSKRQITDRTMTEERSRDLVDRARQAWISSLVDEIVEASGTSKRQITDVDVTSDHSKAFTDRERTAWLHRLLNAVQGQ

>jgi_69477_Bflo

MAAYTTCSLAGAVAAAALALVLVAGQTAGASALRLQQQAGRENNGSPNFDVLYQPRDPATRDDLRRLYGVLRQILSPAQHGGPFASGSKVRRTGLTDVTLLNERHGDLNERARVAWLRFLLGGIDPDASEALASEEED

>jgi_129571_Bflo

MTPLMVMMTMFFTGVLATTPAHYSNQDMKDFAVVKFHTARGRDNTTSLIRRLFDAAADMNGEGNAEKRQAERLIGAHLGETMDSSDRREHLSKLLADITGESISANCWQTSRVWRNARQSGSSELISEKPWTPPRGESTSADCWQISRVWRNAKRSDSSEHISEKPWTPEQERDLSKLLADITGMEKRQAERLVGAHLGETMDSSTGESISANCWQTSRVWRNARRRGSSEHISEKPWTPPTGESISANCWQTSRVWKNGKQSDSSELISEKPWTPGRRESLSELLADITGMEKRQAERLVGAHLEETMDSADRREHLGQLLSALTGGMRKRQAEVVVGSAQGGTLESLQRERFLSELLSTIEGEKGKRQAERLVGAHLGETMDSSGRRDHLSQLLSHITGGIGKRQAELLVGAELGDAVDASERRERLDALLSSITGHLGKRQAGMVEGTGRGDAMGTGERRGRLSGLMSSITDGVGKRQAEVLVGAELGDAVDAEERRERLSALLSSITGGVGKRLAEVTVGAHRGTAADDKKHRQMINALVAKITG

>Uniprot_GIP_Hsap

MVATKTFALLLLSLFLAVGLGEKKEGHFSALPSLPVGSHAKVSSPQPRGPRYAEGTFISDYSIAMDKIHQQDFVNWLLAQKGKKNDWKHNITQREARALELASQANRKEEEAVEPQSSPAKNPSDEDLLRDLLIQELLACLLDQTNLCRLRSR

>Uniprot_PACAP_Hsap

MTMCSGARLALLVYGIIMHSSVYSSPAAAGLRFPGIRPEEEAYGEDGNPLPDFDGSEPPGAGSPASAPRAAAAWYRPAGRRDVAHGILNEAYRKVLDQLSAGKHLQSLVARGVGGSLGGGAGDDAEPLSKRHSDGIFTDSYSRYRKQMAVKKYLAAVLGKRYKQRVKNKGRRIAYL

>Uniprot_VIP_Hsap

MDTRNKAQLLVLLTLLSVLFSQTSAWPLYRAPSALRLGDRIPFEGANEPDQVSLKEDIDMLQNALAENDTPYYDVSRNARHADGVFTSDFSKLLGQLSAKKYLESLMGKRVSSNISEDPVPVKRHSDAVFTDNYTRLRKQMAVKKYLNSILNGKRSSEGESPDFPEELEK

>Uniprot_GHRH_Hsap

MPLWVFFFVILTLSNSSHCSPPPPLTLRMRRYADAIFTNSYRKVLGQLSARKLLQDIMSRQQGESNQERGARARLGRQVDSMWAEQKQMELESILVALLQKHSRNSQG

>Uniprot_Sec_Hsap

MAPRPLLLLLLLLGGSAARPAPPRARRHSDGTFTSELSRLREGARLQRLLQGLVGKRSEQDAENSMAWTRLSAGLLCPSGSNMPILQAWMPLDGTWSPWLPPGPMVSEPAGAAAEGTLRPR

>Uniprot_Gluc_Hsap

MKSIYFVAGLFVMLVQGSWQRSLQDTEEKSRYLRSFSASQADPLSDPDQMNEDKRHSQGTFTSDYSKYLDSRRAQDFVQWLMNTKRNRNNIAKRHDEFERHAEGTFTSDVSSYLEGQAAKEFIAWLVKGRGRRDFPEEVAIVEELGRRHADGSFSDEMNTILDNLAARDFINWLIQTKITDRK

>Ghost_KH.L51.8.v1.A.SL1-1_Cint

MNLMTSSALVVCAFVCMMSQLVGGATLTSSEILKDLSSASSGEHVTQKRYAESAFTSMAAKQMDHAAFQQLLRLFGKRAIKRSGGMPETMNANPQPTEGDINDNYVVILAREDFLQKVLNNEAQNDLS

>jgi_95424_Bflo

MRCGLSWLLFFLTYTLVLLAVTALPTGDSTLARDRRALGDQGFTSDLASKLSEAEARRMIQTLMAQAIGKRFSPAEQQEPLEASKRQLGDQGVTSALAARLEQAEARQYIKDLLAQAVGKRSGGVAKRSTAEGAASRKRRALTDQGPMDQPPVGQEKSEARKILMTLLFGFGKTRAMTEEDMYSQSQQKWRRDPVAEFLDRLYKQEDF

>jgi_96553_Bflo

MGFTLKVRTFGPVDPHNKVVESRTGGGLHSLLTFSVLPYRRQVRQQVKRTTMRCGLSWLLFFLTYTLVLLAVTALPTGDSTLARVREKRQLGDQSITSEMSVRLREAEARRLLQSLMAKQGKRDRRALGDQGFTSDLASKLSEAEARRMIQTLMAQAIGKRFSPAEQQEPLEASKRQLGDQGVTSALAARLEQAEARQYIKDLLAQAVGKRSGGVAKRFAAEGAASRKRRALTDQGPMDHPPVGQEASEARKILMTLLFGFGKTRAMTEEDMYSQSQQKWRRDPVAEFLDRLYEQEDF

>gi_161281871_Ctel

AVLLVATFVAMVTAAVLPSTEGDIEDLMDAQDDIDKRAFSAWGGKRGFHAWGGKRSEDNEMDKRAFSAWGGKRAFNPWGGKRSFEDQEAERGNFEEQKRQFGPWGGKRASPEIYKKAFNPWGGKRSSTSSLEDFLSKRSFNPWGGKRSSGWRAQE

>gi_163494026_Lymnokinin_Lgig

RFNSLKPFKGSHVISKRAAFAAWGGKRAPFSVWNGKRAAFTSWGGKRSNDEYDLDKKAFRYWNGKPSDLNDGGDGNVDMDKKAAFTSWGGKRSDFGDEMDKKSAFTSWGGKRSDYGDEVDKKAAFTSWGGKRSLYDLGLYDLNKRPAFSSWNGKRSLFPFHLSDNIPDPEFNENFRLLTKRKPEWNFAKNYLRAFVTRLLNERAPKRSFSAWKGKRNGELVYNVE

>gi_121439720_Acal

YCLSDLSTGVPDYAVLCEVQDSAVRDLTTSVHTFTKRSSGPLDSLEILHFGNGGKFGEFSVKRSDNVDHENEIVVVNTEVPEFSEGVVSKSDVLEKLADDFLEGEGTNIIPENERDEVDEFPDDSGTEDLDKRAGFAPWAGKRDGDGVVDKRPAFHAWSGKRTPHSAVDAQKRPAFHAWSGKRSTYPYKKK

>gi_117947799_Ipar

MMGNRRLLIIMLQVFAGLLLTCAGQTNCNGNEGCNRESFDDQSLIDTEPIEDKRKFSPWAGKRAGESNLASYIIRTRTNSKPPVITHKKSRFSPWHGKRSGAELSDAELSDNILERLLLQYSDSDIGRLQKMLAKRGFNPWAGK

>gi_83930519_Llon

MLFLRMFPLAFLTVLCGLCVSAEYHGDVGVGISPWTYSNIQRIQSLEERINQYRRFIIHLFGKLPGSCDLLRIFEEYSTPQFPDHSPDIQHLIEKLQPPTAENHAETRLPTDPKKNLLDILDIYLDFTAYVMDPVSTEGIERSLRRGCETDLLTNPADKRSDEKVVGKKIKFHSWGGKRHQVNLDSGAYRIITRTPFHSWGGKRAAMDSPYDFY

>FBtr0075722_Leucokinin_Dmel

MAKIVLCMVLLAFGRQVYGASLVPAPISEQDPELATCELQLSKYRRFILHAILSFEDVCDAYSSRPGGQDSDSEGWPFRHYAPPPTSQRGEIWAFFRLLMAQFGDKEFSPIIRDAVIERCRIKSQLQRDEKRNSVVLGKKQRFHSWGGKRSPEPPILPDY

>Ensembl_C45G9.13_Cele

MSLAQSTFYLLFVAFLAVVIAVTADKQQSAYDLETPISAYKRFYSWEDAKRAASSEEGMRNKRKQFYAWAGKRSSAPVHYFEDSIAQEAGPSMEKRKQFYAWAGK

>gi_54551733_Xind

ARGGSREMEKSEMQNDLVKRKFYAWAGKRSDYKRRFYAWAGKRAGGGAAAEGHESPENDEAIEKRKFYAWAGKRAADEPDEHESPEKRKFYAWAGKRSEDLTEPEKRKFYQWAGKRSEDEEHMLDGPDKRKFYAWAGKRANDGEEQEPRENPDKRKFYAWSGRR

>gi_21817974_Tspi

MMSSVKSCICIFTAFCLLASFTSATLVDSPSSAKEVQMIPVFYSDTVGDHHVIDPTVDNLEDVSELNNLMKRKFYAWAGKRASRFYQWAGKRNGEKVEKVRRKFYPWAG

>jgi_scaffold_397:50652-51471_Ctel

MVNSIPALVVAITLLKLSSAIFYTNSKENDYPRLGRRASISANELWDVRRESDLGSQKSNFEEILDPRDQELGVVIANMMNQHKGWIYPTPCFMLRFVSEQFSKLADIFFFPISLLLICRYVF

>gi_223784846_Apom

MAGDQLVARWSQLLLLLFLVAKALAIFYAQNEGNDFPRIGRRSSLLAPRSLKESDGGLDVHSSGRQDGADFTPLRDYLRRRELALMVASYRQTEFPFAALRHEIRRTKPTSGQ

>jgi_173607_Pleurin_Lotgi1

MRQEVLLLMFCMFTTSCCGVFYTSSKANDYPRIGRRSFFTSSKENTYPRMGRSGDDGKVTIDMLKRRFTAGDSASYPRLGRAGYFTKGAASYPRLGRSSNSLDVEDIQDLMPDSDEELPQRNIAHLGLQAPLAFLLWDKNGDGHLSKSEFLTGLSKTRHRPSHKK

>gi_270524441_Hdiv

MKESLVFLVFAVAGSCAGIFWTNSKDSDYPRIGRRSFYTASSENTYPRIGRSGGVEDTFRDGIETRGSFFTQSDENTFPRIGRGNDNSDGDNVNKVRTKLQI

>gi_168891936_Daphnia_pulex

MIKGLFLTWLLLASALSDPSPEPFNPNYNRFRQKIPRIGRRGEGIIAEYMNSESFPHEGSLSNFFLKASKAVPRLGRRKDISTESGRAAMVGEEPFGRISNEIPIMNQKQDLWPNMNINELTGALNKELNYPGPRIPKDLQDNYIQDLIHSWINQYENLNEN

>gi_288558759_Tribolium_castaneum

MRRYQILLIVIFFNILFDSTLSENYVLKAAKNVPRIGRSNTNKNTNIDEMGKFFMKASKSVPRIGRRNENFDYGQPIVKRDEGGFVISSLLLHYCSILVPIWSDIADRFEYDPEILTSPEILEQLEMGDDPSVYEWEKIRTKRDSHKPHPKFYYVM

>gi_158218475_Acyrthosiphon_pisum

MSGYLAIIVLVCLQILRVMSINEFPEKKVQNIWLADLDDKQIASRIERSDQFETASDVLMKDASVYPKITRRGFAGEEFFLKASKSVPRIGRRNNDIQETPKRSLSKDQVNMVEYWPYLQPNDINDLTRKHDFDLPYNCQQLDAKTIFLVDMYNFVCNDQFYCCAPAKRAIANSPNSNSFVKKRSTTSYRNIIFL

>gi_158183626_Nvit

MKRLVNTFISRNYILSAIFIVAVLAIIENKIVAADEPPAFFLKIAKNIPRIGRSEPYDEYAIKNSNVKDDIPWHKGEISKRRVGFSPESNTYAWQHFPLAIEGPPELWRTLAGYSHDPLYKTTDDFNNELWSRDKRTNNPEA

>gi_210142303_Amel

MTSLRSLGFVRCFFVGAIWISILALLICENASMADEVPAFFLKIAKNIPRVGRSEGYDDFFKSRKNLPKLGNHDGQSESWPQYSVDEPFSRPIKRRVDYPSVDDWSWQHFPLAIEGPRELWRTLAGYSKDTSDDVDNEIWKRKKRTGNEPMASEEN

>gi_288558757_B.mori

MTSKLTMMLFTLSLMFIAGLDGSFIKPNNVPRVGRSNEAFDEDVMGYVIKSNKNIPRMGRRNYDSGNHFDIPKVYSLPFEFYGDNEKSLNNDDAEEYYAKKMGSMKK

>gi_55877638_Lmig

MLLCKETLASLAVVLVVAAAAAAPEEGGGLLLKPHVARRSDFFLKTAKSVPRIGRRSDLFLKSAKSVPRIGRRTNLAPIEAQDGGEWLWPGGADALPMPARRQAYYVRKDGQPVMWSDVARDVEENPDLWPWSDFDSG

>Ensembl_FBtr0072426_ETH_Dmel

MRIITVLSVSLLVGLVAISQADDSSPGFFLKITKNVPRLGKRGENFAIKNLKTIPRIGRSEHSSVTPLLAWLWDLETSPSKRRLPAGESPAKEQELNVVQPVNSNTLLELLDNNAIPSEQVKFVHWKDFDRALQADADLYSKVIQLGRRPDQHLKQTLSFGSFVPIFGDEQNPDFMMYKNNEDQELYGGGNRYDRQFLKYNIL

>gi_163510328_Lgig

MKLSEMIMCIMAVLLVTITLGNGAPQWRPQGRFGKRVNPTLSLLLHGDQEKQFLNHEKPFISEDKPYIPEVTDEISVERLTSDARDNLSKFLHRLCSESGLENVPRCSWSRKTSERSSDPIRLI

>gi_144925775_Lstag

MISTCCVLSVIFSLCYATPHWRPQGRFGKRTTEDDNVPDFGISRPQDIPIELMFSKNELSRRNSKPRLCSTSGVAGYPPCDAQVNAMQVATLGEPQDLANLFDL

>jgi_213930_Ctel

MSKLIIMGILSAILALLSITPSSAEGQFAWRPQGRFGKRMDSTPVNSLDNAGVARNPGFWSIVCNCIYNQKRKQKMVEMSSMLHAWAISTLRMISNINLAFGDLVVHNAWVKPAFFFDPDWWNAIYGGTMD

>gi_223786475_Apom

MATFRAIVMGLASLILSLLILTQPSEQQYAWRPQGRFGKRDNYLARGILSGPSLDTSNKIKMLSTHMFESNGVLCVSAGSRGLYRCFTVNPVPGSSSPVSMANTPFIEVGDVLCVSVAEIGAYRCFQLDDHEVPEQSLTNH

>gi_194267062_Iobs

MMELMLVACVLVVMTSLLQPTAATPSWKPQGRFGKRGGPSDYLGIQKKPHGWRPQGRFGKRSLASSSSPSSSSFEQSIDAELLKSAAVSGAIEVPVELLSTGENGMFLRLGVKPCSITGMDGIPPCTGASEGASETF

>jgi_251691_Dpul

MARKESVFWLFCTLALMMSVVLVDAQTFFTNGRYGKRSEVRSRVASRSADERFFGGPRFGRSGNGGIVLGNSELDARNPERFFIGSRYGKRSEMEQIVPSPQVDESTSNSQEKETFLECNPIGIEQLYHCIERLKSAHHFDLMQHQQV

>BCM_GLEAN_08650_Tcas

MHARKLIVVLVYILTVLVSVAVSKRYTSEKRVQNLATFKTMMRYGRGGPSPNNKENKVNIRPRADAFFLGPRYGKRSGWSPNASLVYPVSTPLCGLDEDLSCAYTGISDLYRCTPRKGESEEFTTSSN

>gi_268286147_Dpon

MLVRKVPVIFVFLVSIFITVVNADMESIKRIPSHSFKQMMRYGRSSANNEKINVNPRADLFYLGPRYGKRAGPTLAKLDTVYDSTFMPCTYTGVADLYRCDTSRSTMREDRRK

>gi_18866372_Agam

MTRRTVQPLAREQSSSSSSSFLFSCISTTVALVLFGALVLAAVADADPMVDSASSLYGDNKHASDKRPFFVGSRYGRSHVYGAKDMRQVNVVPRNDRFFLGSRYGKRSDLTKEIESDNNNGIAELTYLACLHTGVSNLYRCYSRDSSSNALHQQETEQFDQ

>gi_156462907_Isca

MLHCRAWAVALIALLVLSVASAAKGTPQFVPNGRYGRRSVTPPLAGVSKDITVNFFGDSTISCTHTGFADIYRCTRKTSDDYKDSSFE

>Ensembl_FBtr0310523_NepY_Dmel

MNECVNKLLHLKFLFYFILGIQKRPVFFVASRYGRSTTYDESLKSRRIFIVPRNEHFFLGSRYGKRSGKYLCLSREINKLIVRKRLRNNDKERTPTLSFITKHFLMRNT

>gi_BQ542910_Tspi

MDLKIKIRIIFFLIGMALFSEAKPASLARYGRAALPRYGKRAELISGLDAMRDEVTEFKNSPCVYTGYEDLYRCSSMT

>Ensembl_Y75B8A.11_Cele

MLTRVPVLILAVIVMLALCQEPEKPEKRPALLSRYGRAVLPRYGKRSGNLMESSQNSLTEESSDVVCQLIDGKYICLPVDAGFLGEVNGSIKQNSKTKCPAFFKNVSFSLFFRAFYLTVPACTSKKNVLLTSTTSTINHQKLEPATTLSPIRQRRRCLFQIDFAYLTVCPSKCQEGTDTGKVADYLLSLGCSDMDVIEREMKRVCCPE

>AltB_DB915757_Idiosepius_paradoxus

SESKSLKPLSVIKAENLKSWAKRSTTNGKVLNEIRQAVARGVFPAAVGPIDKSGDKYMSFLQHWLKNNGYPATMANLWARNTGFKSRVTREINNNDDDLPDKKDRLDTWNSMNTWGKRSPNTWDSMAAWGKRNGDTWDSMSAWGKRNGDTWDSMSAWGKRNGDTWDSMSAWGKRNGDTWDSMSAWGKRNGDTWDSMSAWGKRNGDTWDSMSAWGKRNADNAGDKRDWDSLQAWGKRNIAP

>jgi_197880_Ctel

MASCRLLLTVITLVICSLVVLADDPETEEQAQDLVPHDMDQDLMDKRKWGSNSMRVWGKRDGDDEMEMDGGAEKRKWGGNNNMRVWGKRKWGANSMRVWGKRSELPEEEKRKWGGSNTMRTWGKRADDNEEDELAKRKWGSNSMRVWGKRADDNKRKWGSNSMRVWGKRADDDMDESKRGWKNNNMRVWGKRADDEIDEDKRKWGSNSMRVWGKRSADDDAELAAAVPHAIVKRSLDSEEFTDDMEKRRWGGNDMRVWGKRRSRADGPKRSWKTNVMRVWGKRGWADNNMRVWGKRADEGAEKRAWVGDKSLSWGKRSDNEVIRNLLAEQVMMISIISPTKYLRDVAICVGGVLGGFSRDPLSE

>jgi_231501_Lgig

MDLKTILCLIIYSLLLQISHAEEQLANDIELSNSLNPVDKRAWKSSYLNTWGKRWNPRYNLRGYQRMPIWAKRWTNSGLITWGKRSADTEIPIHKRKWNQFITWGKRSGVPSIVKRSVGDELVPWGKNKDTLPELNTSSDNLDNKLIDLETTPSTDKLSLDEKRASDKGWNGFTTWGKRANKDWSSLSTWGKRGQNKDWSSLTTWGKRGHDRDWNSLTTWGKRANKDWSSLSTWGKRARENDWSALSTWGKRANNKDWASLTTWGKRANDRDWNSLTTWGKRAKGNNWSGLTTWGKRANKDWNSLTTWGKRANKDWSGLTTWGKRGNKDWSGLTTWGKRGNKDWSGLTTWGKRGNKDWSGLTTWGKRGNKDWSGLTTWGKRGKNDWSGLTTWGKRGNKDWSGLTTWGKRGNNDWSGLTTWGKRGKNDWSGLTTWGKRSPDATSEDSGELSTLDKKDIKGWNGLTTWGKRFAGDKNKWSSLTTWGKRDDNNKQDDKKWAQLSTWGKRSPEDAAELWKIYDSNGDGIMDKEEMVSFLRSAASQKDSQQDEKS

>gi_300048851_Apis

MQNVLGRIAATLVILCPVIVFTIPESAIQASSIKSSQTEQDNSDYTRSFDEDQEEKRAWRDLQTAGWGKRGWQNLKTTWGKRAQDWQNLHSSWGKRQGWQKLHGGWGKRGWKDMQSGGWGKRFKDQPASSQLSQFDEYLDKYEEENPNEAEKRSWDNFQGSWGKRAADWTSFRGSWGKRNPVDYMNEYSXYGDNDNYKAYIFPPGYNNYLPNFQTEYEK

>gi_298366123_Hvir

MRYCVAALWLSALTTLVVAAADDAHHDVAPPQTDNEIELSEEEKRAWSSLHGGGGWGKRGWQDMSSAWGKRGWNDMSSAWGKRGWNDMSSAWGKRAWQDLNSAWGKRAWQDLNSAWGKRAWRDMSQSPWGKRGWQDMSSAWGKRGWNDMSSAWGKRGWNDMSSAWGKRGWNDMSSAWGKRGPEKWANFHGSWGKRSEEPDYEEIDAAIEQLIPIQQLSDSEHMEAPEKR

>gi_219522029_Tcas

MMSFAAAIMRDAVAPVLGAVLLTCYSLQATLALSDETPLKSSNDNPQIEDEMSKRDWNKDLHIWGKRGWNNLHEGWGRKRSVPAWEEQQEKRAWQSLQSGWGKRFAPEDEYAIRQLAAMLDSQYDDYNPEIETNDDEKRNWGQFHGGWGKRSKWDNFRGSWGKREPAWSNLKGIWGKRSGEK

>Ensembl_C01A2.7.2_Cele

MQLIHFIVGLAMLISLSLAASDDRVLGWNKAHGLWGKRSVQEASQDKRTPQNWNKLNSLWGKRSASSFDDDYTTENGDDDVTMLYKRSPAQWQRANGLWGR

>MPI_GENEPREDICTION_JIGTRA00000190063_Ppac

MQSTIVAAVFAALLLVSAAQEEPRGWNKAYGLWGKRSLPMDEDIMALEESAMPKRASERSWGKLNNIRLIEQQGDFLVRASSPGTTTEIILTACDNRSMPVHAALRFDIVWFFPAKEGGKQLSFPTVSDLIDHYIDHPPKNVPLTRAIPRLEPELRLDNIYYEGNHCIGRGAYTTTFWGSLTRPFHGDNKSIAVKIPTVAEGDKAVNMAARRAQLLEESAKLLYCLHPNVARFLGIAFDATYLLIVSEHVPGKSLHRHLTKFGVLTDNREKLLYLLEVAAGVNYLHDKGIVHRAIAARNCYVTVTGKIVVGDYGVNTLIDTGFGERHSCWCRQGPPQRWLAPETLGSSTAFSKASDCWALGILAYEIFANSEQPWPGMTDLQMECEIRKGTGPAMPAATPDRVVVLLKRIWDATPQRRIAIKQIGKEIINMIEDTIRLIGEFPADLLTLNRIPQVTRAVYYEPTAYDFTKVGDVLTASQWTSNSSSSSSAETSPVFGSTLINVKEGSTTRSSSATSLAPPAPVSPAPVSTYFDSNLKENR

>Ensembl_FBtr0079562_Dmel

MGVPRSHGTGIGCGSGHRWLLVWMTVLLLVVPPHLVDGRYLPTRSHGDDLDKLRELMLQILELSNEDPQQQQQQQQQQQHPQLRLHNEATGGSSSSSNINNPRVSNGNSNAAWLQKLSAMGALDELGGDGARFGPNYGRY

>BCM_GLEAN_15137_Tcas

MFDRKLVFALVFVVFATLAVEGRYLPTRSNGDRIEKLRELLKDLFENEVEKEEYQADAPPRWHPESKLFYKREAPAH

>gi_300039604_Apis

MAGKFSALFLVGFVAAVVVAPYMMAEARYLPTRGNDDRLTRLKELLTDLLDSGAQPNLEMERPYVDVNGDFSRLRPREYNIPEKSIMELFNPTVPHHQRPRS

>jgi_305680_Dpul

MLKSTSLKALVTLLVVSFVLMASSPRAADARYLMTRGKDPRFDRLYDIITKLLQNGGGDLEYQIKSQLDSGP

>jgi_21488_Ctel

MEIRLTLVLALLVAALGVVANALSIPSDALKDNQMDVADKDGDQSRRATWLETRDLEDDFKELVYLTIEELVNEGRMDPRVLSKEENEVKEKRGRWQGFCFKRTRSGRFLPYICWKGDRK

>gi_223843442_Apom

MDMCRLSAILVILYGTLCAVTNAMSIPASQLKDSGVELDSSENTALDEGRRASWLETRDLENDFKDLVFLTLQELAQEGRIDPRVIVEENSLDTKEKRGRWQGFCFRRTKTGRFLPYICWKGDRK

>gi_163376585_Lgig

MRSSTILSLLVVLLLAAPTFCLPPDQGSEIDDLDKRPKYMDTREELSVLKDMVYIVLQELAEDGKINPELFTIHDQKAVVKRMKYMGICMRRTKYNAVVPYPCLRSGR

>gi_121437918_Acal

MELQACNIFALFVVVVTLSVASSLPASRTDDVLQEASGLALNKRPKYMDTRRDLDVFKDLVLISIQELVDENRLNPALLPEEDAPKPVEKRMRYMGICMKKQYNNFIPFPCLRSGR

>Ensembl_FBtr0085024_PDF_Dmel

MARYTYLVALVLLAICCQWGYCGAMAMPDEERYVRKEYNRDLLDWFNNVGVGQFSPGQVATLCRYPLILENSLGPSVPIRKRNSELINSLLSLPKNMNDAGK

>gi_45650472_Pmon

MCRVAMLLVVLAVTAVVVTEAQREPAASKCQAATELAIQILQAVKGAHPGVAVGPHKRNSELINSLLGLPKFMIDAGRR

>gi_283526164_Psed

MNSIIVLSLAVLAIFTSSIQSSPLNRAEDLQPVERQIIAEMASKILKVAEDGLVYSKRNAELINSLLGLPKMMNDAGK

>CO508274_Hypsibius_dujardini_Tardigrada

MDVKAFLFAVAMSTFLTSSSAIWVFLEPRFDAEFISRNSEMVGQEVYSQFPSGSEGPAQLLSQGYQPVELTRIPIRKRNSEILNTIIGLPNKLRQRG

>Ensembl_F48B9.4_Cele

MSSRISVSLLLLAVVATMFFTANVVDATPRSQGNMMRYGNSLPAYAPHVLYRFYNSRQFAPINKRNNAEVVNHILKNFGALDRLGDVGK

>Ensembl_T07E3.6a_Cele

MNRFIISMIALLAVFCAVSTASPLLYRAPQYQMYDDVQFVKRSNAELINGLIGMDLGKLSAVGKRSNAELINGLLSMNLNKLSGAGRR

>jgi_239524_Lgig

MVALSPEMNSCTKCLLLLVFVGLTVLVKLSESSPLRPYQTQIEEKSRQDIITLAARVIKIAMYGSNQYDVIKRNAGTVDHLLNFPDLSVGK

>gi_187067819_Skow

MASYKSMMVIYCVFLLVADIEGFLKVVAKKSGRIQREVAEKRDLLRDKEIEKRPHGERDLRNYGNTKQEVDIPRHMVLGR

>Uniprot_NT_Hsap

MMAGMKIQLVCMLLLAFSSWSLCSDSEEEMKALEADFLTNMHTSKISKAHVPSWKMTLLNVCSLVNNLNSPAEETGEVHEEELVARRKLPTALDGFSLEAMLTIYQLHKICHSRAFQHWELIQEDILDTGNDKNGKEEVIKRKIPYILKRQLYENKPRRPYILKRDSYYY

>Ensembl_ENSDART00000142353_Drer

MQMQLTSFLLLFLLCNGLCSDIDQGKRAIEDEVLRSLLTSKVKASRHIAPLWQLPLQDVCRMVNGLGDSWLEAWANEEAAEDTEVHADYEQRVSGTLLQMLEEMHDIQNLCRVLQPRELQDEQEYLELEQNSDSPLKRKSPYILKRQLRTNKSRRPYILKRSVIY

>Ghost_KH.C2.14.v1.A.ND1-1_Cint

MSWKCFSAVLVVIACCGGVKGQESLNEESMPQEIYKDLQNSNNLPGYESESKGVVNHVTNMPDAEILQKILYLLEEERMPSENDQRTGTAAGNVATRQLHVPSILKRFSMPSVIKKGMMGPSIIKRIGPGPSFGGQQEQKVVEPTTRNHRMMLGPGILKRFGMIPSIIKKRRFMGPSIIKRDGAQEPTDNSYNY

>Ghost_KH.C2.201.v1.A.SL21_NT2_Cint

MSSTTLMSRVGILIVVIICLQQQQLTQGIPLSEEIQELLKNRVVSNEADSSDISDYYDQGLAEGNGDSSELFLWFIYKSLLADLKKTGKNNVEVEQRNKLLYPSVIKKSRHPKLYFPGIVKRSVQSGTENKQTDETTF

>Ensembl_reftig_209_Csav

KGHAVVTKQLHVPGILRRFSMPSVIKKHGMFSPSIIKRTEPEADDEVQQPASRSGYMWIPSILKRYSMMPTIIKRNRFMSPSIIKRDTDGQVYSGVGLH

>PENK_Hsap

MARFLTLCTWLLLLGPGLLATVRAECSQDCATCSYRLVRPADINFLACVMECEGKLPSLKIWETCKELLQLSKPELPQDGTSTLRENSKPEESHLLAKRYGGFMKRYGGFMKKMDELYPMEPEEEANGSEILAKRYGGFMKKDAEEDDSLANSSDLLKELLETGDNRERSHHQDGSDNEEEVSKRYGGFMRGLKRSPQLEDEAKELQKRYGGFMRRVGRPEWWMDYQKRYGGFLKRFAEALPSDEEGESYSKEVPEMEKRYGGFMRF

>PDYN_Hsap

MAWQGLVLAACLLMFPSTTADCLSRCSLCAVKTQDGPKPINPLICSLQCQAALLPSEEWERCQSFLSFFTPSTLGLNDKEDLGSKSVGEGPYSELAKLSGSFLKELEKSKFLPSISTKENTLSKSLEEKLRGLSDGFREGAESELMRDAQLNDGAMETGTLYLAEEDPKEQVKRYGGFLRKYPKRSSEVAGEGDGDSMGHEDLYKRYGGFLRRIRPKLKWDNQKRYGGFLRRQFKVVTRSQEDPNAYSGELFDA

>Ghost_KH.C10.27.v1.A.SL11_Cint

MKLVKKFSILAAIVVCYFGCIADAVPVDTVEKQLLQREGTGNPENFLDWTNQLNSTDDAEDNPFLAENRNSDEENNDYSPGQAESIQSDKRFQSLFKRYPGFQGLFKRHNPHLPDLFKRYNSMGLFKRSPGMLGLFKRGLLGLFKRSDARLQGLFKRDSATQGSFKRSSEAQALPKRYPNFQGLFKRLSEATEYPEDDSSNDDTKQRGNLHSLFKRDTSAHYLEDRGESIPFLFRRS

>ENsembl_SNAP00000092003_Csav

MYSIQACLLLSILFNPDSIIVKAVPAIDAIERELISKQINKEEVNFIDFTKALNSTALDDMSDDELLWMLGQMIQPIQESTSVNDEEQLSGIDDIEEIDKEQGTNDELNLGKDGDQNQAYFSPQMKRHFGPLFKRMRGLFKRAPNAPLKRGLSGLFRRDPNTPGEPDARKVSDGGRTDTRGMQGLFKREPAFPVDADNSYDGKSHQVKLDRRRMPGLFKRGLQNLFKRAGGANSLFKREDGIENEVGARNRKSDLQEIFKRGIQGLFKRMTTDDLNAENNLGEAPHYRRSFHSLFKRDLSLSWNKSSGENKNKVHSLEKRGENIPFIFKRN

>Ensembl_ENSDART00000076405_Drer

MPLRCRASSMFLLLCVSLSLCLESVSGGTSFLSPTQKPQGRRPPRVGRREAADPEIPVIKEDDRFMMSAPFELSMSLSEAEYEKYGPVLQNLLENLLRDSSFEF

>gi_55792853_Asch

MFLKRNTYLLVFLFCSLTLWCKSTSAGSSFLSPSQKPQNRGKSSRVGRQVMQEPQQPTDDKHITISAPFEIGISMTEEDYDEYGVVLQEIIQRLLGGTEAAEGPPQL

>Uniprot_GHRL_Hsap

MPSPGTVCSLLLLGMLWLDLAMAGSSFLSPEHQRVQQRKESKKPPAKLQPRALAGWLRPEDGGQAEGAEDELEVRFNAPFDVGIKLSGVQYQQHSQALGKFLQDILWEEAKEAPADK

>gi_21238918_Ajap

MRQMKRTAYIILLVCVLALWMDSVQAGSSFLSPSQRPQGKDKKPPRVGRRDSDGILDLFMRPPLQDEDIRHITFNTPFEIGITMTEELFQQYGEVMQKIMQD

>gi_68445391_Ipun

MLGHGRVGHMMLLLCAFSLWAETVMCGSSFLSPTQKPQNRGDRKPPRVGRRTAAELEAPLPSEEKIMVSAPFQLAVSLSDAEYEDYGPVLQRMLLDVLGDPPTLDGAN

>Uniprot_MOTI_Hsap

MVSRKAVAALLVVHVAAMLASQTEAFVPIFTYGELQRMQEKERNKGQKKSLSVWQRSGEEGPVDPAEPIREEENEMIKLTAPLEIGMRMNSRQLEKYPATLEGLLSEMLPQHAAK

>Ensembl_ENSOANT00000031359_Oana

MVSRKAVAFLLVVSVAAMMAEGFIPIFTHSDVQRMQERERNKGQKKSLTVQQRSEQGGLRTLAEPNGEEEGEIIQLAAPVEIGLRMNSRQLAKYRGILEELIMEALLSTQNGESNPDRGRGRCS

>Ensembl_ENSOCUT00000005807_Ocun

MVSRKAVAALLLVHATAMLASQTEAFVPIFTYSELQRMQERERNRGHKKSLSVQQRSEPAAAPPAEPTLEEENGRTQLTAPVEIGMRMNSRQLEKYRAALEAERAVHPDAPSRPCPAGGESGWSGEPSPT

>Uniprot_SMS_Hsap

MLSCRLQCALAALSIVLALGCVTGAPSDPRLRQFLQKSLAAAAGKQELAKYFLAELLSEPNQTENDALEPEDLSQAAEQDEMRLELQRSANSNPAMAPRERKAGCKNFFWKTFTSC

>Uniprot_CORT_Hsap

MPLSPGLLLLLLSGATATAALPLEGGPTGRDSEHMQEAAGIRKSSLLTFLAWWFEWTSQASAGPLIGEEAREVARRQEGAPPQQSARRDRMPCRNFFWKTFSSCK

>Ensembl_ENSTRUP00000031321_Trub

MQLLVVLAALTGVLLSIRAAAVLPVEERSPVHLNRELSKERKELILKLVSGLLDGALDTNMLPMETVDLEEPLESRLEERAVYNRLSLPQRDRKAPCKNFFWKTFTSC

>Ensembl_ENSTRUP00000025247_Trub

MQRIHSSTILMLTTLVLCIQGVSSQPDRDLNQNQDLEMEVRHHRLLQHVHGAGLLSQEWSKRAVEDLIAQMSMPEGTGQRDTEVVSMATGGRMNLERSVDTTNNLSPPERKAGCKNFYWKGPTSC

>jgi_72051_Bflo

MARLLFLLGVLLVALAAIEPTTSEDLTDDSRELEDIEQDLEQTEFQVSFLKKQLLRKLVEAYAKGQDLSPQEQRQLELLPPGDKRGMIRRAKGCARFYWKMPATAMSC

>gi_161295377_Ctel

MVPSKSVCAIVVVMSCYVLCGFIVNAVPTQDGGSNLSHGYNEVSEMGLTDTKLAFLEKKLREEIERDLADLLVMEHQHRHNLNFIQEKKRQLEIKKREPVQCLVNIVSCWKRK

>jgi_181187_Hrob

MKNVTFLLLAMSATLLLLLASALPPRRRSLISKNYIFPKTHSKRFAKNNDAEDPLYSSYPFEYNNNEPISMDSNKYANSLNKEFPKAPKGKQVFKKAHVPCLFSIISCWK

>gi_163505903_Lgig

MKSLPVLTLTLFCICLNEVSSNKILPGEEHRQLVQITEESSNNDRLLKRSTLQDAYESHLAMLMEAEQSLTKQIEELKLRREELSNRKRSHVMCLVNLISCFRKR

>gi_260628238_Cgig

MELTQSVFVLKLYAAVVAVLLVAEVHAQPQKFSTEIQQTGDESSTDLNLFKMALREAYNRELEFYEQQEAQIVKQLAALENDRNQIRERKRSHIRCLVNVIACYRKK

>gi_203663510_Acal

MSVSVRTWRAVNTCLLLTLLTLWADVLVVRAAVIPVSSPEPMEEASALQLLPGKIGRASLLREMERQLMILQAAEENIVSGLQELEEERRVLSGRKRSHYSSMCMFNVVACYRKRK

>jgi_290676_Dpul

MMAKISAVVPVAILLYLAASGAAKSTDREETESTDFGQDIEVLGAVPDDGSVETALLNYLFAKQIVARLRTNANPQDLMRKRSYWKQCAFNAVSCFGK

>BCM_GLEAN_05428_Tcas

MAAQLPRYLTKTLFIFLIATLVVANARPNHFGDASQNSISGLKFQVVGEPADGNNLLDSRLKPWELEMLVQRLSEISSQTGGDFAWDKSIRLPEAKRQSRYRQCYFNPISCFRK

>Ensembl_FBtr0080234_AstC_Dmel

MMKFVQILLCYGLLLTLFFALSEARPSGAETGPDSDGLDGQDAEDVRGAYGGGYDMPAQAIYPNIPMDRLQMLFAQYRPTSYSAYLRSPTYGNVNELYRLPESKRQVRYRQCYFNPISCFRK

>Ensembl_FBtr0114455_AstCC_Dmel

MVVPKRAALLLDRLMVALHHALEQERSEQRIGEFFGDRNILSGKFGDSHNGMEHHQAREDGMYSDDDAGTLLDYDFKDLNQINRATGETRRAGADRSGTSTHSGSPAGSRRIQPSGSGGGRAYWRCYFNAVSCF

>Ensembl_F33D11.8_Cele

MVKFFVFLLFFAFLCSFTSAIPLRSLFLRSYDDINQESIARGYFAPQPVDDDNADNRPKRGIDLLKRRVEIIERNRCFFNPITCY

>Ensembl_M02E1.2_Cele

MMAQKTLIIAVMLVCSILQPMLALGSLTPSAAFRANMQQRERSPNTLFYMDGASKQYGDEIKDPIYKRFKPCYYSPIQCLIKRK

>Ensembl_R11.2_Cele

MRVLTFLLVTLFALANVMQAQRYDRAIYEALLNDLEREFVERELAQHVLEKRELLRQDRQELDRVRRASEKKSYPRNCYFSPIQCLFTRN

>Ensembl_Y73B6BL.35_Cele

MKSSSVLSVALIVLVIVQLISASLASVPSSSAVSDGQIDFDALAAKIEMLRPNRYWKRAHNIDTRALNQFKNCYFSPIQCVLMERRRK

>Ensembl_R08B4.5_Cele

MSSGKLFQFFIVFLATLLLADAIPMVSSRDEDDQIIQKRLSNDALIRLLMRNRGTQTQLGLKRGLVKKAEVERRSIDEDFSNCFLSPVQCMLPSSRK
