# Supplementary material for: Neural Ganglia Transcriptome and Peptidome Associated with Sexual Maturation in Female Pacific Abalone (Haliotis discus hannai)
Source: Genes (Basel). 2019 Apr 2;10(4):268. doi: 10.3390/genes10040268 (PMC6523705; doi:10.3390/genes10040268)
Supplement: Supplementary file 1 [file genes-10-00268-s001.zip › Supplementary File 2.docx]

**Achatin** MLPNWLFLCGVGLICLGWALAEENEFSDNLHTYNEEDYDPDEEFDKRGFGDKRGFGDKRGFGDKRGFGDKRGFGDKRG+

**Adipokinetic hormone (AKH)** MPSVRSLALVLTVVCLLSQTFGQISFSPNWGTGKRSSALGLDHDCWGKSELRILYDVVKVIRRQAESLARCMQDTEAEEAMLKH*

**Allatostatin A (Buccalin)** MLLRMTPFCLGLVTYILLASSTQAEDVVVPQTPDDASESEVKDNVDTDGYYQDVSKSVYATNNNDEKRQSVDTLGFSGTLGKRRIDPILFGGRLGKRGMDPLGFAGTLGKRGIDNLGFAGTLG+

**Allatostatin B (WWamide)**

MDLKSFFCLILPTLATLQLSLAEDPSLVEGVKPLSGAGQRPLQNSDSAADKRQWSNFHSWGKRWGNFGTSGKKWASSDFPAWGKRWSGSSFTSWGKRNASPEEQKRNWNQFITWGKRNWHALTSWGKRSQEKSEKKWNTLPTWGKRAGWDNGFASWGKRDEVDVRDQLMKLFDESGDGFLDTKELGNFLAWMSSLNDMAAHP*

**Allatostatin C** MRSSVFLVVLVAFVLLCQVVACMSESEKSSLSGHGSSSDTVLLKRSLRQAYEDQLAMLQEAEENLTQQIAALQHQRQEITQRKRSHYMCLVNLITCYRKRK*

**Allatotropin** MMRTTLLMLGLLLVAVVASLPHNTHSRQKRGFRANSADRVAHGYGKRTYSSWENSFSTDSKHLLSVEDLANLVSNNPTLAEALIRKFVDVDDDGFVGNNELFERSNE*

**APGWamide** MSSVIHTLTLGVVVVLSVLSCGLGEDQVASVTDDKEQSDFSQRTLDSVHVLKKRAPGWGKRSLEDEVNSDDDMSYDSETPEDILSGADLSKRAPGWGKRTLDILEDYTKRAPGWGKRDSLDVKRAPGWGKRDIDMDKRAPGWGKRAPGWGKRAPGWGKRAPGWGKRAPGWGKRAPGWGKRSDTSCAGIDEEVDYYIYRAVQAEARRILECGSKYNGNDVLRK*

**CCAP**

MPSCRLLCLLLLPLLAVSASDDLEEDYVPQSKSSESHILSDPLSLPPDGRLMDGRLNLPRGWATLFDRLSSESSDQVRTSGDANAQTKRVFCNGFTGCGGRSRGRRSGPDGKSSESHILSDPLSLPPDGRLMDGRLNLPRAWTTLFDRLSSESSDQVRTPGNTDGLTKRVFCNGFTGCGGRFRGRRRQHPPVLGKRPFCNYFGCGNAGKRSEAPTKAVAQRVMRLPDDIRKRLFCNSFDGCRGRKRALYSNWLSKLQGIADDL*

**Cerebrin or PDF** MVSCSKYILILAFLLLACVSRDSSAAMIRPYQAQLEEKARQEVIHLAARIIKLTMYGSRLEMANKRNGGTLDSLFNLPDLSDNGR*

**Clionin** MSSVTLFSCACAMVLLVSVVTPSPTLPEEGLKDMGSEHACLFMCEMCFTGFPKAMLYCANEVCAAGTCNAPGMVWVGRTCKNPALLDKFLSK*

**Conopressin**

MVSASAMQVSAIVVLFGCVISGTLACFIRNCPPGGKRGLDMSGHNARECMACGPGGSGQCVGPNICCGSDIGCYFGTQEASICQKENESTTPCVIRGSTCGSRGQGHCVTDGVCCDEEACSFNSRCDVRASDRQTSKADLIKLVQQLLRSKNYD*

**CTP**

MDASSLVVFTTVILCLNYGHSAAKHQSEETVGVVINKLEQLLRDVSSLQHSVDQLDADIQSSLSPGKRSVRDTSSVMGHSRTTRSVEDLYRKRYMLRSLRAVLANTQRIIKAERKRSCNLNLGFHCQTDEYSSIADLYDFLSSDISPGKRKRTVGDSLNTS*

**Cysteine-knot protein hormone**

**(1)Bursicon A** MLPVRTVFLVCLLHVTSSDVMLPESGADRHEYYSAQETCRQQLRMTTIRFPGCAPKSIVTVSCRGTCHSRNVPEWRYNNQVMEMVEHCTCCKPHARRFRYIEFTCPARPGGKLRWRQWAALQCACRPCADAEPEAEQPYDY*

**(1)Bursicon-like** MWLKVIVLLLTVYILPTSGFVFNIRSSPDPNRNRCGLRWIKMTIHPPIMYASRCQSATVYTLGCKGGCESYSKIDYERNATNIQRYCSCCSQTEVAPIRKPALLPCSDGNVLRIPVKMALTCHCRPCSMSVQQIDIFDLRRLLASSRPSSAIFGG*

**(2)GPA2** MSLPMPQLPALSSLVLLLLTLYIVTLATSEVINPQTTISCHVRQYTMRVSKPHITESGDVIPCEGFVTVNSCWGRCDSSEIGDYKMPFKISHHPVCTYSGRAPRTVHLDNCPGHPDPEVEVFDAEGCACSLCNPEYTSCENLNG*

**Elevenin**

MMSHGVSYERVMVVLGLVLVAVSCVAQSRKIDCTRFVFAPRCRGVAAKRAQAAIMDAADSDDLSTLNKEPSLVDNDWNTDDTDSYQRLRFLAAKLGLRPVNVDNTAEQDLWERLMASRTMRH*

**ELH** MPRLYVCLAVLVYTCVSVNRISAEELKSKSKTEENYFSPGELKWPTGNGVYEDEIQALDEDGKGNVGRIAERENRSSGTSLWTKILNRIGKTQETFGSSGQYPNLPGKAKNERRYIAPQSVWAKLKRNQPEMPSFDAERAMNDGKFITQNKKRGLSINGALSSLANMLSAEGQRRDHAAALRLRQRLLVAGKR*

**FCAP**

MEWERYFRVSSLLLLSWAIVSCAPVEKSSGKDLKKREVNGHSMEHAMRLKMTKRSTSRMAYDDRELVDDLTEMGKRYLDSLGGAQIHGYKRFFDPLADVDVQTKRYLDPIGGFEVHGYKKRSASTAGRSARSRTKRALDTHGDSKIHDGLTRHTNKVDADKTHDGFKRGFDTLGGAQIHDGFKRG+

**FFamide** MNCKIPCLLLVLTLCILSITHAQANPRNLNRLVGQQPLLFGRRGVNPNMNSLFFGKRASNVNNMDVRTMCSAVLSACAAWQADMADN*

**FVamide**

**(1)Enterin** MSPIVEYLWKVSLAILFIHTVFVTGNDQTSDDTAAEKRRSPGFTHMFVGKRLPIELESRSPKYGYYFVGKRMPTAFQTRLSPKVGRWFVGKRPYAHRMFVGKRDMEMNDG+

**(2)MIP**

+MHDSGLLSPPPPHSAALQQGEVANPPPDGLARDAYMLSDDQGLERLLSSDSSNTDSSQPYSQLLDDELLQRIKQLVQQEQKRLAWNRRSYAPMFVGKKAAPLFIGKKSAPYFVGKKSAPYFVGKRAVPYFVGKKSAPYFVGKKAAPYFVGKKSAPYFVGKKA+

**(3)LRNFVamide** MPPLHQLLVAGLTLLVVSGGQADRDRRDVGATPDKRLREFFGKRDLSSADSAEGFGQDYGTYDKRVRDFFGKREDKRLREFFGKRSDNPDKRLREFFGKRSDPSADKRLREFFGKRSGDLSADKRLREFFGKRSDLSADKRLREFFGKRSDVTADKRLREFFGKRNGLEADKRLREFFGKRSDQPVEEKRLREFFGKRSGPEEQKRLREFFGKRDGHVDDKRLREFFGKRDGPMEEKRLREFFGKRNVIEEQKRLREFFGKRSVMPSPAAAKRKRDVRKRVREFIGKRSLLSFDDSIDNGYNKRVREFIG*

**FxRIamide** MTENVAQVLALVVAALVPMVMLVNKVQAAPTECADGLCDDKLAASDEAFSDGTDLDKRMSKFVRIGKGHGSSFVRIGRPHSFVRIGKSTGLESEYEEPAKRGGSNFLRIGKGMNRFLRIGKSVDSDKRASRFLRIGKSMEDEDEEMDKRGHAFVRIGKIPSSAFVRIGREPLLDRIMKKPSSSFLRIGRMGQSSFVRIGKKSSGSDEDTYSV*

**GGNG**

MDSQHSWCISFTALIYLALLLQPATGKCVGKWAIHSCFGGNGKRSDPSIAQTIDTQKQSNLLRQLLLRERFPPSVGLDMNEEQDREESDLNSFQDDLPQRPSAERQSDIDRLNIMLRTLMLQRKLRLAAERLA*

**GnRH**

MSVLSRQGVTVSVLLLLLTVHAVGGQNYHFSNGWHAGRKRGGDSTSCVFRKDILMLVNKLIMEESSRVAHRCQNGVPHMDFTEPDTVEADSANQLTDKRWK*

**Insulin1a** MDIAIAVSLALVLCLVCGVCPVNAYYEHTCYLDDRKPSVGGLCGRRASMMVDMVCRGSFGKRSVRFMDDDPSQEPVKFAAPKREALSYLGKRTSEQGFVCECCYHRCSIGELREYCHD*

**Insulin2** MKCWDKDILDKTVSRSAPSVEWAIRAVMVMVMRCPTVLLLLLCLQPGPAVADFEKTCNLEDRREGPAAGGICGSRLPEVIRLTCLVRRHRRSAKSLKGKAIDKQSELKDLTLHKSTALSYLLKRSNGLGEQGITCECCYNRCSLTELMQYC*

**Insulin4** MMNNVAFGLKSLHSTICVSSITVLALICLTKISPAFGDDDMMVRAFAERFRSLSGEELYDLWHTDCHRRCRAQLIEHINLACSYDPYKIISKRSVNSTVARKHVPDHDGDSEKKAPIFITRHASAAFLATGHKTKTVGHKIVKRGIMEECCYMKACSWEEYGEFCHTHNRQTTDRVTRCNK*

**Lymnokinin** MLSWKLPPARPKVGFAWVLVITAWASVASSLEAGITGLGSGEDEDLSAVSDKRAAFSAWAGKRDFLENFPESDSKEKRPAFSAWAGKRDYTEGDKRAAFSAWAGKRSLGGELDKRAAFNAWAGKRGTGGELLQDKRAPFSVWAGKRDFEGSFPEGKRAAFNAWAGKRSTPDDQELSGDKRAAFSSWAGKRDSGDSQSEATDEFESDKRAAFSSWAGKRSVTEARLNLKLLDWYLSRALAKVMEKRKFSVWAGKRDRYNNVFRRRFGPWSG*

**NKYamide** MQAYVTVAVLSAVLTLCTCMNIDQSRAALSGESRVKRQTLSDEYDVDSRMESLYSLLLARMAELSSRGTYPSTFSDVKRSSIGEIGLDNMPLTRKRKVFWQPLGYLPASARIGNQGSSSGAAKDKNGSSIFRYGK*

**Opioid** MRRLSVFLLVLLSMMAVHATDNAKSRSSEAAKVTQAPEMDDDNDDDEDEMGGLGWCKEDQRVAAICIACAQLPAALSLPPSECCSDADALEICKNCVDDPVRCLRDVLDIVTDGDRPQHEEEAMRAYDKRYGMLFFGQKPKYVPPRSDYVAEKRFRTLNLDNMGKRFDDQLDGELDKRWGSLGLSKRYGTLNLGSYFNKRYGMLGLGNRYLGYLGSRDPNMGKRFGTLNLGGLYSDMGKRYGTLNIGKESDLLSESELGKRYGTLNLGKRYGTLSMGRGSGPLSLGKRYGTLGMSNTFGYFPYKRSEYVPYNGAIGTDMKKRYGRLFMGRFGGKRLNSANEIRISQIKK*

**Pedal peptide A** MKLVSSLQPGLVLALGLCLVLGEKEEVKVGTSDGTSVAVKRSFDSINKGSGLSGFMKRGFDKISHGSGFSSFNKRPFDSISGSSAFSDFAKRPFDSIASGGGMAGFAKRPFDSISAGSGISGFAKRPYDSIGAGSGISEFAKRSFDSINAGSGLSGFAKRSFDSTGAGTGIAGFAKRPFDSIASGRGMSGFAKRPLESIAAGRDLSGFVKRPFDSIASGRGIAGFAKRPFDSISSGGGMAGFAKRPFDSISSGSGMSGFVKKSFDKIAHGGFASFNKKSDSEVDKRPFDSISQGAGMAGFAKRPFDSIGSGGFSTFVKKGLDSISHFSDMDDFGKRSVDNAGESESGERK*

**Pedal peptide B** +MSSFYKRGRDDVSGFYKRSPENDLSSFYKRAAGGDLSGFYKRSAGDDLSSFYKRAPGDDLSSFYKRGFQDSSSFYKRSRGDGLSNFYKRARDDVSGFYKRGLSNGLSSFYKRSVNDGVSSFYKRGLSDVNSFYKRGDDNNLSSFYKRNGDNLSSFYKRARNDLMGFYKRSGDDSNLSSFYKRARDELSGFYKRGLNDVSGFYKRSPAELSSFYKRMANGLSRFYKRSNEDLSGFYKRDTDVREDNELENVADKRSRDDVSGFYKRGLSNVSNFYKRDESEAL*

**Pedal peptide C**

METVKILFSVCVLLLLNGMVLCTEDDEETHELSKRSLDSVGSGFIKRPLDLVGSGFIKRVWNTDVGKRPIDQIGAGLIKRAMEQEEEEKRDLDYLGLGLLKREDDYEEEKRNLDRVGMGLIKRPIDTLGSGFIKKRPLDYLGSGLIRKRFFDPFAESLKKRPIDRIGSGLIKKRAIDELGMGLLKRPIDSIGSGFIKKRPIDSIGSGFIKKRPLDRIGSGFIRKRPIDSIGSGFIKKRPIDSIGSGFIKKRPIDSIGSGFI+

**PKYMDT** MDVTTTTVLGSLLLALLAVATSSGLPADEGRLDEASLAAEKRPKYMDTRQEDFEMVKQTVLIAMEELVQEGKLNPSVLAGGQGSKPVEKRMYMGICMRQSHNHFIPYPCMRSGRK*

**Pleurin** MMQLINPRRRMKESLVFLVFAVAGSCAGIFWTNSKDNDYPRIGRRSFYTASSENTYPRIGRSGGVEDTFRDGIERRASFFTQSDENTFPRIGRRNNDGSDVNKVEAKLQIPKGSPYPNSNEDTKSLTKPQTGLGTSFSLPAPILFFAWDANGDDSLSREEFVNGIANSRKRRHLV*

**RFamide**

**(1)FMRFamide**

+MRFGRAGEEEKRFMRFGRDGEDEKRFMRFGKSGEEEKRFMRFGRAGEDGEEFEDDDEGLEADKRFMRFGRDGEDEKRFMRFGKSGDDEKRFMRFGKRFMRFGRDGQDKRFMRFGRAGEEEKRFMRFGRDGEDEKRFMRFGKSGEEEKRFMRFGRAGEDGEEFEDDDEGLEADKRFMRFGRDGEDEKRFMRFGKSGDDEKRFMRFGKRFMRFGRDGQDKRFMRF+

**(2)LFRFamide** MDSSQILSILMLVFAMVLTGVALGAKEEQDSEAAVVAPSEHHKRSVDFPLSQDLLDDDAYDMDKRGSLFRFGKRGGLFRFGKRGTLFRFGKRGSTLFRFGRSGNDDLWVPVNEDGQDTKRNFHWGRETEE*

**(3)Luqin or cardioexcitatory peptide** MWKIMKLAEIVLCITTVLLLTFTTGNGAPQWRPQGRFGKRVQNNLPLLINEDSSNHSPEPASSPYEIPIEKLETHSDGSVLRLLDRLCTESNVPGFRKCLWNKRK*

**(4)NPY**

QKLVIAVLLVFSLVVMEVTCQDAMLAPPDRPSEFRSPDQLRQYLKALNEYYAIVGRPRFGRSVNKRSVNDAVFGEATKTE*

**Tachykinin1** MSSSQGVIFVLAGFALLALVNAEALDDNTAASLYKLLQPAYQSTVAEKRESDLNLAFDAPPDPNWSAGPSTYGFGSLLRRAPAGYRRNLIEDKRFGYVGSRGKRTELGFGYVGSRGKRTNHATAFVTLRDLLDMYEERARGLPFRASDLNSLSQDSALLNSKRQPHFGFHGVRG*

**Tachykinin2** MWKVSTLCIVLVCGFVGTQAEKETHDESVKHKGKINFTEGSSLTSNPSPTHNTQEDPDAFMDSLLGYRIPLSTDDNELKKRGRHFGFVGSRGRRGKTSVNSFSEDLFPKRRPMSNSARLRMLLENRDQGLFAKEKRKPHFGFHGSRG*
