# Supplementary material for: Neural Ganglia Transcriptome and Peptidome Associated with Sexual Maturation in Female Pacific Abalone (Haliotis discus hannai)
Source: Genes (Basel). 2019 Apr 2;10(4):268. doi: 10.3390/genes10040268 (PMC6523705; doi:10.3390/genes10040268)
Supplement: Supplementary file 1 [file genes-10-00268-s001.zip › Supplementary File 3.docx]

MAP-1 (TBIU010983)

MQILTFSWILLAVSPVIISGQLEKQIGSKRSVILTSILLQERRYDRMSKRRDSVVSRPMPYECCPALRERIEPYGGISREGKVLELYRDSRTVQTFYQTRCRPWVVNGQCHYLDVRAKRYSRCVQKYTYMYGIVKDYNVTQPYRVDYIKVKSGCTCELDFHRDATRYG

MAP-2 (TBIU005609)

MASTPALYTLAVLFVTCIVITTHGFSLKGFCASNCARGKGGNVCKCNGFHFAGKRGVPTLDSDLLTDSMGDSKEENFLELDSEGFGVFGYPKDDSRRRTLKNTNSRGNFRALLEKYLAERYSRGGGFGPASNPDSWSEVYRTGN

MAP-3 (TBIU009796)

MVRKKDLGKVFFIYLCLFSVCPAHPCGENPLRGFMSLSRITGKNVNHLLGILRSANTDLYGRLERNWQLYANCVGMVDTGYFKRSGSAAKKAGIANQVTRILPIQVLSPDDLMYYDRSNTEDDSLL

MAP-4 (TBIU003144)

MEVTRLVSVCLSVLVLAQVIVAHPYRSRDTDTALGDDVTNLAQDLVRLFEDARRENSDNSDERRAKRYVHFNIGNDHQVSRRSFRNRRIHHDRRRARGGFQVSRV

MAP-5 (TBIU013612)

MTWRDCVLPHISSTFRSSMKQSGAALSALLPLVVLLSHTGLSFARSLSLDDTYWVDNVDKRNSWFSKKSLDEFGPLSNAASAISQQTEGDHSRQLYSCYVETCVPDFIICAKRSRTQRGFSMCKMDHRVCAVECWTKTSKETGA

MAP-6 (TBIU017101)

MQNSWMAFTVVAVATLCVCVSGMGIGKGAQSFGDGQNNDQRYQKWYEWMVKRQLGSNDMSFGRYLQDGGKFLDWGSLFDGGRGKRQLSGFPDKFGNYQRFFTNGLQDWKSTFNGEKRVAPEE

MAP-7 (TBIU032784)

MQFRRSLQVCVVFATLVSLSQSFRRDVREGSDLFSSLSSVEAKRSTIFDRMGRFYGKRSSTEMEGMNGKIRPLYTTEEMRELVSGSPRLLAGIIRGYIDRNGDDLIDEEEMSQLKKRIQYQ

MAP-8 (TBIU035555)

MPEWSRMLAYVFAVAALSQCLIKPTDSTETRAAFWDNAAGRQPYKKRDMQSLSALLSPPDRSDVTVEDVADLISQVPAMAKALVQRFVDRDGDGLISEEELMDLWK
